# Genomic repeats, misassembly and reannotation: a case study with long-read resequencing of *Porphyromonas gingivalis* reference strains

**DOI:** 10.1186/s12864-017-4429-4

**Published:** 2018-01-16

**Authors:** Luis Acuña-Amador, Aline Primot, Edouard Cadieu, Alain Roulet, Frédérique Barloy-Hubler

**Affiliations:** 1 Institut de Génétique et Développement de Rennes, CNRS, UMR6290, Université de Rennes 1, Rennes, France; 2 Laboratorio de Investigación en Bacteriología Anaerobia, Centro de Investigación en Enfermedades Tropicales, Facultad de Microbiología, Universidad de Costa Rica, San José, Costa Rica; 3 GenoToul Genome & Transcriptome (GeT-PlaGe), INRA, US1426, Castanet-Tolosan, France

**Keywords:** *Porphyromonas gingivalis*, Bacteroidetes, long-read sequencing, misassembly, genomic repeats, annotation, biocuration, comparative genomics

## Abstract

**Background:**

Without knowledge of their genomic sequences, it is impossible to make functional models of the bacteria that make up human and animal microbiota. Unfortunately, the vast majority of publicly available genomes are only working drafts, an incompleteness that causes numerous problems and constitutes a major obstacle to genotypic and phenotypic interpretation. In this work, we began with an example from the class Bacteroidia in the phylum Bacteroidetes, which is preponderant among human orodigestive microbiota. We successfully identify the genetic loci responsible for assembly breaks and misassemblies and demonstrate the importance and usefulness of long-read sequencing and curated reannotation.

**Results:**

We showed that the fragmentation in Bacteroidia draft genomes assembled from massively parallel sequencing linearly correlates with genomic repeats of the same or greater size than the reads. We also demonstrated that some of these repeats, especially the long ones, correspond to misassembled loci in three reference *Porphyromonas gingivalis* genomes marked as circularized (thus complete or finished). We prove that even at modest coverage (30X), long-read resequencing together with PCR contiguity verification (*rrn* operons and an integrative and conjugative element or ICE) can be used to identify and correct the wrongly combined or assembled regions. Finally, although time-consuming and labor-intensive, consistent manual biocuration of three *P. gingivalis* strains allowed us to compare and correct the existing genomic annotations, resulting in a more accurate interpretation of the genomic differences among these strains.

**Conclusions:**

In this study, we demonstrate the usefulness and importance of long-read sequencing in verifying published genomes (even when complete) and generating assemblies for new bacterial strains/species with high genomic plasticity. We also show that when combined with biological validation processes and diligent biocurated annotation, this strategy helps reduce the propagation of errors in shared databases, thus limiting false conclusions based on incomplete or misleading information.

**Electronic supplementary material:**

The online version of this article (10.1186/s12864-017-4429-4) contains supplementary material, which is available to authorized users.

## Background

Pioneer studies such as the MetaHIT consortium [1] and the Human Microbiome Project [2] have used high-throughput sequencing techniques to produce a detailed catalog of human-associated bacterial taxa. The vast majority of prokaryotes identified in and on human beings belong to only four phyla: Actinobacteria, Bacteroidetes, Firmicutes, and Proteobacteria [3]. To date, the best-described human microbial community is the gut microbiota [4], which is mostly (~90%) composed of members of the phyla Bacteroidetes and Firmicutes [5, 6]. Among these, the dominant classes are the strict anaerobes Bacteroidia and Clostridia, respectively [7, 8]. Although their relative proportions may vary [9, 10], Bacteroidetes make up approximately 50% of the gut microbiome [11].

The phylum Bacteroidetes (or “Bacteroidaeota” as recently proposed [12]), is highly diverse, and its phylogenetics has been well explored [13–15]. The Bacteroidia are Gram-negative chemoorganotrophic rod-shaped organisms. Either non-motile or moving by gliding, they have colonized several ecological niches, including soil, oceans, fresh water and the abovementioned gastrointestinal tract [8]. Their genomes can undergo massive reorganization, with extensive and frequent horizontal gene transfers (HGTs), and the sizes of their genomes correlate with their functional specialization [14]. The class Bacteroidia includes some commensal genera that can present as opportunistic pathogens, such as the intestinal *Bacteroides* and the oral *Prevotella*, *Porphyromonas,* and *Tannerella* [8, 14, 15].

Approximately 10 years ago, the technological breakthrough known as next-generation sequencing (NGS, now also called SGS for second-generation methods or massively parallel sequencing) exponentially lowered sequencing costs, making these techniques widely accessible [16]. In recent years, massively parallel sequencing has almost entirely been conducted using Illumina’s MiSeq and HiSeq platforms [17, 18]. Prior to these developments, most whole-genome sequencing (WGS) projects were conducted on organisms that were selected due to their relevance to medicine or biotechnology [19], resulting in a strongly biased portrait of microbial diversity [20]. Researchers such as those involved in the Genomic Encyclopedia of Bacteria and Archaea (GEBA) are currently attempting to compensate for this by sequencing at least one species from each known genus [19].

WGS generates primary information and a catalog of reference genomes. The associated biological information is typically stored in online databases and used for downstream purposes such as comparative genomics, transcriptomics, and proteomics [16, 21–23]. For genome assembly using massively parallel methods, computation time and memory efficiency have led to the use of algorithms based on de Bruijn graphs [24]. In this method, a genome is constructed using graphs, but if the assembly software encounters a genomic repeat that is equal to or longer than the read length, it either continues the assembly by guessing (which can create false joins) or breaks it, leaving repeat-induced gaps [25–27]. These assembly breaks are common because genomic repeats in bacteria account for 5 to 10% of the total genome. Their frequency is variable but not adaptively neutral since repeated DNA sequences are involved in protein-DNA interactions, bacterial immunity, specialization, speciation and transcriptional regulation [28, 29].

The expected outcome of WGS is the complete DNA sequence of a genome. An initial draft sequence is usually obtained in a matter of days and then completed through genome finishing, a cost-intensive process that may require months or even years [30]. For some bacteria, assembly finishing may be the most important step in genome sequencing, and there are at least three strategies for gap closure and assembly validation. The first of these strategies is reference-assisted gap closure, which consists of arranging contigs into a putative chromosome using information from a closely related complete genome [31, 32]. This assumes that the published reference sequences are accurate [33], biologically validated and closely phylogenetically related [34]. The second strategy involves long read (re)sequencing using either mate-pair libraries [35] or long-read techniques such as PacBio [36] and Nanopore [37]. The third strategy is based on genome maps [38, 39]. These methodologies incur additional cost and require significant time investments. In fact, in a WGS project, assembly finishing comprises over 95% of the total cost and timeframe. Therefore, researchers often decide that finishing is not cost-effective, preferring to publish the draft genomes, which abound in the databases [40]. The accumulation of numerous draft genomes creates massive databases; unfortunately, the quality of the genomes is decreased since it is based on incomplete and/or incorrect genomic data [41, 42]. Indeed, draft genome assemblies are not sufficient for studying large-scale genome architecture [43], can result in incomplete or incorrectly annotated genes (e.g., partitioned annotation of ORFs), and may hinder evolutionary studies [44, 45] by leaving significant portions of the genome unclear or inaccurate [17]. Furthermore, some studies have demonstrated the limitations and difficulties associated with using drafts for studies involving HGT analysis, phylogenomics, the evolution of genome synteny, genome structural analysis, and pangenomic approaches [34, 42, 46, 47].

In this study, we focused on Bacteroidetes genomes because of the importance of this phylum in microbiota communities but also because this clade contains genomes that are mostly available as drafts. We attempted to identify the reasons for this incompleteness. We first explored the number of published genomes in Bacteria and determined the percentage of genomes released as drafts. We then studied species in which at least two strains have been sequenced, since this is required for comparative analysis of their repeats. We followed this with *in silico* sequencing to elucidate the links between assembly and repetitions. Finally, we narrowed in on the genomic diversity of an interesting model in this clade, *Porphyromonas gingivalis*. Using three principal reference strains, we combined long-read resequencing and *de novo* assembly with manually biocurated annotations. In this way, we produced correct and consistent genome sequences that are valuable resources for future comparative genomic studies.

## Methods

### Analysis of the NCBI genome database

On 3 April 2017, we accessed all data through 2016 in the NCBI genome database (https://www.ncbi.nlm.nih.gov/genome/browse). For analysis purposes, we considered the "FCB Bacteroidetes/Chlorobi," "Terrabacteria Firmicutes," "Terrabacteria Actinobacteria," and "All Proteobacteria" subgroups to represent the phyla Bacteroidetes, Firmicutes, Actinobacteria, and Proteobacteria, respectively. The levels "Complete" and "Chromosome" were considered finished or complete genomes, and "Scaffolds" and "Contigs" were considered draft or incomplete genomes. Analysis was performed using R (v3.3.2) [48] and in-house parsing scripts in Python (v2.7.10). We excluded all entries marked “Candidatus” and entries that did not include full species identification specifying both the genus and the species.

### Analysis of Bacteroidetes genomes

Bacteroidetes genomes were categorized by class and sequencing status. The sequences of all copies of genes encoding 16S rRNA were extracted from the complete genomes. Alignment was performed using the MAFFT (v7.222) plug-in [49] in Geneious (v10.2.3) [50] set to the following parameters: automatic detection for the algorithm; a scoring matrix of 200 PAM/k = 2; a gap opening penalty of 1.53; and an offset value of 0.123. A consensus sequence was then created for each species using the default Geneious settings. Finally, a phylogenetic tree was constructed using the PhyML plug-in [51]. The tree was based on an HKY85 substitution model [52] and features 100-bootstrap branch support; optimized topology, branch lengths, rates, and nearest-neighbor interchange (NNI); and a subtree pruning and regrafting (SPR) topology search. The tree was simplified to the class level according to the NCBI taxonomy. The isolation sources of all complete genomes were identified via the NCBI BioProject and BioSample databases. The sources were classified into three categories: environmental (soil, fresh or marine water, sludge, mud, plants and algae samples), animal (insects, mollusks, fish, birds, cattle, and domestic animals), and human (isolated from various body sites of healthy or sick individuals).

### Complete Bacteroidia genomes and genomic repeats

Genomes of species of the class Bacteroidia were classified by sequencing status ("Complete" or "Draft") and by year of publication. The number of contigs in draft genomes was analyzed by genus. We retrieved the complete genomes (those having no assembly gaps) of all species identified as having only one chromosome in the NCBI database. If plasmids existed, only the chromosome was analyzed. Species with two or more chromosomes were excluded (Additional file 1: Table S1). For further analysis, the complete genomes of species for which genomic sequences of two or more strains were available were studied; these included the four *Bacteroides* species *B. dorei, B. fragilis, B. ovatus,* and *B. thetaiotaomicron*, *Porphyromonas gingivalis*, and *Tannerella forsythia*. We studied the number of contigs present in draft assemblies for those species (Table 1). The genome-based similarity measure OrthoANI was used to assess intra- and inter-species relatedness [53].

**Table 1:** Information on the complete genomes used in this study

Species	Strain	Sequencing Technology	Assembler	Coverage	GenBank assembly accession
*Bacteroides dorei*	HS1_L_1_B_010	PacBio	Celera	306	GCA_000738045
*Bacteroides dorei*	HS1_L_3_B_079	PacBio	Celera	370	GCA_000738065
*Bacteroides dorei*	HS2_L_2_B_045b	PacBio	Celera	185	GCA_001274835
*Bacteroides dorei*	CL03T12C01	PacBio	SMRT Analysis	193	GCA_001640865
*Bacteroides fragilis*	NCTC 9343	Sanger	Phrap	10	GCA_000025985
*Bacteroides fragilis*	638R	Sanger	Phrap	9	GCA_000210835
*Bacteroides fragilis*	BE1	Illumina + Nanopore	SPAdes	68 + 8	GCA_001286525
*Bacteroides fragilis*	BOB25	454 + IonTorrent + Sanger	Newbler	29	GCA_000965785
*Bacteroides fragilis*	S14	Illumina	CLC-GW + SPAdes	73	GCA_001682215
*Bacteroides fragilis*	YCH46	Sanger	Pherd/Phrap	10	GCA_000009925
*Bacteroides ovatus*	ATCC 8483	PacBio + Illumina	HGAP + Celera	350	GCA_001314995
*Bacteroides ovatus*	V975	454 + Sanger	Newbler	23	GCA_900095495
*Bacteroides thetaiotaomicron*	7330	PacBio + Illumina	HGAP + Celera	395	GCA_001314975
*Bacteroides thetaiotaomicron*	VPI-5482	Sanger	Phrap	7	GCA_000011065
*Porphyromonas gingivalis*	381	454 + Sanger	Velvet + Newbler	50	GCA_001314265
*Porphyromonas gingivalis*	A7436	454 + Sanger	Velvet + Newbler	57	GCA_001263815
*Porphyromonas gingivalis*	A7A1-28	454 + Sanger	Velvet + Newbler	94	GCA_001444325
*Porphyromonas gingivalis*	AJW4	454 + Sanger	Velvet + Newbler	60	GCA_001274615
*Porphyromonas gingivalis*	ATCC 33277	Sanger	Pherd/Phrap	9.5	GCA_000010505
*Porphyromonas gingivalis*	TDC60	454 + Sanger	Newble + Phrap	9 + 7	GCA_000270225
*Porphyromonas gingivalis*	W83	Sanger	TIGR Assembler	8	GCA_000007585
*Tannerella forsythia*	3313	Sanger + 454 + Illumina	Newbler	21	GCA_001547875
*Tannerella forsythia*	92A2	Sanger	Celera	12	GCA_000238215
*Tannerella forsythia*	KS16	Sanger + 454 + Illumina	Newbler	23	GCA_001547855

For complete genomes, we used Repeatoire to identify genomic repetitions that were at least 95% similar to each other and longer than 500 base pairs (bp) [54], visualizing them with Circos (v0.69) [55]. In each genome, the repeat’s initial location was fixed as the starting point for all of the links to the other positions of that repeat, and we color-coded the copy numbers of each repetition.

### Simulated read assembly

For each genome, artificial reads were produced using ART software (v2.5.8) [56] set for paired-end reads of 250 nucleotides (nt) each, 500 nt insert size, and a coverage of 40; the built-in MiSeq simulation profile (v1) was used. For the *P. gingivalis* genomes, eleven *de novo* assemblers were tested using the default parameters: A5-miseq (v20160825) [57]; CodonCode Aligner (CCA v7.0.1); CLC Genomics Workbench (CLC-GW v8.5.1); fermi (v1.1) [58]; Geneious (v10.2.3) [50]; Minia (v2.0.3) [59]; MIRA (v4.0.2) [60]; PERGA (v0.5.03.02) [61]; SOAPdenovo (v2.04) [62]; SPAdes (v3.10.1) [63]; and Velvet (v1.2.10) [64]. For CCA and Geneious, reads were preassembled with PEAR (v0.9.10) [65]. Where required, KmerGenie (v1.7016) was used to determine the *k-mer* length parameter [66].

After the initial test step, we selected three software packages for assembly of all 24 genomes. The first was A5-miseq, in which we used the default parameters. For Geneious, reads were preassembled with PEAR and then assembled using the default Medium Sensitivity/Fast setting and generating consensus with a 50% strict threshold for calling bases. Finally, SPAdes was used with the default parameters but with the “careful” option and *k-mer* lengths of 21 to 127.

For each genome and assembly tool, we rejected contigs of less than 1 Kbp because they are not informative. We assessed the assemblies with QUAST (v4.5) [67]. The fidelity of each assembly was evaluated by mapping each set of contigs to the reference genome using Geneious mapper (Medium Sensitivity/Fast option); the unmapped contigs were rejected, and QUAST was then used again. The correlation between the number of repeats (copy number > 3) and each assembly was tested using the “ggplot2” and “nortest” packages in R. We identified the *P. gingivalis* reference genome segments that correspond to assembly gaps and classified these into five categories: genomic islands, *rrn* operons, coding sequences (CDS) with repeated domains, intergenic sequences, and insertion sequences or miniature inverted-repeat transposable elements (IS/MITE).

### Bacterial strain cultures and DNA extraction

We purchased the ATCC 33277 and W83 (also known as BAA-308) *P. gingivalis* strains from the American Type Culture Collection-LGC Standards (Manassas, VA, USA) in September 2006; a low (< 20) passage number was used. *P. gingivalis* TDC60 (also known as JCM 19600) was purchased from the Japan Collection of Microorganisms (Riken BioResource Center, Koyadai, Japan) in November 2015, and a low (< 10) passage number was used. All strains were cultured on Columbia European Pharmacopoeia agar plates (Conda, Madrid, Spain) supplemented with 5% (v/v) defibrinated horse blood (Eurobio, Courtaboeuf, France), 5 g/L yeast extract (Conda), 25 mg/L hemin (Sigma-Aldrich, Saint-Quentin Fallavier, France), and 10 mg/L menadione (Sigma-Aldrich). The cultures were incubated in an anaerobic chamber in a Whitley DG500 Workstation (Don Whitley Scientific, Shipley, UK) for 5 days at 37 °C in an atmosphere composed of 80% N_2_, 10% H_2_, and 10% CO_2_.

For DNA extraction, each strain was cultured for 48 h at 37 °C under the same atmospheric conditions described above. This was done in 50 mL BHI broth (BioMérieux, Marcy l'Etoile, France) enriched with 5 g/L yeast extract (Conda), 25 mg/L hemin (Sigma-Aldrich), and 10 mg menadione (Sigma-Aldrich). After harvesting, the cells were washed twice in Dulbecco's PBS (Dominique Dutscher, Brumath, France). A QIAamp DNA Mini Kit (QIAGEN, Courtaboeuf, France) was used for cell lysis and protein denaturation. The following steps were performed using standard methods: DNA precipitation with 5 mol/L NaCl (Sigma-Aldrich) and 0.7 volumes of cold isopropanol (VWR Chemicals, Fontenay-sous-Bois, France), two washes in 70° ethanol (WWR), and resuspension in sterile Milli-Q ultrapure water (Merck, Darmstadt, Germany).

### DNA library preparation and PacBio SMRT sequencing

Barcoded DNA library preparation and single molecule real-time (SMRT) sequencing were performed using the Genome et Transcriptome (GeT) GénoToul platform (Toulouse, France) according to the manufacturers’ instructions. Quality control was performed at each step. DNA was purified using AMPure PB beads (Pacific Biosciences, Menlo Park, CA, USA), and the mass of dsDNA was verified using a Qubit fluorometer (Thermo Fisher Scientific, Villebon sur Yvette, France). The purity of the DNA was determined based on absorbance ratio using a Nanodrop spectrophotometer (Thermo Fisher Scientific). Sizing measurements were performed using a Fragment Analyzer (Advanced Analytical Technologies, Evry, France). In brief, each sample was diluted to 10 μg/mL and sheared on a Megaruptor (Diagenode, Seraing, Belgium). Using 5 μM of various barcoded adapters, a SMRTbell Barcoded Adapter Prep Kit (Pac Bio) was used to repair and ligate 150 ng of DNA fragments. After end-repair and ligation using a SMRTbell DNA Damage Repair Kit, the samples were pooled. To remove unligated DNA fragments, the library was treated with an exonuclease cocktail consisting of 1.81 U/μL Exo III and 0.18 U/μL Exo VII (PacBio).

Library selection in the 6-50 Kbp range was performed using BluePippin 0.75% agarose cassettes (Sage Science, Beverly, MA, USA). Primers were annealed to the size-selected SMRTbell with the full-length libraries. The primer-template complex was then bound to the P5 enzyme using a 10:1 ratio of polymerase:SMRTbell for 4 h at 30 °C. The magnetic bead loading step was conducted at 4 °C for 1 h. The complexes were placed into the PacBio RS II sequencer, which was configured to run continuously for 6 h at a sequencing concentration of 80 pM. The sequencing results were validated using the NG6 integrated next-generation sequencing storage and processing environment [68].

### Genome assembly and finishing strategy

We mapped the contigs obtained from the *in silico* reads to each reference genome. We identified and retrieved the sequences not covered by the contigs, which corresponded to the assembly gaps. Because these gaps are repeated regions, we clustered the sequences at 99% nucleotide identity and generated a consensus sequence. Thus, each consensus represents one type of repeated region. We used canu (v1.3) to correct, trim, and assemble the raw reads [69]. We mapped the corrected long reads to each consensus sequence that was previously identified; for each, we selected only reads overhanging at least 500 nt at both ends. The selected reads were *de novo* assembled using Geneious at 100% nucleotide identity. They were then used to scaffold the contigs generated by canu and to finish the assembly and reconstruct the genome organization.

To confirm our construction, PCR was used on repeats longer than the median trimmed/corrected read length (i.e., *rrn* operons and CTnPg1). The primers used are listed in Table 2. For the *rrn* operons, 22 additional *P. gingivalis* strains were tested; 16 of these were isolated from Colombian patients with periodontitis (UIBO421B, UIBO465, UIBO472, UIBO537B, UIBO655H4, UIBO695H2, UIBO710B, UIBO728B, UIBO728H3, UIBO742, UIBO760B, UIBO771H2, UIBO783, UIBO801H3, UIBO1047B, and UIBO1047H [70]), 4 were isolated from French patients with periodontitis (2J14, M71, MAJ and TN), and 2 strains (OMZ314 and OMZ409) were provided by Prof. J. Gmür of Zurich, Switzerland. For all PCR reactions, we used 50 ng genomic DNA, 1X Phusion GC buffer, 7% DMSO, 0.02 U/μL Phusion Hot Start II High-Fidelity DNA Polymerase (Thermo Fisher), and 200 nM of each primer (Eurogentec, Seraing, Belgium). PCR was performed under the following conditions: an initial denaturation step at 98 °C for 3 min; 30 cycles of 98 °C for 20 s, 63 °C for 30 s, and 72 °C for 7 min 30 s; and a final elongation step at 72 °C for 10 min. The CTnPg1 validation was performed under the same conditions as the *rrn* PCR except that no DMSO was used and the annealing temperature was 67 °C.

**Table 2:** Primers used to validate the architecture of the *Porphyromonas gingivalis* genome

Primer Name	Primer sequence (5' to 3')	Tm (°C)
rrn1F	TCCCCACCGGCAAAAACATC	68.0
rrn1R	GAGATGTCCGAAAGTCCATGTCAC	66.3
rrn2F	AGATAGCCAGTTTCGTTACGTCCG	67.3
rrn2R	TACAGCAACGGTTACTTCCGCG	68.6
rrn3F	CTATGGATATTCTGCGGTGTACGG	66.3
rrn3R	GTTGTAGGACAGCAACCTTTTGG	64.2
rrn4F	ACAAGTCAGAACATGGCCGAT	64.3
rrn4R	CAGGCACAAACCGCTTTACC	65.0
ctnpg1_5out1	GACGGAATTTGCGTGTTGATATAGT	64.3
ctnpg1_5out2	ATAAACGTGTGGCCGAAATAGATTC	65.3
ctnpg1_5in	CAATAGCGTTTGCATTACCTCATCT	65.3
ctnpg1_mid	ATCGGTGGAGATGTTCATACTACTG	63.9
ctnpg1_3in	GTATTTGCCCAATACTCTCTGAACG	64.9
ctnpg1_3out1	CGACAACATCGTATTTCTCTGTCAG	64.9
ctnpg1_3out2	CACCGAGATTCAAGGTTATGTGATG	66.9

### Genome annotation and biocuration

Genomes were annotated using Prokka (v1.12-beta) [71], Genix online [72], and RASTtk (v1.3.0) [73]. The annotations for the gene starts/ends, gene names, gene product descriptions, gene status (gene, pseudogene by stop in-frame or frame-shift), EC numbers, and functional descriptions were all manually biocurated [74]. For this purpose, we performed NCBI BLAST searches [75] against non-redundant databases as well as domain searches using the Conserved Domains search tool [76]. The results were then compared to the precomputed annotations from MicroScope [77].

The presence of signal peptides was analyzed using the SignalP 4.1 server [78] and SOSUIsignal [79]. Protein subcellular localization predictions were made using PSORTb (v3.0) [80] and CELLO (v2.5) [81]. The outer membrane proteins predicted by these tools were then confirmed using BOMP [82], and LipoP (v1.0) [83] and DOLOP [84] were used to predict the lipoproteins.

Insertion sequence transposases were renamed as per the ISfinder [85] nomenclature. Clustered Regularly Interspaced Short Palindromic Repeats (CRISPRs) were identified using CRISPRfinder [86], CRISPRDetect [87], CRISPI [88] webtools and the CRT plug-in [89] with Geneious. We predicted genomic islands using IslandViewer (v4) [90], which has precomputed predictions from SIGI-HMM [91], IslandPath [92], and IslandPick [93]. Island prediction was also performed using EGID [94], which implements AlienHunter [95], SIGI-HMM, IslandPath, INDeGenIUS [96], and PAI-IDA [97].

All genomic sequences and the reads used to produce them were deposited in GenBank and in the NCBI BioProject database with links to the BioProject accession number PRJNA393092.

### Comparative genomics

Genome-level comparisons were made with progressiveMauve [98] using the default settings. The same program was used to identify locally collinear blocks (LCBs) and single-nucleotide polymorphisms (SNPs).

For each strain, we compared the available annotation in the NCBI database to our manually biocurated annotations feature-by-feature: rRNA; tRNA; tmRNA; ncRNA; regulatory; repeat region; and coding DNA sequence (CDS). The CDS were separated into pseudogenes and “true” coding regions. For the latter category, the CDS/pseudogenes present in both annotation versions were termed “common,” and those that were present only in our new version were called “new.” We noted all instances of changes from a CDS to a pseudogene and vice versa, fusion (more than one interval becoming one interval) and separation (one interval becoming more than one interval), and changes in the coding strand. Finally, we also analyzed the changes in start/stop codons.

We concluded our study with a pangenomic analysis of the *P. gingivalis* strains ATCC 33277, TDC60 and W83. The CDS and pseudogenes were classified into six groups: multiple-copy genes (nucleoid-associated proteins, *tra* genes, *xer* genes, and transposases in ISPg); those that appeared in only one copy in all of the strains and were highly conserved (> 97% nucleotide identity); those present in only one copy in all of the strains but with sequence divergence (< 97% nucleotide identity); those present in all three strains but present in more than one copy in at least one strain and/or having pseudogenes; those present only in two strains and either copied or with pseudogenes; and those present in only one strain.

## Results

### The Bacteroidetes phylum is represented by few genomes, mostly incomplete and with variable numbers of contigs

Bacterial genomes represent approximately 85% of the available genomes in the NCBI genome database. Despite the fact that Proteobacteria represents a relatively minor constituent of human microbiota, it is the most sequenced bacterial phylum, even in comparison to the two most abundant phyla, Bacteroidetes and Firmicutes (Additional file 2: Figure S1a). Furthermore, the accumulation of incomplete draft bacterial genomes is notable, comprising 85-95% of all entries depending on the phylum (Additional file 2: Figure S1b).

Within the phylum Bacteroidetes, the classes Bacteroidia and Flavobacteriia constitute 90% of the listed genomes. Flavobacteriia is mostly associated with environmentally obtained samples, particularly with aquatic species such as water and fish pathogens, and rarely (18% of complete genomes) with humans. In contrast, Bacteroidia isolation sources are human in more than 75% of cases (Additional file 3: Figure S2a). Although generally considered part of the normal microbiota, Bacteroidia are pathobionts that can become pathogens upon dysbiosis. Prior to the introduction of massively parallel sequencing, only four complete Bacteroidia genomes were published. Since 2007, this number has grown exponentially, mainly due to the large number of draft genomes that have accumulated to such an extent that today 10 times as many draft genomes as complete genomes are available (Additional file 3: Figure S2b).

In addition to the preponderance of draft genomes, the Bacteroidia draft genomes are extremely fragmented; half of them contain more than 75 contigs. The number of contigs per draft ranges widely from 2 in *Prevotella oryzae* DSM17970 to 4357 in *Bacteroides acidifaciens* 1a3B. Observing the number of contigs per draft in bacterial genera with at least five draft genomes, we noted that 50% of all entries are *Bacteroides* and that *Bacteroides*, *Parabacteroides,* and *Prevotella* all have at least one draft genome featuring more than 500 contigs. Based on their interquartile ranges (IQRs), the genera with less variation were found to be *Alistipes* and *Tannerella*; coincidentally, these were the genera with the smallest number of draft genomes. At 93 contigs per draft, *Bacteroides* and *Tannerella* have the highest median number of draft genomes (Additional file 4: Figure S3a). Even when only species having at least two complete genomes (*B. dorei*, *B. fragilis*, *B. ovatus*, *B. thetaiotaomicron*, *P. gingivalis*, and *T. forsythia*) are considered, this variability is still quite large. *B. fragilis* represents almost 20% of all Bacteroidia drafts and is the most variable in terms of the number of contigs per draft. *B. dorei* and *T. forsythia*, although the species with the smallest number of drafts, are the least variable. Finally, *P. gingivalis* has an intermediate profile (Additional file 4: Figure S3b).

### Draft variability cannot be explained by sequencing and assembly methods

Several hypotheses could explain the observed variability in the features of the draft genomes. It could be linked to differences in the assembly strategies (algorithm types or specific assembly tools) and/or the massively parallel technologies used. To test these hypotheses, we collected all of the available information on sequencing and assembly methodology for all of the draft genomes of the six species studied here (Additional file 5: Table S2). Of the 166 drafts, 144 were sequenced using Illumina technology, and only six assemblers were used for *de novo* assembly. Five assemblers used the De Bruijn graphs (Allpaths, Velvet, ABySS, CLC Genomics Workbench), covering 40% of the drafts. The final MaSuRCA assembler, which is based upon the de Bruijn and Overlap-Layout-Consensus (OLC) approaches, was used for the remaining 60% of the drafts. However, the overrepresentation of MaSuRCA is due to a single sequencing project of different strains of *B. fragilis* at the Institute for Genome Sciences (University of Maryland, Baltimore, MD, USA). Once again, the results (whether per assembler or per bacterial species) were very diverse. In *B. fragilis* projects using Illumina for which an assembler was specified (99 of 107 drafts), MaSuRCA generated 31 to 2566 contigs (*n* = 69), Allpaths produced 5-14 contigs (*n* = 10), SPAdes generated 73-343 (*n* = 8), ABySS produced 150-1290 (*n* = 6), CLC Genomics Workbench produced 5-156 (*n* = 5), and Velvet, which was only used once, generated 52 contigs. The 20 *P. gingivalis* draft genomes were sequenced using Illumina technology and then assembled 17 times with Velvet (generating 22-192 contigs) and once each with SPAdes (92 contigs), Celera (104 contigs), and SOAPdenovo (117 contigs). For all other species, the number of drafts with known assemblers is too small (< 10) to draw conclusions. At this point, it therefore seems that technological differences in sequencing and assembly cannot explain the extensive differences in the number of contigs per draft.

The hypothesis that intra-species diversity might be responsible for this variability remains to be explored. Because this hypothesis involves comparative genomics, only complete genomes could be used to explore its validity.

### Complete *Porphyromonas gingivalis* genomes are the most diverse and repeated genomes within Bacteroidia

**Fig. 1:**
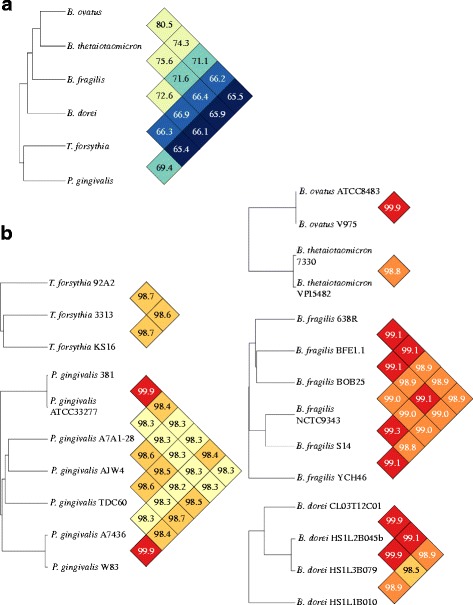
Relatedness of complete Bacteroidia genomes for species having at least two different strains. **a.** Dendrogram of the inter-species relatedness calculated with the OrthoANI algorithm, clustered using UPGMA, and shown with the corresponding pairwise identity heatmap. **b.** Dendrogram of the intra-species relatedness, shown with the corresponding pairwise identity heatmap

By calculating the average nucleotide identity (ANI), we separated two groups of species. The first group contained the *Bacteroides* genus, whereas the second group included *P. gingivalis* and *T. forsythia* (Fig. 1a). ANI values offer a robust and sensitive way to measure the evolutionary relationship of bacterial strains [99]. With the exception of the *P. gingivalis* group (11 of 21 pairs), the ANI values of all the species are high and uniform (Fig. 1b). These values suggest that only minor genomic differences exist within species and refute the hypothesis that global intra-species diversity could explain the variability in the number of contigs per draft genome. However, because small variations such as repeated genomic regions cannot be detected by the ANI method and since repeats have been declared to be the main cause of assembly gaps, we decided to evaluate the possible role of repeats in the fragmentation of draft genomes.

We determined the number and locations of each repeat type. Because Illumina is the industry standard and its MiSeq platform generates 2 x 250 nt paired reads, we set the low threshold to 500 bp and looked for the repeats. The histogram in Additional file 6: Figure S4 shows the mean number of repeats in each species (2 to 10 copies at 95% identity). Notably, although *B. fragilis* displays the greatest variation in number of contigs per draft, it has an intermediate number of repeats, and these have low copy numbers and occur a maximum of only six times. *P. gingivalis* can once again be distinguished as having the lowest total number of repeats but the highest number of copies for each repeat type, with a total of approximately 40 different repeats copied more than 10 times (Additional file 6: Figure S4).

**Fig. 2:**
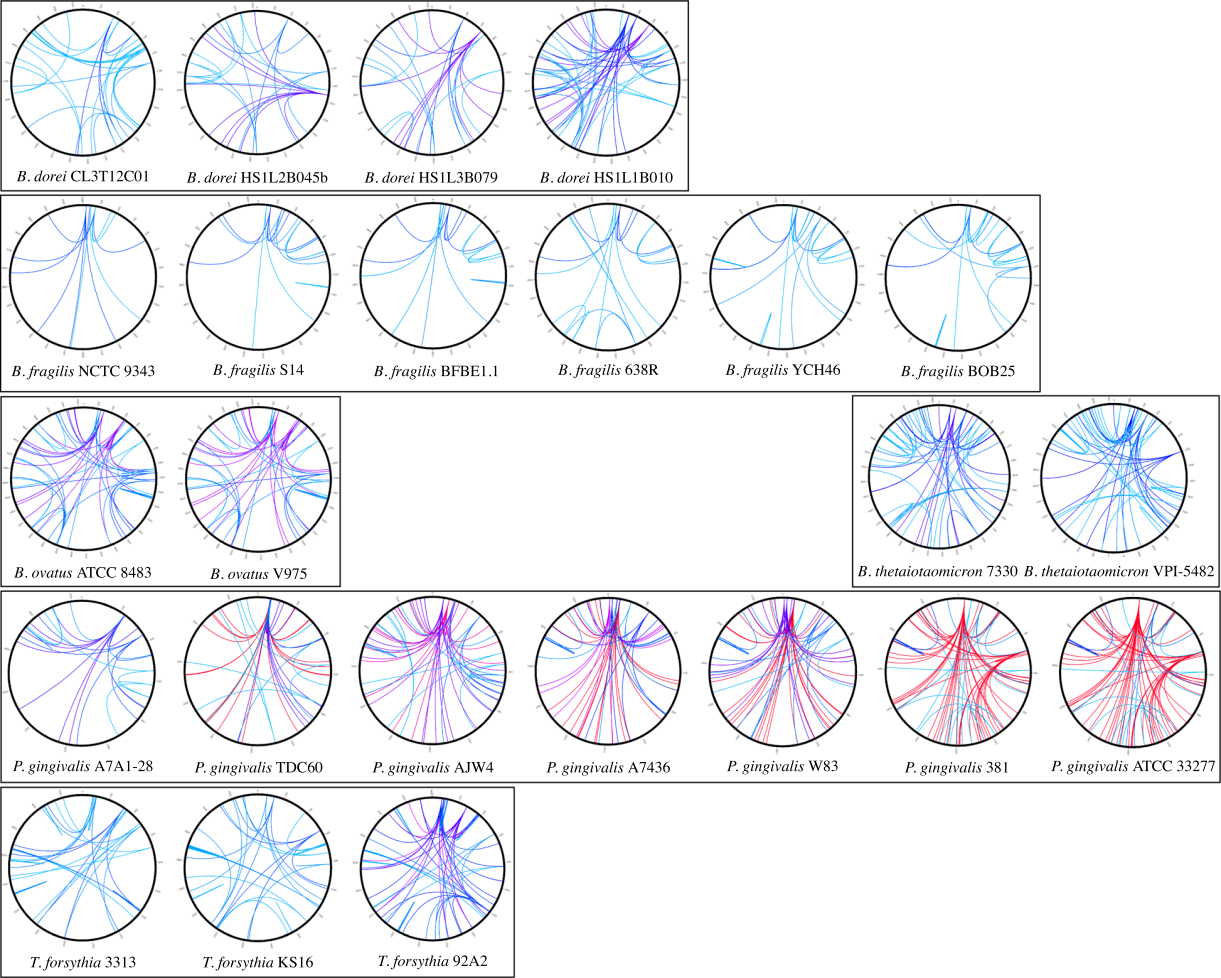
Genomic distribution of repeats (at least 3 copies) in each genome studied. Circos representations of each strain’s chromosome, with *oriC* positioned at the first nucleotide of the *dnaA* gene. For each repeat, its first occurrence in the genome is the starting point of each line that links it to all of the other positions. As the copy number increases, the line colours range from light blue to red. The total number of repeats can be visualized as the number of intersections of the circular chromosome. Strains of the same species are grouped together and arranged in ascending order of repeat counts

We used a Circos figure to visualize the genomic repeats with more than 3 copies in each complete genome. The plot illustrates the distribution of the repeated loci and their copy numbers in the form of a heat map, the color of which changes from blue to red as the count increases (Fig. 2). The plots show that all six available strains of *B. fragilis* possess few types of repeats and that repeats are not frequent. In contrast, all of the other *Bacteroides* species genomes (*B. dorei*, *B. ovatus*, and *B. thetaiotaomicron*) have more repeats that occur more often and display greater variability within the strains. Finally, we observed that the seven *P. gingivalis* strains had the highest genomic complexity and diversity with respect to repeat frequency.

**Fig. 3:**
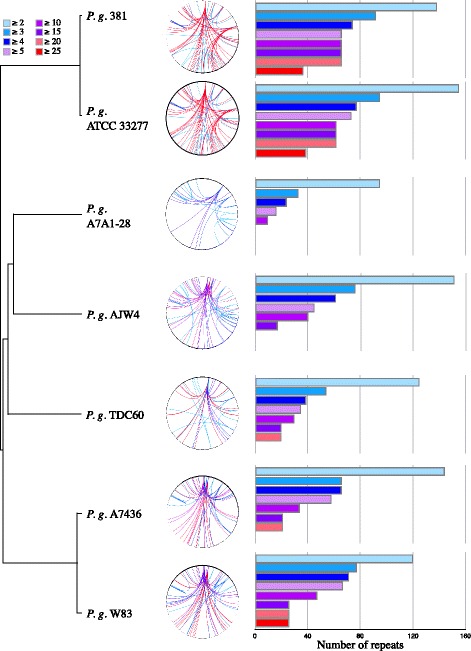
Genomic repeats in *Porphyromonas gingivalis* (*P. g.*) strains. From left to right, **s**train relatedness, genomic repeat distribution, and number of copies. The dendogram shows intra-species relatedness calculated with OrthoANI and clustered with UPGMA. The circular chromosome of each strain is presented using Circos, with *oriC* positioned at the top. For each repeat (at least 3 copies), its first occurrence in the genome is the starting point of the lines that link it to all other positions. As the copy number increases, the lines go from light blue to red. On the right, the number of repeats by copy number. Since all repeats have at least 2 copies, the total number of repeats corresponds to the light blue bar

The intra-species diversity of *P. gingivalis* and its large number of highly frequent repeats make this species an interesting model for analysis of the impact of genomic repeats on bacterial genome assembly, especially in Bacteroidetes. The *P. gingivalis* strains appear as three branches on the DNA-DNA distance tree. Two of these branches correspond to the previously mentioned closely related strains: ATCC 33277 with 381, and W83 with A7436 (Fig. 3). The ATCC 33277/381 branch contains the genomes with the most repeats, with some loci repeated more than 25 times, followed by the W83/A7634 branch. The remaining branch contains TDC60, AJW4, and A7A1-28, which display the lowest numbers and frequencies of repetition. With the exception of W83 (from Bonn, Germany) and TDC60 (from Tokyo, Japan), all of the cited strains were originally isolated in the USA. Despite this common origin, there is no apparent link between isolation populations and genomic repetition frequencies. To test whether the variation in genomic repeat counts affects assembly completion and since the real sequencing reads from the initial sequencing projects were not available, we produced *in silico* reads based on the complete published genome of the seven *P. gingivalis* strains and *in silico* simulated paired-end reads.

### Simulated sequencing reveals a correlation between contig and repeat counts

To generate the artificial reads, we used ART software. This set of tools mimics the real sequencing process and permits simulation of the data that are produced by massively parallel methods; the simulated data display each technology’s inherent empirical errors and profile qualities. For the seven complete *P. gingivalis* genomes, 11 assemblers were tested. The assembler spectrum was restricted to software that can treat Illumina reads but was otherwise chosen to be as wide as possible. Three of the assemblers are part of commercial software suites (Geneious, CLC Genomics Workbench, and CodonCode Aligner); the others are freely available for academic purposes. Since de Bruijn graphs are inescapable (they are used by A5-miseq, CLC Genomics Workbench, Geneious, Minia, MIRA, SOAPdenovo2, SPAdes, and Velvet), we made an effort to also include different assembly methods, including Overlap-Layout-Consensus (CodonCode Aligner), string graph (fermi), and greedy algorithm (PERGA).

**Fig. 4:**
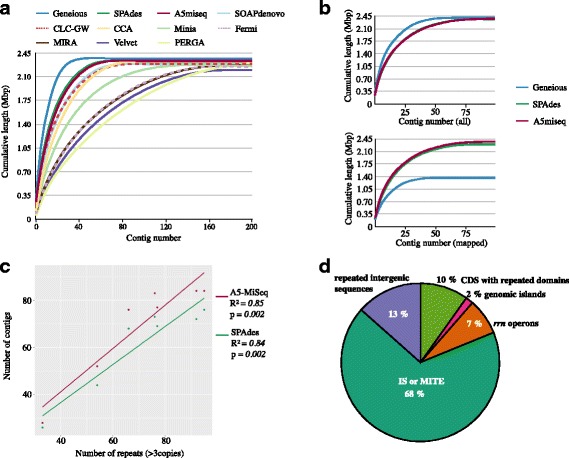
A *de novo* genome assembly of *Porphyromonas gingivalis* artificial reads. **a.** Eleven programs were used for *de novo* assembly of the seven strains in study. The main cumulative lengths were calculated, and plotted here against the contig index. **b.** The three assemblers that produced the highest N50 were plotted in the same manner as in a. (upper panel), then the assembly was mapped to the reference and only the mapped contigs were plotted (lower panel). **c.** The number of contigs (A5-miseq and SPAdes) was plotted against the amount of repeats (with at least 3 copies). **d.** Identification of gaps: after assembly with A5-miseq or SPAdes, genomic regions not covered by contigs were extracted. The gaps were classified into five categories: genomic islands, ribosomal RNA (*rrn*) operons, coding sequences (CDS) with repeated domains, intergenic sequences, and insertion sequences or miniature inverted-repeat transposable element (IS/MITEs)

We assessed each assembly’s fragmentation and compared it to that of the others using QUAST’s N50 parameter. For all *P. gingivalis* strains, Geneious produces the longest contigs and the fewest per assembly. SPAdes and A5miseq are just behind, with very similar performances. The other assemblers generate more fragmented assemblies, and some (Velvet and PERGA) do not even seem suitable for this bacterial group (Fig. 4a). Closer examination of the results of the three assemblers producing the highest N50 (Fig. 4b, upper panel) shows that the number of contigs obtained is below the median (90.5) of the published draft genomes assembled with real reads (Additional file 4: Figure S3b).

N50 can be misleading, however, because it provides no information on assembly accuracy; it can therefore give the impression that one tool is more algorithmically efficient than another even when its results do not agree with the biological sequence. Since the simulated reads were created from the complete NCBI reference genomes, mapping the resulting contigs against the genomes should be biologically consistent. The unmapped contigs would therefore be rejected as believed to be incorrect, resulting in the graph shown in the lower panel of Fig. 4b. The plot demonstrates that despite their high N50 values, Geneious contigs, especially the longer ones, are not consistent with the real sequence. The plot line will therefore fall short of the expected genome size, resulting in drafts that are not at all complete. In contrast, A5miseq and SPAdes yield similar plots since they accurately reproduce the biological sequences, despite the fact that their resulting contigs are shorter. Evaluating assembly completeness solely based on the N50 metric is a bad idea, and it is preferable to obtain a draft genome that is correct even if it is more fragmented. Consequently, A5miseq and SPAdes produce the best results for the *P. gingivalis* genomes (modeled with MiSeq reads), and we confirmed this to be true for the other Bacteroidia species as well (Additional file 7: Figure S5a).

A direct positive linear correlation can be observed in the number of *P. gingivalis* genomic repeats (at least three copied loci) and the number of biologically sound contigs produced by A5miseq or SPAdes from the *in silico* simulated reads (Fig. 4c). This correlation is also observed for the other complete Bacteroidia genomes studied here (Additional file 7: Figure S5b). In the case of *P. gingivalis*, it is true even when all genomic repeats, including duplications, are included (Additional file 7: Figure S5c).

We can classify the functions of the elements that are annotated in the breaking points of the A5miseq and SPAdes *P. gingivalis* assemblies. These gaps coincide with the repeated regions in the genome, and two thirds of them correspond to copied insertion sequences (IS) or miniature inverted-repeat transposable elements (MITEs) with a maximum length of 1.1 Kb. In order of their occurrence, the other four categories are: intergenic unannotated regions; repeats found in coding regions (such as gingipains) that have several paralogs and internal repeated motifs; *rrn* ribosomal RNA operons (present in four copies in *P. gingivalis*); and, the least frequent, genomic islands (Fig. 4d).

This study shows that to correctly assemble a genome from Illumina MiSeq reads of a few hundred nucleotides, it is helpful and even indispensable to have at least a good estimate of the quantity and frequency of genomic repeats. This knowledge permits the calculation of contig counts with little or no misassembly expected. It could also help in the choice of the best finishing strategy for the assembly. However, as seen here, this knowledge is often impossible to obtain in advance, since even among bacterial species the strains are very diverse.

### Choosing *Porphyromonas gingivalis* strains for resequencing and reassembly

Taking into account our initial results and observations, we chose to resequence three *P. gingivalis* strains (ATCC 33277, TDC60, and W83) using long-read technology from PacBio. We chose these strains because they are commercially available, allowing other researchers to reproduce our results, and because of their geographic diversity (they appear in North America, Europe, and Asia). Moreover, they were the first *P. gingivalis* strains to be sequenced and are considered reference strains. In addition, ATCC 33277 and W83 are frequently used to study mutants, in functional analysis, in adherence/invasion tests involving different types of human cells, and for other purposes.

For each strain, the mean coverage was approximately 30X (29.7 to 30.4), and the median corrected/trimmed read length obtained from canu was 6.3 Kb. The reads were assembled with canu, producing 18, 28, and 11 contigs for ATCC 33277, TDC60, and W83, respectively, and the finishing strategy described in the Methods section enabled assembly completion. All of the resulting complete assemblies differ from the initially published sequences. To validate the results, we performed PCR to confirm the organization of the large genomic multicopy regions. These included the *rrn* operons (Additional file 8: Figure S6a) and, for ATCC 33277, the duplication of the CTnPg1 genomic island and the orientation of the two copies (Additional file 8: Figure S6b-c). All of the RNA ribosomal operons from the ATCC 33277 and W83 strains were validated and found to correspond to the published structures. However, we corrected 3 of the 4 *rrn* in the TDC60 published genome, and this result confirms the observations made by Naito *et al.* in 2008 (see their Additional file 4: Figure S3) [100]. The most surprising result was the reorganization of 2 *rrn* loci in W83 compared to the other two strains (Additional file 8: Figure S6a). To determine whether this reorganization occurs only in this strain, 22 additional strains were subjected to PCR verification; we found that it is in fact restricted to W83 (Additional file 8: Figure S6d). The general arrangement of *rrn* operons in *P. gingivalis* chromosomes therefore seems remarkably stable despite the varied positions of these operons relative to the origin of replication (*ori*C) due to the high genomic mosaicism of the species. Homologous recombination at the *rrn* extremities seems extremely rare and probably accidental since of approximately 30 strains only W83 is affected.

### Differences from published genome sequences

With all three circular genomes confirmed, we proceeded to identify the ways in which these genomes differed from the previously published sequences for these strains. We used progressiveMauve, which identifies locally collinear blocks (LCBs) to compare the published genomes with our constructions. Insertions and deletions in the *de novo* assembly compared to the published sequence were noted (Additional file 9: Figure S7). The differences are reported in detail below.

In ATCC 33277, CTnPg1 is found to be completely duplicated, with both copies oriented in the same direction (Additional file 9: Figure S7). The other differences in this strain are three deletions and one insertion (Additional file 10: Figure S8a). Since the ANI values showed a significant similarity of the ATCC 33277 and 381 strains and the 381 strain also has two complete CTnPg1 copies, we compared our ATCC 33277 genome reconstruction with the 381 genome [101]. We observed two collinear genomes with a length difference of only 1 kb and approximately 100 SNPs. The dissimilarities, which are minor, occur mainly in repeats and in the CRISPR1 region. As the complete 381 genome was assembled from 454 reads using Velvet and Newbler, there could be assembly errors. By mapping the Illumina HiSeq reads of the recent 381 sequencing [102] to our *de novo* ATCC 33277 construction, we positioned 99.5% of the reads without any gaps, suggesting that these American strains could be variants of the same strain.

TDC60 is the most changed, since, as discussed, 3 of the 4 *rrn* were incorrectly assembled during the initial construction. This means that in our new assembly, large sections of the genome are translocated (LCB2 and LCB5) and inverted (LCB3) (Additional file 9: Figure S7). The other differences are one insertion into a BrickBuilt 7 MITE [103] and four deletions (Additional file 10: Figure S8b).

Finally, the new W83 strain has a central inversion (LCB2) (Additional file 9: Figure S7). The new sequence consists only of a 523-bp insertion, which corresponds to eight additional direct repeats and the same number of new spacers in the CRISPR2 region (Additional file 10: Figure S8c). The genome length differences and numbers of SNPs in the three strains are shown in detail in Additional file 9: Figure S7.

### Annotation and manual biocuration of three selected *P. gingivalis* strains

We automatically *de novo* annotated the three *Porphyromonas gingivalis* strains using three pipelines, after which they were manually biocurated. This allowed us to standardize the structural (syntactic) annotation, which consists mostly of CDS start/stop codon positions, and the functional annotation, using a single ontology for gene names and functional descriptions. The number and nature of all 12 rRNA genes (in four operons), 53 tRNA genes, and 1 tmRNA gene are identical to those in the published genomes, although the positions of some relative to *oriC* vary due to the reassembly. Seven of the riboswitches (5 cobalamin and 2 thiamine pyrophosphate riboswitches) were already annotated in all of the strains, and we added 1 S-adenosyl methionine (SAM)-II long-loop riboswitch per strain. It is noteworthy that all of these riboswitches were described by Hovik *et al*. [104]. For the small non-coding RNA (ncRNA), only *rnpB* (bacterial RNAse P) is present in the previous annotation. We positioned and annotated additional ncRNAs: one each *ctRNA* (antisense RNA), Bacteroidales-1 RNA, and bacterial signal recognition particle (SRP RNA) for each strain. We also added various group II catalytic introns: 5 in ATCC 33277; 4 in TDC60; and 3 in W83. For all riboswitches and ncRNA, the synteny is conserved. Two group II catalytic introns are present in *haeR* and in *punA*. The variation in numbers in this group is explained by the paralogy in *traE* (a gene containing this ncRNA is present once in W83 and twice in ATCC 33277 and TDC60) and by an additional ncRNA in the ATCC 33277 *traG* gene that is absent from the other two strains.

For ATCC 33277, TDC60, and W83, we added 213, 199, and 188 peptide signals and 23, 19, and 21 mobile elements (genomic islands, transposons, and conjugative transposons), respectively, to the original annotations. We further completed the annotation of intergenic sequences with repeated elements: BrickBuilt/MITEs (complete or partial), dispersed genomic repeats (> 500 bp with at least 95% identity), and CRISPR regions. We did not annotate any short repeats (3 to 31 bp) or sequence-tagged sites.

Finally, the longest and probably the most important biocuration work involved the CDS and pseudogenes, which we were able to dramatically improve. To facilitate traceability in publications and databases, we kept the initial locus_tags when the DNA sequence was unchanged or very similar (only having a new start and/or stop position). When the differences were larger (e.g., changes in the coding strand, ORF fusion, or a new CDS), we created new locus_tags marked *PGN_n*, *PGTDC60_n*, and *PG_n* for ATCC 33277, TDC60, and W83, respectively. In the previous versions, the pseudogenes had coding sequences and gene annotations, with pseudogene status annotated in the gene qualifier “note.” These could be classified into the categories “frameshifted,” “incomplete,” and “internal stop.” Following the NCBI Prokaryotic Genome Annotation guidelines (https://www.ncbi.nlm.nih.gov/genbank/genomesubmit_annotation/), we annotated the pseudogenes with the gene feature only (no CDS) and added an asterisk to the gene name field so that pseudogenes could be easily distinguished from normal coding regions. The gene_desc qualifier field describes the function of the pseudogenized gene, and the reason for the pseudogenization (“fragment” or “frameshift”) is listed in the note qualifier. Where they are relevant, gene fusions and coding strand changes are also mentioned in the “note” field.

For the three strains, our revised annotation contains fewer CDS and pseudogenes than are found in the original annotations (Table 3). These differences are essentially due to over-annotation of the original genomes and to genome-to-genome annotation propagation caused by the difficulty of distinguishing between small coding ORFs and Evil Little Fellows or ELFs [105, 106]). These are regions that can accidentally produce ORFs that are not biologically verified. To avoid erroneous elimination of real small ORFs, we identified ORFs as ELFs only if one of the following conditions applied: if the ORFs were annotated in intergenic repeats such as MITEs, which by definition do not code [107]; if the ORFs were not conserved in all 30 available *P. gingivalis* complete and draft genomes but the DNA sequences and synteny were conserved; and if the ORFs were small and possessed an AMIGA status that was “wrong” [108]. We systematically predicted the cellular localizations and/or the conserved functional domains of the small conserved ORFs. This sometimes enabled us to improve the annotation of the hypothetical functions of small CDS into “lipoprotein,” “inner membrane,” “periplasmic,” “outer membrane,” or “secreted.” Finally, the use of manual biocuration permitted us to correctly identify some coding sequences as pseudogenes and vice versa, to fuse two initial CDS/pseudogenes into one, and to divide a CDS/pseudogene to create two or more. In rare cases (*n* = 5), our re-annotation produced a coding strand change for the ORF involved (Additional file 11: Table S4).

- Comparison of the original annotations and our comprehensive syntactic re-annotations

**Table 3:** Summary of CDS and pseudogenes (in parentheses) in the original and curated annotations

	Original annotation	Curated annotation	Common to both	Curated start/stop positions	Eliminated ELFs
ATCC 33277	2051 (96)	1856 (60)	1722 (22)	155 (8)	165 (46)
TDC60	2031 (89)	1794 (34)	1742 (13)	164 (8)	172 (64)
W83	2027 (112)	1801 (77)	1703 (45)	163 (14)	165 (53)

To compare the original and curated annotations, we began by summarizing the common CDS and genes in the two versions as well as the number of curated start and stop positions and eliminated ELFs (Table 3). Additionally, for ATCC 33277, we added 55 new coding sequences; these usually corresponded to the re-establishment of a complete CTnPg1 copy or the reconstruction of complete ISPg CDS copies. For the TDC60 strain, we added 2 new pseudogenes (ISPg) and 6 new CDS (coding for 2 ISPg, an NAP, a peptidase, a rhodanase, and a PF07877 domain-containing protein). Finally, in the W83 strain, we *de novo* annotated 6 pseudogenes (5 ISpg and a PF07877 domain-containing protein) and 12 CDS (these encode 7 ISPg, 2 integrases, an NAP, a transcriptional regulator HxlR, and a virulence-related RhuM protein).

To compare the functional annotations in the two versions, we grouped the common features into five categories: nucleic acids and protein metabolism (replication, transcription, translation, histones, proteases, etc.); metabolism and transport of ions and other macromolecules (transporters, porines, ion channels, ferritin, kinases, hydrolases, etc.); mobile elements (conjugation, competence, phages, etc.); proteins without assigned biological functions but with a predicted subcellular localization or possessing an identified functional domain (lipoproteins, inner/outer membrane, or proteins which contain GLPGLI or zinc finger domains, etc.); and proteins that are conserved in all available *P. gingivalis* genomes but to which we could not assign a function (DUFs, FIGs, and some COGs, all marked as hypothetical).

**Fig. 5:**
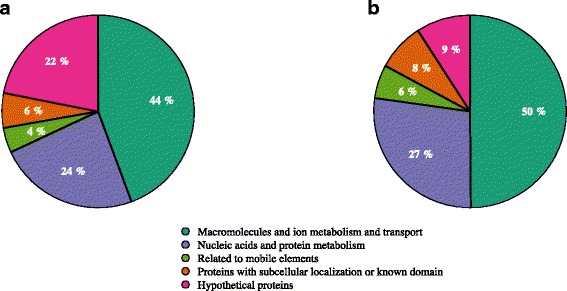
Functional comparison of the common coding sequences in two *Porphyromonas gingivalis* annotations. Comparison of **a.** an annotation available at the NCBI, and **b.** this study’s manually biocurated annotation. For both, the common CDS were classified into five categories. Both pie charts reflect mean values

Our biocuration and re-annotation work on the coding sequences and pseudogenes shared by the two versions resulted mainly in the de-anonymization of hypothetical CDS/proteins of unknown function (Fig. 5). These constituted approximately 22% of the original annotation and now represent only approximately 9%. The newly described functions were placed into four other categories as follows: 6% were added to macromolecule/ion transport and metabolism, an additional 3% were placed in nucleic acids/protein metabolism, mobile elements increased by 2%, and 2% were added to the list of CDS with a predicted conserved domain and/or subcellular localization. Finally, we noted that for the new pseudogenes and coding sequences as well as all of the other changes (features, fusions, separations, etc.), the new annotations are particularly enriched in mobile elements (Additional file 11: Table S4).

### Pangenome analysis

**Fig. 6:**
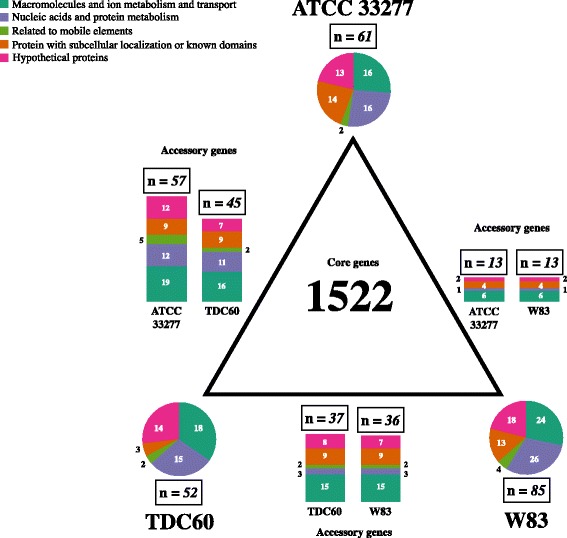
Pangenome overview of ATCC 33277, TDC60, and W83 strains, focusing on accessory and unique genomes. The central triangle represents the core genome, which has at least 1522 genes (see text for details). Each corner is a *Porphyromonas gingivalis* (*P. g.*) strain, with a pie chart showing the unique genome’s distribution of functions, with total and absolute counts shown. On each triangle side, stacked histograms show the accessory genome of the strains in the adjacent vertices. Total and absolute counts are shown, and the differences between strain numbers are due to paralogy

All gene annotations (both true CDS and pseudogenes) from the three strains were classified into the five previously cited categories (Fig. 6). As shown in Additional file 12: Figure S9, with the exception of genes related to mobile elements, the absolute number of genes in each category is uniform. These transposases, integrases, phages, and conjugation genes are overrepresented in ATCC 33277, and underrepresented in TDC60. Even after manual biocuration, 16.8% of all genes remain poorly characterized; 7.7% have only subcellular localization predictions or functional domains, and 9.1% of the proteins conserved in *P. gingivalis* still have unknown functions.

- The *P. gingivalis* ATCC 33277, TDC60 and W83 pangenome can be divided into four categories

The first class is genes that are present in at least 2 copies in all of the studied strains; these represent 10.1% of the total number of genes in ATCC 33277 and 7.5% of the total genes in the other two strains. Some, such as DNA helicase, transcriptional regulators of the helix-turn-helix (HTH) 17 family, and nucleoid-associated proteins (NAP), are involved in nucleic acid metabolism. The others are associated with horizontal gene transfer and include integrases, tetracycline-resistance elements, *tra* genes of conjugative transposons, and transposases encoded by several types of insertion sequences (Additional file 13: Figure S10a). Transposases represent more than a third of the multicopy genes and are highly pseudogenized, particularly in W83 (Additional file 13: Figure S10b). Their distribution in the strains is variable; ISPg8 (previously known as ISPg1) occurs the most frequently, followed by IS195 (formerly ISPg3). It is notable that W83 is the only strain rich in ISPg4. In ATCC 33277, we annotated a pseudogenized ISPg5 despite this gene’s being described as absent by Califano *et al.* [109] (Additional file 13: Figure S10c).

The next category is the “dispensable/accessory genome,” which consists of genes that are present in only two of the three strains (70 genes in ATCC 33277, 82 in TDC60, and 49 in W83). Genes with unknown function are more abundant, with 40% (38.6 to 44.9%) poorly characterized genes in the accessory genome vs. 17% in the core (Fig. 5). Many of these genes are in chromosomal regions that have been annotated as genomic islands.

The third gene class (“strain-specific or unique genes”) consists of genes that are present in only one of the three strains. This class includes 3.3% of the genes in ATCC 33277, 2.9% of the genes in TDC60, and 4.7% of the genes in W83. Within this class, genes related to mobile elements *per se* are rare (< 5%), and genes of unknown function represent approximately 45% in ATCC 33277 and approximately 35% in TDC60 and W83. The singularity of these “unique” genes is relative since BLAST analysis of *Porphyromonas* genomes yields homologs in at least one other genome. This is often the case for ATCC 33277 genes in the 381 or HG66 strains and for W83 genes in the A7A1-28, A7536, and AJW4 strains. Some of the unique genes present in the three strains have hits in *Tannerella forsythia* genomes. Many of the unique genes are clustered in the genome.

The final gene class can be referred to as the “core” genome because it contains the genes that are present in all three strains. This class includes more than 80% of all genes (82.8% in ATCC 33277, 85.0% in TDC60, and 84.7% in W83) and can be further subdivided into a constant core, a variable core, a paralogy core, and a pseudogenized core (Additional file 14: Figure S11a). Due to paralogy, the total number of genes in the core genome varies, but it starts at 1522 genes.

The first subgroup of genes in the core genome is the “core genome *sensus stricto*” or the “constant core genome,” which contains genes with only one ortholog in each strain and more than 97% nucleotide identity. The constant core represents the majority of the core genes (*n* = 1424, 93% of the core); we classified these genes into the functional categories described above (Additional file 14: Figure S11b). There are only four genes related to horizontal gene transfer, and all four (*pir*, *yhaI*, *vrgG,* and *p12*) correspond to phage-related proteins. Since no phage infecting *P. gingivalis* has been described [110], it would be interesting to study the significance of this finding. We also observed four pseudogenes that are present in the three strains: *COG4335,* a hypothetical protein with a conserved domain associated with DNA alkylation activity [111]; *pspB,* which encodes alpha-ribazole-5’-phosphate phosphatase, an enzyme involved in coenzyme B12 biosynthesis; *pyrD,* coding for dihydroorotate dehydrogenase, which is involved in the *de novo* biosynthesis of pyrimidine; and finally *tetR,* which encodes a transcriptional regulator.

The second subgroup is the “variable core”, consisting of genes that also only have one ortholog but display less than 97% nucleotide identity (ranging from 96.9 to 66.3%). This subgroup represents only 3% of the core or 47 variable genes (Additional file 14: Figure S11c).

The third subgroup is the “core with paralogy”; it contains genes with only one ortholog in a strain but with duplications in at least one of the strains. This subgroup represents only 2.5% of the core genes (*n* = 36). The genes in this subgroup are related to DNA transcription, modification and repair (*alkD, betI, btr,* and *ytxK*), and transport (*irtA, ndvA,* and *ydfJ*). There are also four genes of known function (*ctpA* protease, *epsJ* glycosyltranferase, *era* GTPase, and the *rhuM* virulence protein) and four hypothetical proteins (Additional file 14: Figure S11d).

The fourth and final subgroup is the “core with pseudogenization”, which includes genes with only one ortholog in each strain but also at least one pseudogene in another strain. This group contains 36 genes, represents 2.5% of the core genes, and includes genes with unknown function as well as genes involved in adaptation and/or pathogenicity, for example, genes involved in transport, nucleic acid metabolism and cellular appendages (Additional file 15: Table S5).

## Discussion

Bacteroidetes dominate human intestinal bacterial communities [112], but these taxa are also associated with other animals and found in soil and aquatic environments. This suggests that they play an important role in biogeochemical processes [8]. *In silico* modeling allows us switch from describing microbial communities to actually predicting genotype-phenotype relationships and microbe-microbe and microbe-host interactions, but it requires genomic reference sequences that are both accurate and exhaustive [113, 114]. The existing databases contain thousands of bacterial genomes, but their quality is poor since nearly 90% of all available bacterial genomes are merely drafts [40, 115]. Furthermore, the biodiversity of the databases is biased because the data were primarily obtained from pathogenic or biotechnologically/economically interesting bacteria [116–118]. As an example, Bacteroidetes, despite their abundance among human microbiota, represent a minority of the species in genome databases, with some overrepresented species such as *Bacteroides fragilis*. Moreover, even when a reference genome is available for a bacterial species, this is not sufficient to permit evaluation of the true biological diversity of its strains [119]. Of even more concern, the number of drafts is actually underestimated since some genomes marked “complete” or “finished” are fragmented and have not been fully annotated. For example, we observed (Additional file 16: Table S1) that two of nine *Porphyromonas gingivalis* genomes described as complete were artificially circularized: HG66 [120] has an assembly gap of unknown length (represented by 100 N), and JCVI SC001 [121] contains 282 assembly gaps, abnormalities also noticed by Chen *et al.* [122].

Draft genomes are made up of a set of contigs of varying size and unknown order, orientation, and quality. They may contain sequencing and assembly errors as well as absent, fragmented, and/or frameshifted genes and other artifacts [44, 117, 123, 124], and these irregularities can also occur in closed genomes, as demonstrated by this study. Worse, without a complete genome it is difficult to identify possible contaminant sequences; in some draft genomes, even human genome sequences can be observed [125–127]. Nevertheless, most current sequencing project teams settle for drafts, limiting our knowledge of the genomic structural organizations crucial for understanding bacterial evolution and adaptability [128]. Comparative genomics of two incomplete genomes can yield a description of most of the protein-coding genes, but only complete genomic information will allow us to explore the frequency and localization of repeated sequences, paralogy, synteny, and genomic rearrangements. Incomplete data also hamper the interpretation of ecological models and evolutionary reconstructions [116, 129].

Draft quality assessment is difficult, especially in the absence of a reference genome [130]. It is usually evaluated using the N50 parameter [117, 131], with a higher value indicating better quality [132]. However, our study shows that N50 is inadequate for judging an assembly’s biological value because it favors long contigs even when they are misassembled. Dozens of assembly software packages exist, and comparative studies have shown that their effectiveness depends on factors such as genome size, coverage, sequencing technology, and the presence of atypical DNA (transposons, plasmids, and phages) [124, 133–136]. Our analysis shows that this biological variability also influences assembly.

Using an *in silico* sequencing strategy to evaluate *de novo* assemblies obtained using different software packages, we compared the number of genomic repetitions (*rrn* operons, insertion sequences, adhesins, proteases, etc.) and the number of obtained contigs. Despite the fact that repetitions are often cited as being responsible for assembly breaks [102, 123, 137, 138], as far as we know our study is the first to demonstrate a strong correlation between the number of repeats and the number of contigs. These repeats “break” the assembly and are quite often present as individual contigs or at the ends of contigs in draft genomes. However, repeated elements are important for genome plasticity (rearrangements, duplications, inversions), and even considering that their genome coverage is estimated at 6.9% for prokaryotes [139], their apparent percentages vary widely even within the same species (4.8 to 6.7% for *P. gingivalis*, 1.5 to 2.4% for *B. fragilis*), and they are impossible to evaluate *ab initio*. Genomic repeats are frequently transposases in insertion sequences [140, 141], and they represent two-thirds of the breakpoints in *P. gingivalis* assemblies. Sequencing technologies that generate reads shorter than the repeat length are not suitable for resolving these assembly problems [123, 142, 143].

All of the molecular studies of the *P. gingivalis* genome published in the last 10 years have shown that the genomes of *P. gingivalis* strains are highly variable [144–148]. Our average nucleotide identity (ANI) calculations show, however, that as described by other authors [144, 149], *P. gingivalis* is a single species with a genomic heterogeneity indicating a non-clonal population. Furthermore, this variability is common to opportunistic pathogens that are responsible for chronic colonization and infection [150, 151]. *P. gingivalis* is therefore an interesting model for exploring the relationship between strain genomic diversity and potential differences in pathogenicity and virulence. The potential of DNA recombination in this diversity is facilitated by the natural competence of *P. gingivalis* [148]. This suggests that the species represents a panmictic population [146] with high genomic mosaicity, as confirmed in our study. The nutritional use of exogenous DNA as a carbon and energy source certainly facilitates recombination [148].

In this study, we chose to resequence three *P. gingivalis* reference strains from international collections. We demonstrated that even modest long-read coverage (~30X) combined with biocurated assembly and some PCR contiguity validation could correct these highly plastic genome assemblies. This result is supported by previous studies of other bacterial species [38, 43, 152, 153].

Our work confirms the genomic diversity and plasticity of *P. gingivalis* but also shows that the species includes clonal subpopulations of closely related strains. This is especially the case for ATCC 33277 and 381 and to a lesser extent for the W83 and A7436 strains. We confirmed the relatedness of ATCC 33277 and 381 mainly through the reconstruction and reorientation of two full copies of the CTnPg1 conjugative transposon in the ATCC 33277 genome. These two strains are either cited as identical strains [102, 122, 154] or as variants (ATCC 33277 being a natural streptomycin-resistant mutant [155, 156]). Our study shows the importance of submitting the sequencing reads to databases such as the NCBI SRA sequence read archive so that they can be reused for further analysis. This allows the scientific community to complete the study of genomes, adding value to the work of the initial researchers. Unfortunately, however, many reads are not publicly available, since it is not mandatory to upload them (even if it is highly recommended).

As previously stated, the main reason for sequencing several strains from a single species is comparison of their genomes with the goal of explaining phenotypic differences and understanding the evolutionary history and adaptation of the species. To do this, we compared the three resequenced *P. gingivalis* strains after performing a thorough manually biocurated annotation. Similar to Guo *et al.* [157], our biocuration strategy involves homogenizing transcription initiation sites, rigorously identifying frameshifts, internal stop codons, and intergenic low complexity repeats, and eliminating false CDS predictions; finally, if coding sequences only have hypothetical functions, we assign functions or predict the subcellular localizations of their gene products. Although manual biocuration is time-consuming and labor-intensive, it is essential for proper comparison [115, 158] and to avoid the false positives and negatives propagated by automatic annotation pipelines [159–161]. After this step, the mean genetic density was 85.6%, closer to the mean value of approximately 85% that has been described for prokaryotic genomes [162] than to the value of 87.5% obtained through automatic annotation. CDS boundaries were analyzed via comparative ortholog analysis, and we made corrections in the corresponding genes for the three strains. Biocuration resulted in changes in start codon use, with AUG (Met) used in 97.4% of cases for the three strains (an increase from 85%). UUG (Leu) is the next alternative codon at 2.5% (initially 9.5%), and GUG (Val) is used in 1.1% of cases (vs. the original 5.5%).

Our consistent annotation yields an accurate description of the pangenome. However, the multiple-copy genes deserve a study of their own to analyze their content and to determine how frequency differences are related to phenotypes. We identified 1522 constant core genes, equivalent to 82.5-85.0% of all protein-coding genes. This is close to the 83% (1488) estimation of Dashper *et al.* [102], the 1490 described as common by Naito *et al.* [100], and the 1476 core genes identified by Brunner *et al.* [163] but very different from the 55% (*n* = 1037) estimated by Chen *et al.* [122]. Why are these values so different? There are at least four possible reasons for this. First, the number of strains studied varies widely (23, 2, 8, and 19 in the studies of Dashper, Naito, Brunner, and Chen, respectively). This could explain the smaller differences (0.7% to 3.8%) between our study and the first three cited studies, but not the difference of greater than 30% between our results and those of Chen *et al.* Next, the differences may result from the nature of the studied genomes. Unlike our study, which was based only on complete and verified genomes, the Chen and Dashper groups based their results mostly on draft genomes. As previously mentioned, incompleteness can falsely indicate that some coding regions are absent and can artificially enrich unique strain-specific coding sequences. This may have been the case in the Chen analysis and would explain the small number of core genes that were detected. Another possible explanation for the differences is the way in which we calculated the core genes. Chen compares all of the automatically annotated CDS, without any evident biocuration, whereas Dashper used a more biocurated annotation. Finally, in the description of the core genome, we included variable genes as well as pseudogenized or duplicated genes that are functional orthologs. These have all evolved differently, and due to low nucleotide identity in reciprocal best hits BLAST analysis were previously wrongly described as unique or strain-specific genes. For example, this is the case for the major fimbrillin gene *fimA,* which displays 66.3% nucleotide identity and 60.1% protein identity in the three strains.

Comparison of the essential genes described by Klein *et al.* (*n* = 463) [164] and Hutcherson *et al.* (*n* = 281) [165] to those in our classification shows that the vast majority of essential genes are present in the constant core genome (96% and 98.5%, respectively). Six of the genes described as essential by Klein but not by Hutcherson were eliminated by our biocuration due to the presence of MITEs or because the observations showed a conserved nucleic acid sequence but not an ORF. The genes eliminated were short and close to the 5'- or 3'-UTRs of coding genes (10 to 250 nt). This polar effect could be caused by the transposon mutagenesis used in both of the abovementioned studies, with a change in one gene perturbing the transcription of adjacent genes [166]. Two essential genes (*PGN_0919* and *PGN_1215*) described by Klein but not by Hutcherson are specific to the ATCC 33277 strain in our study, but their presence can be observed in *P. gingivalis* strains 381 and HG66 and in other bacterial genera in various phyla, including *Parabacteroides* and *Prevotella* in Bacteroidetes, *Bacillus* in Firmicutes, and *Rhizobium* and *Vibrio* in Proteobacteria. This might indicate an exogenous origin, which would be consistent with their locations in or near genomic islands. This observation confirms the importance of the biological characterization of proteins with unknown functions and shows that such effort is vital for functional genomic interpretation and identification of proteins of interest [167]. The presence of homologs of strain-specific genes in other strains or species challenges the existence of ORFans, unique or orphan open reading frames [168]. Our re-assembly and re-annotation work produced two noteworthy and highly correlated improvements: fewer genes of unknown function and fewer ORFans in all three strains. The number of unique genes in ATCC 33277, TDC60, and W83 was initially 461, 415 and 382, respectively [100, 169]. This represents 17-22% of all protein-coding genes and was reduced to approximately 3% in our study, a value that is closer to the estimate of 6-7% unique genes obtained using experimental microarrays [145, 154]. Of the 382 unique genes described for TDC60, more than two-thirds were described as hypothetical [169]. Naito *et al.* noted that more than 60% of the unique coding sequences had similar sequences in other strains that did not fulfill their study’s criteria (cut-off of > 60% alignment length and > 90% identity) [100]; these could be allelic isoforms of the same gene [170]. Since unique genes might be involved in adaptive responses to environmental changes, it is important to obtain accurate annotations. Our analysis of the three strains shows that many loci initially described as unique correspond to regions of synteny that display nucleotide sequence homology but coding loss in one or many strains. In some of these regions, biocurated annotation identifies pseudogenes or non-coding repeated interspersed elements such as MITEs. In others, it is more likely that the coding loss involves a conserved region that expresses a promoter, a terminator, or even a common ncRNA but not a CDS. We only included the regions that conserved their coding capacities in all 29 *P. gingivalis* strains studied (Additional file 16: Table S1, Additional file 5: Table S2, Additional file 17: Table S3) and that did not have new overlapping annotations.

It is interesting to note that the genes in the variant core that have been reported in the literature are mostly associated with virulence factors coding fimbriae/pili [171], hemagglutinins, surface proteins and transporters [149, 163], and *cas* genes, completing and reconfirming the observations of Igboin *et al.* [145]. However, although we have predicted their subcellular localizations, the products of some of these genes are still of unknown function. Perhaps, as in the case of many membrane proteins, these loci encode proteins that have new functions, are involved in *P. gingivalis* environmental interactions or confer differential pathogenicity or virulence [170, 172], since virulence differences are probably due to differing external envelope components and adhesion capacities [173].

In ATCC 33277, our reannotation positioned 23 mobile elements (MEs) in 14 regions (2 of these were separated by less than 2 kb); this is in accordance with the 13 atypical regions initially described [100]. In contrast, in W83 we only annotated 12 MEs in 11 regions, 10 fewer than in the initial annotation [174]. In TDC60, we again annotated 12 MEs in 11 regions, thus actually enriching the previous annotation, which only included 4 [169].

As a final remark, reference-guided genome assembly should be avoided for a bacterial phylum such as Bacteroidetes (and especially its *Porphyromonas* genus) that has high genomic plasticity and frequent repeats. The method is unreliable and is a source of errors due to the numerous genomic rearrangements. Long-read *de novo* assembly is clearly the strategy of choice for obtaining complete and accurate finished genomes. Even though the sequencing and assembly of complete genomes is expensive, time-consuming, and requires manual biocuration, it should be the goal for high-quality sequencing projects [175, 176]. In a bacterial species, having several consistently sequenced, assembled, annotated, and biocurated genomes is essential for comparative genomic studies, permitting the analysis of genomic plasticity and evolutionary mechanisms [177].

## Conclusions

Current sequencing capacity is yielding more and more bacterial genomes at a continuously lower price, yet the vast majority of these projects release draft genomes. In agreement with previous observations, in this study we showed that assembly breaks are caused by genomic repeats that are equal to or longer in length than the sequencing reads. Nevertheless, these repeats encompass a vast variety of elements that are essential to genome organization, stability, and function. They are therefore inherent parts of genomes, yet most are not shown in draft genomes. When possible and according to the biological question to be answered, a complete finished genome should be the preferred aim of sequencing projects, and long-read sequencing makes this feasible. We have demonstrated that this technology allows the verification of bacterial genomes that have been sequenced, assembled and circularized using massively parallel sequencing technologies. This method will also detect misassembly errors that are often associated with erroneous combinations of ribosomal operons or very long genomic islands. Finally, we have validated the importance of biocurating automatic annotations and have shown that a strategy based on comparative genomics is very powerful for improving both structural and functional annotations.

## Additional files

Additional file 1: Table S1. Complete genomes not included in this study. When available, the species, strain, sequencing technology, assembler, coverage, Pubmed ID, release date, exclusion reason, and FTP link are presented for each genome. When no publication was found, genomes were marked as “unpublished,” and the sequencing organism was identified. Key: BCoM, Baylor College of Medicine; BU, Bielefeld University; DOE-JGI, United States Department of Energy-Joint Genome Institute; FI, The Forsyth Institute; HMP, Human Microbiome Project; JCVI, J. Craig Venter Institute; LIAEPB, Leibniz Institute for Agricultural Engineering Potsdam-Bornim; SI, Sanger Institute; and UoB, University of Bern. (PDF 43 kb)

Additional file 2: Figure S1. NCBI genome database distribution of the main bacterial phyla associated with humans. **a.** Pie chart featuring the genomes present in the database, by phylum. **b.** Stacked bar chart of the incidence of the genomes belonging to the four main phyla associated with humans. Absolute counts are presented by phylum and classified as either finished/complete (having at least one chromosome and/or plasmid), or draft/incomplete (having multiple contigs or scaffolds). (PDF 61 kb)

Additional file 3: Figure S2. Bacteroidetes genomes by class. **a.** On the left, a phylogenetic tree based on the 16S rRNA genes of complete genomes, grouped by class. A stacked bar chart then shows the number of genomes belonging to each Bacteroidetes class. The absolute genome counts are given, and classified as being either finished/complete or draft/incomplete (having multiple contigs or scaffolds). Pie charts on the right indicate the isolation sources for each genome: environmental (soil, fresh or marine water, and plants), animal (insects, molluscs, fish, birds, and mammals), or human (different body sites and health conditions). **b.** Stacked bar chart of Bacteroidia genomes grouped by status (complete or draft), presented by their publication year. (PDF 95 kb)

Additional file 4: Figure S3. Bacteroidia draft genomes binned by genus and by species**. a.** Box plot of draft/incomplete Bacteroidia genomes grouped by genus. With the exception of *Tannerella* which has complete genomes, any genus with less than 10 draft genomes was classified as “other.” The number of assemblies is presented above the plot, and the median is shown for each box. If a genus has drafts with more than 500 contigs/scaffolds, it is marked with ◉: Bacteroides (*n* = 17, 557 to 4357 contigs); Parabacteroides (*n* = 2, 1471 and 1920 contigs); and Prevotella (*n* = 3, 553 to 3171 contigs). **b.** Box plot of draft/incomplete Bacteroidia genomes for which at least two complete genomes exist, grouped by species, as per **a**. The drafts which have over 500 contigs/scaffolds are *Bacteroides fragilis* (*n* = 7, 557 to 2566 contigs), *B. ovatus* (*n* = 1, 556 contigs), and *B. thetaiotaomicron* (*n* = 2, 1730 and 2372 contigs). (PDF 34 kb)

Additional file 5: Table S2. The 166 draft genomes of the six Bacteroidia species studied here. Species, strain, sequencing technology, assembler, number of contigs, Pubmed ID, release date, and FTP links are presented. When no publication was found, genomes were marked as “unpublished” and the sequencing organism was identified. Key: *, no sequencing centre could be identified; BCoM, Baylor College of Medicine; BI, Broad Institute; DOE-JGI, United States Department of Energy-Joint Genome Institute; FMBA, Federal Medical-Biological Agency, Russia; HMP, Human Microbiome Project; IGS, Institute for Genome Science, University of Maryland; JCVI, J. Craig Venter Institute; TUD, Technical University of Denmark; UoS, University of Sheffield; and WU, Washington University. (PDF 1709 kb)

Additional file 6: Figure S4. Genomic repeats by species. Genomic repeats were identified for each genome, and the cumulative mean copy numbers and their standard deviations are presented. The light blue bar indicates the total number of repeats (at least 2 copies). (PDF 649 kb)

Additional file 7: Figure S5. *De novo* assembly of artificial reads of the studied Bacteroidia genomes**. a.** QUAST graph (cumulative length versus config index) for each assembly of each strain. The left column shows all contigs (> 1 Kbp), while the right shows only the contigs that mapped to its reference. The dotted line represents the reference genome size. **b.** For all 24 genomes, the contig counts from A5-miseq and SPAdes were plotted against the repeat counts (with at least 3 copies). **c.** As b, but showing all seven *P. gingivalis* strains. (PDF 133 kb)

Additional file 8: Figure S6. PCR validation of 3 *P. gingivalis* strain constructions. **a.** Agarose gel electrophoresis (0.8% in 1X TBE buffer, stained with 1X GelRed) of PCR products for rrn operons. Primers used and verified strains are indicated by lane, and the primer names were simplified (“rrn” is not mentioned). DNA molecular-weight size markers were used in the first and last lanes. The first band is 9 Kb, and the second is 4 Kb. **b.** Schematic representation (not to scale) of both copies (“a” and “b”) of CTnPg1 from *P. gingivalis* ATCC 33277. The *de novo* assembly identified two complete copies with the same orientation. The published ATCC 33277 strain had two copies, but the second was partial and inverted when compared to the first. Size and orientation are presented for clarity, and the dotted line indicates the absent CTnPg1-b region in the published genome. Primers names were simplified (“ctnpg1_” is not mentioned). The ctnpg1_5in is red, the ctnpg1_3in is green, and all other primers are black. **c.** Agarose gel electrophoresis done as in **a.** for the primer combination indicated over each lane. In the order shown, the expected sizes are 7.5, 3.5, 3.0, 3.5, 5.0, 6.5, and 10 Kb. **d.** Agarose gel electrophoresis as above for *rrn* operons in 22 *P. gingivalis* strains. For PCR conditions, primer sequences, and strain origins, see Methods. (PDF 194 kb)

Additional file 9: Figure S7. Whole-genome alignments of the three resequenced *P. gingivalis* strains. Published and *de novo* assembled genome architectures are compared. Locally collinear blocks (LCBs) were detected using the progressiveMauve algorithm. Shown are translocations and inversions, insertions (green arrows), and deletions (thin red arrows), along with size differences and SNP counts. For ATCC 33277, the blue blocks represent CTnPg1 copies; in TDC60, they are the *rrn* operons. For W83, the *de novo* sequence “a” was assembled from the published genome’s “b” and “c” sequences (see text for details). (PDF 27 kb)

Additional file 10: Figure S8. Insertions and deletions in the *de novo P. gingivalis* assemblies as compared to the published genomes. In all cases, alignment zooms are presented for the corresponding insertions or deletions detailed in Fig. S8 and in the text. The upper genome is the *de novo* assembly and the bottom is the published one. **a.** The ATCC 33277 strain has four indels. **b.** TDC has four indels, with an additional 22-bp deletion in an intergenic region (not depicted). **c.** W83 has a single insertion. (PDF 65 kb)

Additional file 11: Table S4. Classification of additional CDS/pseudogenes. After a manual biocurated annotation, the changes were separated into five functional categories. The absolute counts of the new CDS/pseudogenes, CDS changed to pseudogenes or vice versa, feature fusions or splitting of single features into two, and coding strand changes are presented here, strain by strain. The results are emphasised via a heatmap that goes from lilac to burgundy. For re-annotation of the CDS/pseudogenes that are shared between the two versions, see Results and Fig. 4. (PDF 84 kb)

Additional file 12: Figure S9. The three *P. gingivalis* strains binned by their CDS/pseudogene functions. The coding sequences and pseudogenes were classified into five categories as shown, and the histogram is based on their absolute counts. (PDF 58 kb)

Additional file 13: Figure S10. CDS/pseudogenes of all strains that have at least two copies in all three *P. gingivalis* genomes. a. Horizontal histogram of absolute gene counts binned by category. Three of these categories are related to nucleic acids (DNA helicases, regulators mainly containing the HTH 17 domain, and histones), while the remaining ones are related to mobile elements (integrases, transposases, tetracycline-resistance genes, and the *tra* conjugative transposons). **b.** Histogram of all transposases coded by genome and divided into CDS and pseudogenes. **c.** Heatmap table of transposase families separated into coding sequences and pseudogenes. Absolute numbers are presented, as the copy numbers grow, the cell color move from light blue to dark red. (PDF 83 kb)

Additional file 14: Figure S11. Overview of the core genomes of *P. gingivalis* strains ATCC 33277, TDC60, and W83. **a.** Pie chart of genes present in all three strains grouped by categories: constant (more than 97% nucleotide identity, none has paralogs); variable (less than 97% nucleotide identity, none have paralogs); with paralogs (at least one strain has paralogs); and with pseudogenisation (at least one strain has a pseudogene, another a functional CDS, and none have paralogs). **b.** Constant core genes classified into five categories. **c.** Genes in variable core genome. The 47 genes are presented grouped by function. **d.** Core genes with paralogs. Gene names and products are listed, and the number of paralogs detailed by strain. To facilitate reading, cells were shaded when at least two paralogs exist. *, pseudogenes; †, a hypothetical gene clustered with genes from the BF0131 conjugative transposon. (PDF 26 kb)

Additional file 15: Table S5. Core genes with pseudogenisation. Gene names and products are listed for all 36 genes that are present in this subset of core genes. An asterisk indicates the strains in which they are pseudogenised, and genes with pseudogenes in more than one strain are bold. (PDF 35 kb)

Additional file 16: Table S3. CDS/pseudogene differences by strain. Comparison of the publically available annotations accessed via NCBI versus this study’s annotation showed that coding sequences changed to pseudogenes, pseudogenes changed to CDS, features fused, single features split into two, and coding strands changed. See the Results for information on shared CDS and pseudogenes, eliminated CDS/genes, and all other features (tRNA, rRNA, ncRNA, tmRNA, riboswitches, mobile elements, signal peptides, and repeat regions). (PDF 66 kb)

## Abbreviations

ANI: Average Nucleotide Identity
CDS: Coding DNA Sequence
COG: Cluster of Orthologous Groups (of proteins)
CRISPR: Clustered Regularly Interspaced Short Palindromic Repeats
CTnPg: Conjugative Transposon of Porphyromonas gingivalis
DUF: Domain of Unknown Function
ELF: Evil-Little Fellows
FIG: Fellowship for Interpretation of Genomes
GEBA: Genomic Encyclopedia of Bacteria and Archaea
HGT: Horizontal Gene Transfer
ICE: Integrative and Conjugative Element
IQR: Interquartile Ranges
IS: Insertion Sequence
LCB: Locally Collinear Blocks
ME: Mobile Element
MITE: Miniature Inverted-repeat Transposable Elements
NAP: Nucleoid-Associated Proteins
NCBI: National Center for Biotechnology Information
NGS: Next Generation Sequencing, also SGS or massively parallel sequencing
NNI: Nearest-Neighbor Interchange
OLC: Overlap-Layout-Consensus
ORF: Open Reading Frame
*rrn*: ribosomal RNA operon
SGS: Second Generatation Sequencing, also NGS or massively parallel sequencing
SNP: Single Nucleotide Polymorphism
SPR: Subtree Pruning and Regrafting
WGS: Whole Genome Sequencing

## References

[1] Qin J, Li R, Raes J, Arumugam M, Burgdorf KS, Manichanh C, Nielsen T, Pons N, Levenez F, Yamada T (2010). A human gut microbial gene catalogue established by metagenomic sequencing. Nature.

[2] Human Microbiome Project Consortium (2012). Structure, function and diversity of the healthy human microbiome. Nature.

[3] Hugon P, Dufour JC, Colson P, Fournier PE, Sallah K, Raoult D (2015). A comprehensive repertoire of prokaryotic species identified in human beings. The Lancet Infectious diseases.

[4] Lloyd-Price J, Abu-Ali G, Huttenhower C (2016). The healthy human microbiome. Genome medicine.

[5] Lozupone CA, Stombaugh JI, Gordon JI, Jansson JK, Knight R (2012). Diversity, stability and resilience of the human gut microbiota. Nature.

[6] Robles Alonso V, Guarner F (2013). Linking the gut microbiota to human health. The British journal of nutrition.

[7] Winter SE, Baumler AJ (2014). Why related bacterial species bloom simultaneously in the gut: principles underlying the 'Like will to like' concept. Cellular microbiology.

[8] Hahnke RL, Meier-Kolthoff JP, Garcia-Lopez M, Mukherjee S, Huntemann M, Ivanova NN, Woyke T, Kyrpides NC, Klenk HP, Goker M (2016). Genome-Based Taxonomic Classification of Bacteroidetes. Frontiers in microbiology.

[9] Arumugam M, Raes J, Pelletier E, Le Paslier D, Yamada T, Mende DR, Fernandes GR, Tap J, Bruls T, Batto JM (2011). Enterotypes of the human gut microbiome. Nature.

[10] Bull MJ, Plummer NT (2014). Part 1: The Human Gut Microbiome in Health and Disease. Integrative medicine (Encinitas, Calif).

[11] Johnson EL, Heaver SL, Walters WA, Ley RE (2017). Microbiome and metabolic disease: revisiting the bacterial phylum Bacteroidetes. Journal of molecular medicine (Berlin, Germany).

[12] Oren A, da Costa MS, Garrity GM, Rainey FA, Rossello-Mora R, Schink B, Sutcliffe I, Trujillo ME, Whitman WB (2015). Proposal to include the rank of phylum in the International Code of Nomenclature of Prokaryotes. Int J Syst Evol Microbiol.

[13] Krieg NR, Ludwig W, Euzéby J, Whitman WB: Phylum XIV. Bacteroidetes phyl. nov. In: *Bergey’s Manual® of Systematic Bacteriology: Volume Four The Bacteroidetes, Spirochaetes, Tenericutes (Mollicutes), Acidobacteria, Fibrobacteres, Fusobacteria, Dictyoglomi, Gemmatimonadetes, Lentisphaerae, Verrucomicrobia, Chlamydiae, and Planctomycetes.* Edited by Krieg NR, Staley JT, Brown DR, Hedlund BP, Paster BJ, Ward NL, Ludwig W, Whitman WB. New York, NY: Springer New York; 2010: 25-469.

[14] Thomas F, Hehemann JH, Rebuffet E, Czjzek M, Michel G (2011). Environmental and gut bacteroidetes: the food connection. Frontiers in microbiology.

[15] Muñoz R, Rossello-Mora R, Amann R (2016). Revised phylogeny of Bacteroidetes and proposal of sixteen new taxa and two new combinations including Rhodothermaeota phyl. nov. Syst Appl Microbiol.

[16] Smith DR (2017). Goodbye genome paper, hello genome report: the increasing popularity of 'genome announcements' and their impact on science. Briefings in functional genomics.

[17] Reuter JA, Spacek DV, Snyder MP (2015). High-throughput sequencing technologies. Molecular cell.

[18] Heather JM, Chain B (2016). The sequence of sequencers: The history of sequencing DNA. Genomics.

[19] Kyrpides NC, Hugenholtz P, Eisen JA, Woyke T, Goker M, Parker CT, Amann R, Beck BJ, Chain PS, Chun J (2014). Genomic encyclopedia of bacteria and archaea: sequencing a myriad of type strains. PLoS biology.

[20] Wu D, Hugenholtz P, Mavromatis K, Pukall R, Dalin E, Ivanova NN, Kunin V, Goodwin L, Wu M, Tindall BJ (2009). A phylogeny-driven genomic encyclopaedia of Bacteria and Archaea. Nature.

[21] Ventura M, Canchaya C, Fitzgerald GF, Gupta RS, van Sinderen D (2007). Genomics as a means to understand bacterial phylogeny and ecological adaptation: the case of bifidobacteria. Antonie van Leeuwenhoek.

[22] Forde BM, O'Toole PW (2013). Next-generation sequencing technologies and their impact on microbial genomics. Briefings in functional genomics.

[23] Goodwin S, McPherson JD, McCombie WR (2016). Coming of age: ten years of next-generation sequencing technologies. Nature reviews Genetics.

[24] Li Z, Chen Y, Mu D, Yuan J, Shi Y, Zhang H, Gan J, Li N, Hu X, Liu B (2012). Comparison of the two major classes of assembly algorithms: overlap-layout-consensus and de-bruijn-graph. Briefings in functional genomics.

[25] Treangen TJ, Salzberg SL (2011). Repetitive DNA and next-generation sequencing: computational challenges and solutions. Nature reviews Genetics.

[26] Williams D, Trimble WL, Shilts M, Meyer F, Ochman H (2013). Rapid quantification of sequence repeats to resolve the size, structure and contents of bacterial genomes. BMC genomics.

[27] Kamada M, Hase S, Sato K, Toyoda A, Fujiyama A, Sakakibara Y (2014). Whole genome complete resequencing of Bacillus subtilis natto by combining long reads with high-quality short reads. PloS one.

[28] Shapiro JA, von Sternberg R (2005). Why repetitive DNA is essential to genome function. Biological reviews of the Cambridge Philosophical Society.

[29] Avershina E, Rudi K (2015). Dominant short repeated sequences in bacterial genomes. Genomics.

[30] Nagarajan N, Cook C, Di Bonaventura M, Ge H, Richards A, Bishop-Lilly KA, DeSalle R, Read TD, Pop M (2010). Finishing genomes with limited resources: lessons from an ensemble of microbial genomes. BMC genomics.

[31] Bao E, Jiang T, Girke T (2014). AlignGraph: algorithm for secondary de novo genome assembly guided by closely related references. Bioinformatics (Oxford, England).

[32] Kolmogorov M, Raney B, Paten B, Pham S (2014). Ragout-a reference-assisted assembly tool for bacterial genomes. Bioinformatics (Oxford, England).

[33] Salzberg SL, Yorke JA (2005). Beware of mis-assembled genomes. Bioinformatics (Oxford, England).

[34] Vincent AT, Derome N, Boyle B, Culley AI, Charette SJ. Next-generation sequencing (NGS) in the microbiological world: How to make the most of your money. Journal of microbiological methods. 2016; 10.1016/j.mimet.2016.02.016 26995332

[35] Dayarian A, Michael TP, Sengupta AM (2010). SOPRA: Scaffolding algorithm for paired reads via statistical optimization. BMC bioinformatics.

[36] English AC, Richards S, Han Y, Wang M, Vee V, Qu J, Qin X, Muzny DM, Reid JG, Worley KC (2012). Mind the gap: upgrading genomes with Pacific Biosciences RS long-read sequencing technology. PloS one.

[37] Madoui MA, Engelen S, Cruaud C, Belser C, Bertrand L, Alberti A, Lemainque A, Wincker P, Aury JM (2015). Genome assembly using Nanopore-guided long and error-free DNA reads. BMC genomics.

[38] Mariano DC, Sousa Tde J, Pereira FL, Aburjaile F, Barh D, Rocha F, Pinto AC, Hassan SS, Saraiva TD, Dorella FA (2016). Whole-genome optical mapping reveals a mis-assembly between two rRNA operons of Corynebacterium pseudotuberculosis strain 1002. BMC genomics.

[39] Madoui MA, Dossat C, d'Agata L, van Oeveren J, van der Vossen E, Aury JM (2016). MaGuS: a tool for quality assessment and scaffolding of genome assemblies with Whole Genome Profiling Data. BMC bioinformatics.

[40] Land M, Hauser L, Jun SR, Nookaew I, Leuze MR, Ahn TH, Karpinets T, Lund O, Kora G, Wassenaar T (2015). Insights from 20 years of bacterial genome sequencing. Functional & integrative genomics.

[41] Howison M, Zapata F, Dunn CW (2013). Toward a statistically explicit understanding of de novo sequence assembly. Bioinformatics (Oxford, England).

[42] Barbosa EG, Aburjaile FF, Ramos RT, Carneiro AR, Le Loir Y, Baumbach J, Miyoshi A, Silva A, Azevedo V (2014). Value of a newly sequenced bacterial genome. World journal of biological chemistry.

[43] Stepanov VG, Tirumalai MR, Montazari S, Checinska A, Venkateswaran K, Fox GE (2016). Bacillus pumilus SAFR-032 Genome Revisited: Sequence Update and Re-Annotation. PloS one.

[44] Klassen JL, Currie CR (2012). Gene fragmentation in bacterial draft genomes: extent, consequences and mitigation. BMC genomics.

[45] Fierst JL (2015). Using linkage maps to correct and scaffold de novo genome assemblies: methods, challenges, and computational tools. Frontiers in genetics.

[46] Turroni F, van Sinderen D, Ventura M (2011). Genomics and ecological overview of the genus Bifidobacterium. International journal of food microbiology.

[47] Periwal V, Scaria V (2015). Insights into structural variations and genome rearrangements in prokaryotic genomes. Bioinformatics (Oxford, England).

[48] R Core Team: R: A Language and Environment for Statistical Computing. In. Vienna, Austria; 2016.

[49] Katoh K, Misawa K, Kuma K, Miyata T (2002). MAFFT: a novel method for rapid multiple sequence alignment based on fast Fourier transform. Nucleic acids research.

[50] Kearse M, Moir R, Wilson A, Stones-Havas S, Cheung M, Sturrock S, Buxton S, Cooper A, Markowitz S, Duran C (2012). Geneious Basic: an integrated and extendable desktop software platform for the organization and analysis of sequence data. Bioinformatics (Oxford, England).

[51] Guindon S, Gascuel O (2003). A simple, fast, and accurate algorithm to estimate large phylogenies by maximum likelihood. Systematic biology.

[52] Hasegawa M, Kishino H, Yano T (1985). Dating of the human-ape splitting by a molecular clock of mitochondrial DNA. Journal of molecular evolution.

[53] Lee I, Kim YO, Park SC, Chun J. OrthoANI: An improved algorithm and software for calculating average nucleotide identity. International journal of systematic and evolutionary microbiology. 2015; 10.1099/ijsem.0.000760 26585518

[54] Treangen TJ, Darling AE, Achaz G, Ragan MA, Messeguer X, Rocha EP (2009). A novel heuristic for local multiple alignment of interspersed DNA repeats. IEEE/ACM transactions on computational biology and bioinformatics.

[55] Krzywinski M, Schein J, Birol I, Connors J, Gascoyne R, Horsman D, Jones SJ, Marra MA (2009). Circos: an information aesthetic for comparative genomics. Genome research.

[56] Huang W, Li L, Myers JR, Marth GT (2012). ART: a next-generation sequencing read simulator. Bioinformatics (Oxford, England).

[57] Coil D, Jospin G, Darling AE (2015). A5-miseq: an updated pipeline to assemble microbial genomes from Illumina MiSeq data. Bioinformatics (Oxford, England).

[58] Li H: Exploring single-sample SNP and INDEL calling with whole-genome de novo assembly. Bioinformatics (Oxford, England) 2012, 28(14):1838-1844. 10.1093/bioinformatics/bts280 PMC3389770 22569178

[59] Chikhi R, Rizk G (2013). Space-efficient and exact de Bruijn graph representation based on a Bloom filter. Algorithms for molecular biology : AMB.

[60] Chevreux B, Pfisterer T, Drescher B, Driesel AJ, Muller WE, Wetter T, Suhai S (2004). Using the miraEST assembler for reliable and automated mRNA transcript assembly and SNP detection in sequenced ESTs. Genome research.

[61] Zhu X, Leung HC, Chin FY, Yiu SM, Quan G, Liu B, Wang Y (2014). PERGA: a paired-end read guided de novo assembler for extending contigs using SVM and look ahead approach. PloS one.

[62] Luo R, Liu B, Xie Y, Li Z, Huang W, Yuan J, He G, Chen Y, Pan Q, Liu Y (2012). SOAPdenovo2: an empirically improved memory-efficient short-read de novo assembler. GigaScience.

[63] Bankevich A, Nurk S, Antipov D, Gurevich AA, Dvorkin M, Kulikov AS, Lesin VM, Nikolenko SI, Pham S, Prjibelski AD (2012). SPAdes: a new genome assembly algorithm and its applications to single-cell sequencing. Journal of computational biology : a journal of computational molecular cell biology.

[64] Zerbino DR, Birney E (2008). Velvet: algorithms for de novo short read assembly using de Bruijn graphs. Genome research.

[65] Zhang J, Kobert K, Flouri T, Stamatakis A (2014). PEAR: a fast and accurate Illumina Paired-End reAd mergeR. Bioinformatics (Oxford, England).

[66] Chikhi R, Medvedev P (2014). Informed and automated k-mer size selection for genome assembly. Bioinformatics (Oxford, England).

[67] Gurevich A, Saveliev V, Vyahhi N, Tesler G (2013). QUAST: quality assessment tool for genome assemblies. Bioinformatics (Oxford, England).

[68] Mariette J, Escudie F, Allias N, Salin G, Noirot C, Thomas S, Klopp C (2012). NG6: Integrated next generation sequencing storage and processing environment. BMC genomics.

[69] Koren S, Walenz BP, Berlin K, Miller JR, Bergman NH, Phillippy AM (2017). Canu: scalable and accurate long-read assembly via adaptive k-mer weighting and repeat separation. Genome research.

[70] Perez-Chaparro PJ, Lafaurie GI, Gracieux P, Meuric V, Tamanai-Shacoori Z, Castellanos JE, Bonnaure-Mallet M (2009). Distribution of Porphyromonas gingivalis fimA genotypes in isolates from subgingival plaque and blood sample during bacteremia. Biomedica : revista del Instituto Nacional de Salud.

[71] Seemann T (2014). Prokka: rapid prokaryotic genome annotation. Bioinformatics (Oxford, England).

[72] Kremer FS, Eslabao MR, Dellagostin OA, Pinto LD. Genix: a new online automated pipeline for bacterial genome annotation. FEMS microbiology letters. 2016;363(23) 10.1093/femsle/fnw263 27856568

[73] Brettin T, Davis JJ, Disz T, Edwards RA, Gerdes S, Olsen GJ, Olson R, Overbeek R, Parrello B, Pusch GD (2015). RASTtk: a modular and extensible implementation of the RAST algorithm for building custom annotation pipelines and annotating batches of genomes. Scientific reports.

[74] Gray KA, Yates B, Seal RL, Wright MW, Bruford EA. Genenames.org: the HGNC resources in 2015. Nucleic acids research 2015. 43(Database issue):D1079–85. 10.1093/nar/gku1071 PMC4383909 25361968

[75] Altschul SF, Gish W, Miller W, Myers EW, Lipman DJ (1990). Basic local alignment search tool. Journal of molecular biology.

[76] Marchler-Bauer A, Bryant SH: CD-Search: protein domain annotations on the fly. Nucleic acids research 2004, 32(Web Server issue):W327-331. 10.1093/nar/gkh454 PMC441592 15215404

[77] Vallenet D, Belda E, Calteau A, Cruveiller S, Engelen S, Lajus A, Le Fevre F, Longin C, Mornico D, Roche D (2013). MicroScope--an integrated microbial resource for the curation and comparative analysis of genomic and metabolic data. Nucleic acids research.

[78] Petersen TN, Brunak S, von Heijne G, Nielsen H (2011). SignalP 4.0: discriminating signal peptides from transmembrane regions. Nature methods.

[79] Gomi M, Sonoyama M, Mitaku S (2004). High performance system for signal peptide prediction: SOSUIsignal. Chem-bio informatics. journal.

[80] NY Y, Wagner JR, Laird MR, Melli G, Rey S, Lo R, Dao P, Sahinalp SC, Ester M, Foster LJ (2010). PSORTb 3.0: improved protein subcellular localization prediction with refined localization subcategories and predictive capabilities for all prokaryotes. Bioinformatics (Oxford, England).

[81] CS Y, Lin CJ, Hwang JK (2004). Predicting subcellular localization of proteins for Gram-negative bacteria by support vector machines based on n-peptide compositions. Protein science : a publication of the Protein Society.

[82] Berven FS, Flikka K, Jensen HB, Eidhammer I: BOMP: a program to predict integral beta-barrel outer membrane proteins encoded within genomes of Gram-negative bacteria. Nucleic acids research 2004, 32(Web Server issue):W394-399. 10.1093/nar/gkh351 PMC441489 15215418

[83] Juncker AS, Willenbrock H, Von Heijne G, Brunak S, Nielsen H, Krogh A (2003). Prediction of lipoprotein signal peptides in Gram-negative bacteria. Protein science : a publication of the Protein Society.

[84] Babu MM, Priya ML, Selvan AT, Madera M, Gough J, Aravind L, Sankaran K (2006). A database of bacterial lipoproteins (DOLOP) with functional assignments to predicted lipoproteins. Journal of bacteriology.

[85] Siguier P, Perochon J, Lestrade L, Mahillon J, Chandler M (2006). ISfinder: the reference centre for bacterial insertion sequences. Nucleic acids research.

[86] Grissa I, Vergnaud G, Pourcel C: CRISPRFinder: a web tool to identify clustered regularly interspaced short palindromic repeats. Nucleic acids research 2007, 35(Web Server issue):W52-57. 10.1093/nar/gkm360 PMC1933234 17537822

[87] Biswas A, Staals RH, Morales SE, Fineran PC, Brown CM (2016). CRISPRDetect: A flexible algorithm to define CRISPR arrays. BMC genomics.

[88] Rousseau C, Gonnet M, Le Romancer M, Nicolas J (2009). CRISPI: a CRISPR interactive database. Bioinformatics (Oxford, England).

[89] Bland C, Ramsey TL, Sabree F, Lowe M, Brown K, Kyrpides NC, Hugenholtz P (2007). CRISPR recognition tool (CRT): a tool for automatic detection of clustered regularly interspaced palindromic repeats. BMC bioinformatics.

[90] Bertelli C, Laird MR, Williams KP, Lau BY, Hoad G, Winsor GL, Brinkman FS. IslandViewer 4: expanded prediction of genomic islands for larger-scale datasets. Nucleic acids research. 2017; 10.1093/nar/gkx343 PMC5570257 28472413

[91] Waack S, Keller O, Asper R, Brodag T, Damm C, Fricke WF, Surovcik K, Meinicke P, Merkl R (2006). Score-based prediction of genomic islands in prokaryotic genomes using hidden Markov models. BMC bioinformatics.

[92] Hsiao W, Wan I, Jones SJ, Brinkman FS (2003). IslandPath: aiding detection of genomic islands in prokaryotes. Bioinformatics (Oxford, England).

[93] Langille MG, Hsiao WW, Brinkman FS (2008). Evaluation of genomic island predictors using a comparative genomics approach. BMC bioinformatics.

[94] Che D, Hasan MS, Wang H, Fazekas J, Huang J, Liu Q (2011). EGID: an ensemble algorithm for improved genomic island detection in genomic sequences. Bioinformation.

[95] Vernikos GS, Parkhill J (2006). Interpolated variable order motifs for identification of horizontally acquired DNA: revisiting the Salmonella pathogenicity islands. Bioinformatics (Oxford, England).

[96] Shrivastava S, Reddy CV, Mande SS (2010). INDeGenIUS, a new method for high-throughput identification of specialized functional islands in completely sequenced organisms. Journal of biosciences.

[97] Tu Q, Ding D (2003). Detecting pathogenicity islands and anomalous gene clusters by iterative discriminant analysis. FEMS microbiology letters.

[98] Darling AE, Mau B, Perna NT (2010). progressiveMauve: multiple genome alignment with gene gain, loss and rearrangement. PloS one.

[99] Konstantinidis KT, Ramette A, Tiedje JM (2006). The bacterial species definition in the genomic era. Philosophical transactions of the Royal Society of London Series B, Biological sciences.

[100] Naito M, Hirakawa H, Yamashita A, Ohara N, Shoji M, Yukitake H, Nakayama K, Toh H, Yoshimura F, Kuhara S (2008). Determination of the genome sequence of Porphyromonas gingivalis strain ATCC 33277 and genomic comparison with strain W83 revealed extensive genome rearrangements in P. gingivalis. DNA research : an international journal for rapid publication of reports on genes and genomes.

[101] Chastain-Gross RP, Xie G, Belanger M, Kumar D, Whitlock JA, Liu L, Raines SM, Farmerie WG, Daligault HE, Han CS et al: Genome Sequence of Porphyromonas gingivalis Strain 381. Genome Announc 2017, 5(2). 10.1128/genomeA.01467-16 PMC5256213 28082501

[102] Dashper SG, Mitchell HL, Seers CA, Gladman SL, Seemann T, Bulach DM, Chandry PS, Cross KJ, Cleal SM, Reynolds EC (2017). Porphyromonas gingivalis Uses Specific Domain Rearrangements and Allelic Exchange to Generate Diversity in Surface Virulence Factors. Frontiers in microbiology.

[103] Klein BA, Chen T, Scott JC, Koenigsberg AL, Duncan MJ, LT H (2015). Identification and characterization of a minisatellite contained within a novel miniature inverted-repeat transposable element (MITE) of Porphyromonas gingivalis. Mobile DNA.

[104] Hovik H, WH Y, Olsen I, Chen T (2012). Comprehensive transcriptome analysis of the periodontopathogenic bacterium Porphyromonas gingivalis W83. Journal of bacteriology.

[105] Ochman H (2002). Distinguishing the ORFs from the ELFs: short bacterial genes and the annotation of genomes. Trends in genetics : TIG.

[106] Lawrence J (2003). When ELFs are ORFs, but don't act like them. Trends in genetics : TIG.

[107] Fattash I, Rooke R, Wong A, Hui C, Luu T, Bhardwaj P, Yang G (2013). Miniature inverted-repeat transposable elements: discovery, distribution, and activity. Genome.

[108] Bocs S, Danchin A, Medigue C (2002). Re-annotation of genome microbial coding-sequences: finding new genes and inaccurately annotated genes. BMC bioinformatics.

[109] Califano JV, Kitten T, Lewis JP, Macrina FL, Fleischmann RD, Fraser CM, Duncan MJ, Dewhirst FE (2000). Characterization of Porphyromonas gingivalis insertion sequence-like element ISPg5. Infection and immunity.

[110] Szafranski SP, Winkel A, Stiesch M (2017). The use of bacteriophages to biocontrol oral biofilms. Journal of biotechnology.

[111] Lenhart JS, Schroeder JW, Walsh BW, Simmons LA (2012). DNA repair and genome maintenance in Bacillus subtilis. Microbiology and molecular biology reviews : MMBR.

[112] Heinken A, Sahoo S, Fleming RM, Thiele I (2013). Systems-level characterization of a host-microbe metabolic symbiosis in the mammalian gut. Gut microbes.

[113] Garza DR, Van Verk MC, Huynen MA, Dutilh BE: Bottom-up ecology of the human microbiome: from metagenomes to metabolomes. bioRxiv 2016:060673.

[114] Magnusdottir S, Heinken A, Kutt L, Ravcheev DA, Bauer E, Noronha A, Greenhalgh K, Jager C, Baginska J, Wilmes P (2017). Generation of genome-scale metabolic reconstructions for 773 members of the human gut microbiota. Nature biotechnology.

[115] Papanicolaou A (2016). The life cycle of a genome project: perspectives and guidelines inspired by insect genome projects. F1000Research.

[116] Field D, Wilson G, van der Gast C (2006). How do we compare hundreds of bacterial genomes?. Current opinion in microbiology.

[117] Tatusova T, Ciufo S, Fedorov B, O'Neill K, Tolstoy I (2014). RefSeq microbial genomes database: new representation and annotation strategy. Nucleic acids research.

[118] Mukherjee S, Seshadri R, Varghese NJ, Eloe-Fadrosh EA, Meier-Kolthoff JP, Goker M, Coates RC, Hadjithomas M, Pavlopoulos GA, Paez-Espino D (2017). 1,003 reference genomes of bacterial and archaeal isolates expand coverage of the tree of life. Nature biotechnology.

[119] Galperin MY, Koonin EV (2010). From complete genome sequence to 'complete' understanding?. Trends in biotechnology.

[120] Siddiqui H, Yoder-Himes DR, Mizgalska D, Nguyen KA, Potempa J, Olsen I. Genome Sequence of Porphyromonas gingivalis Strain HG66 (DSM 28984). Genome announcements. 2014;2(5) 10.1128/genomeA.00947-14 PMC4175203 25291768

[121] McLean JS, Lombardo MJ, Ziegler MG, Novotny M, Yee-Greenbaum J, Badger JH, Tesler G, Nurk S, Lesin V, Brami D (2013). Genome of the pathogen Porphyromonas gingivalis recovered from a biofilm in a hospital sink using a high-throughput single-cell genomics platform. Genome research.

[122] Chen T, Siddiqui H, Olsen I (2017). silico Comparison of 19 Porphyromonas gingivalis Strains in Genomics, Phylogenetics, Phylogenomics and Functional Genomics. Frontiers in cellular and infection microbiology.

[123] Mavromatis K, Land ML, Brettin TS, Quest DJ, Copeland A, Clum A, Goodwin L, Woyke T, Lapidus A, Klenk HP (2012). The fast changing landscape of sequencing technologies and their impact on microbial genome assemblies and annotation. PloS one.

[124] Utturkar SM, Klingeman DM, Hurt RA, Jr., Brown SD: A Case Study into Microbial Genome Assembly Gap Sequences and Finishing Strategies. Frontiers in microbiology 2017, 8:1272. 10.3389/fmicb.2017.01272 PMC5513972 28769883

[125] Fadeev E, De Pascale F, Vezzi A, Hubner S, Aharonovich D, Sher D (2016). Why Close a Bacterial Genome? The Plasmid of Alteromonas Macleodii HOT1A3 is a Vector for Inter-Specific Transfer of a Flexible Genomic Island. Frontiers in microbiology.

[126] Kryukov K, Imanishi T (2016). Human Contamination in Public Genome Assemblies. PloS one.

[127] Mallet L, Bitard-Feildel T, Cerutti F, Chiapello H. PhylOligo: a package to identify contaminant or untargeted organism sequences in genome assemblies. Bioinformatics (Oxford, England). 2017; 10.1093/bioinformatics/btx396 PMC5860033 28637232

[128] Thomma B, Seidl MF, Shi-Kunne X, Cook DE, Bolton MD, van Kan JAL, Faino L (2016). Mind the gap; seven reasons to close fragmented genome assemblies. Fungal genetics and biology : FG & B.

[129] Mardis E, McPherson J, Martienssen R, Wilson RK, McCombie WR (2002). What is finished, and why does it matter. Genome research.

[130] Riba-Grognuz O, Keller L, Falquet L, Xenarios I, Wurm Y (2011). Visualization and quality assessment of de novo genome assemblies. Bioinformatics (Oxford, England).

[131] Baker M (2012). De novo genome assembly: what every biologist should know. Nat Meth.

[132] Khiste N, Ilie L (2015). LASER: Large genome ASsembly EvaluatoR. BMC research notes.

[133] Earl D, Bradnam K, St John J, Darling A, Lin D, Fass J, HO Y, Buffalo V, Zerbino DR, Diekhans M (2011). Assemblathon 1: a competitive assessment of de novo short read assembly methods. Genome research.

[134] Dias Z, Dias U, Setubal JC (2012). SIS: a program to generate draft genome sequence scaffolds for prokaryotes. BMC bioinformatics.

[135] Magoc T, Pabinger S, Canzar S, Liu X, Su Q, Puiu D, Tallon LJ, Salzberg SL (2013). GAGE-B: an evaluation of genome assemblers for bacterial organisms. Bioinformatics (Oxford, England).

[136] Mikheenko A, Valin G, Prjibelski A, Saveliev V, Gurevich A (2016). Icarus: visualizer for de novo assembly evaluation. Bioinformatics (Oxford, England).

[137] Loman NJ, Misra RV, Dallman TJ, Constantinidou C, Gharbia SE, Wain J, Pallen MJ (2012). Performance comparison of benchtop high-throughput sequencing platforms. Nature biotechnology.

[138] Hunt M, Newbold C, Berriman M, Otto TD (2014). A comprehensive evaluation of assembly scaffolding tools. Genome biology.

[139] Treangen TJ, Abraham AL, Touchon M, Rocha EP (2009). Genesis, effects and fates of repeats in prokaryotic genomes. FEMS microbiology reviews.

[140] Touchon M, Rocha EP (2007). Causes of insertion sequences abundance in prokaryotic genomes. Molecular biology and evolution.

[141] Newton IL, Bordenstein SR (2011). Correlations between bacterial ecology and mobile DNA. Current microbiology.

[142] Schatz MC, Delcher AL, Salzberg SL (2010). Assembly of large genomes using second-generation sequencing. Genome research.

[143] Denton JF, Lugo-Martinez J, Tucker AE, Schrider DR, Warren WC, Hahn MW (2014). Extensive error in the number of genes inferred from draft genome assemblies. PLoS computational biology.

[144] Enersen M, Olsen I, van Winkelhoff AJ, Caugant DA (2006). Multilocus sequence typing of Porphyromonas gingivalis strains from different geographic origins. Journal of clinical microbiology.

[145] Igboin CO, Griffen AL, Leys EJ (2009). Porphyromonas gingivalis strain diversity. Journal of clinical microbiology.

[146] Enersen M. Porphyromonas gingivalis: a clonal pathogen?: Diversities in housekeeping genes and the major fimbriae gene. Journal of oral microbiology. 2011;3 10.3402/jom.v3i0.8487 PMC3223970 22125739

[147] Dolgilevich S, Rafferty B, Luchinskaya D, Kozarov E. Genomic comparison of invasive and rare non-invasive strains reveals Porphyromonas gingivalis genetic polymorphisms. Journal of oral microbiology. 2011;3 10.3402/jom.v3i0.5764 PMC3086587 21541093

[148] Tribble GD, Rigney TW, Dao DH, Wong CT, Kerr JE, Taylor BE, Pacha S, Kaplan HB. Natural competence is a major mechanism for horizontal DNA transfer in the oral pathogen Porphyromonas gingivalis. mBio. 2012;3(1) 10.1128/mBio.00231-11 PMC3268665 22294679

[149] Tribble GD, Lamont GJ, Progulske-Fox A, Lamont RJ (2007). Conjugal transfer of chromosomal DNA contributes to genetic variation in the oral pathogen Porphyromonas gingivalis. Journal of bacteriology.

[150] Feil EJ, Spratt BG (2001). Recombination and the population structures of bacterial pathogens. Annual review of microbiology.

[151] Tibayrenc M, Ayala FJ (2015). How clonal are Neisseria species? The epidemic clonality model revisited. Proceedings of the National Academy of Sciences of the United States of America.

[152] Riedel T, Bunk B, Thurmer A, Sproer C, Brzuszkiewicz E, Abt B, Gronow S, Liesegang H, Daniel R, Overmann J. Genome Resequencing of the Virulent and Multidrug-Resistant Reference Strain Clostridium difficile 630. Genome announcements. 2015;3(2) 10.1128/genomeA.00276-15 PMC4392158 25858846

[153] Malone KM, Farrell D, Stuber TP, Schubert OT, Aebersold R, Robbe-Austerman S, Gordon SV. Updated Reference Genome Sequence and Annotation of Mycobacterium bovis AF2122/97. Genome announcements. 2017;5(14) 10.1128/genomeA.00157-17 PMC5383904 28385856

[154] Chen T, Hosogi Y, Nishikawa K, Abbey K, Fleischmann RD, Walling J, Duncan MJ (2004). Comparative whole-genome analysis of virulent and avirulent strains of Porphyromonas gingivalis. Journal of bacteriology.

[155] Slots J, Gibbons RJ (1978). Attachment of Bacteroides melaninogenicus subsp. asaccharolyticus to oral surfaces and its possible role in colonization of the mouth and of periodontal pockets. Infection and immunity.

[156] Loos BG, Mayrand D, Genco RJ, Dickinson DP (1990). Genetic heterogeneity of Porphyromonas (Bacteroides) gingivalis by genomic DNA fingerprinting. Journal of dental research.

[157] Guo FB, Xiong L, Teng JL, Yuen KY, Lau SK, Woo PC (2013). Re-annotation of protein-coding genes in 10 complete genomes of Neisseriaceae family by combining similarity-based and composition-based methods. DNA research : an international journal for rapid publication of reports on genes and genomes.

[158] Zhang HX, Li SJ, Zhou HQ (2014). Evaluating the annotation of protein-coding genes in bacterial genomes: Chloroflexus aurantiacus strain J-10-fl and Natrinema sp J7-2 as case studies. Genetics and molecular research : GMR.

[159] Richardson EJ, Watson M (2013). The automatic annotation of bacterial genomes. Briefings in bioinformatics.

[160] Indrischek H, Wieseke N, Stadler PF, Prohaska SJ (2016). The paralog-to-contig assignment problem: high quality gene models from fragmented assemblies. Algorithms for molecular biology : AMB.

[161] Vernikos G, Medini D, Riley DR, Tettelin H (2015). Ten years of pan-genome analyses. Current opinion in microbiology.

[162] Touchon M, Rocha EP (2016). Coevolution of the Organization and Structure of Prokaryotic Genomes. Cold Spring Harbor perspectives in biology.

[163] Brunner J, Wittink FR, Jonker MJ, de Jong M, Breit TM, Laine ML, de Soet JJ, Crielaard W (2010). The core genome of the anaerobic oral pathogenic bacterium Porphyromonas gingivalis. BMC microbiology.

[164] Klein BA, Tenorio EL, Lazinski DW, Camilli A, Duncan MJ, LT H (2012). Identification of essential genes of the periodontal pathogen Porphyromonas gingivalis. BMC genomics.

[165] Hutcherson JA, Gogeneni H, Yoder-Himes D, Hendrickson EL, Hackett M, Whiteley M, Lamont RJ, Scott DA (2016). Comparison of inherently essential genes of Porphyromonas gingivalis identified in two transposon-sequencing libraries. Molecular oral microbiology.

[166] Korona R (2011). Gene dispensability. Current opinion in biotechnology.

[167] Ijaq J, Chandrasekharan M, Poddar R, Bethi N, Sundararajan VS (2015). Annotation and curation of uncharacterized proteins- challenges. Frontiers in genetics.

[168] Fischer D, Eisenberg D: Finding families for genomic ORFans. Bioinformatics (Oxford, England) 1999, 15(9):759-762. 10.1093/bioinformatics/15.9.759 10498776

[169] Watanabe T, Maruyama F, Nozawa T, Aoki A, Okano S, Shibata Y, Oshima K, Kurokawa K, Hattori M, Nakagawa I (2011). Complete genome sequence of the bacterium Porphyromonas gingivalis TDC60, which causes periodontal disease. Journal of bacteriology.

[170] Tribble GD, Kerr JE, Wang BY (2013). Genetic diversity in the oral pathogen Porphyromonas gingivalis: molecular mechanisms and biological consequences. Future microbiology.

[171] Kerr JE, Abramian JR, Dao DH, Rigney TW, Fritz J, Pham T, Gay I, Parthasarathy K, Wang BY, Zhang W (2014). Genetic exchange of fimbrial alleles exemplifies the adaptive virulence strategy of Porphyromonas gingivalis. PloS one.

[172] Lamont RJ, Jenkinson HF (1998). Life below the gum line: pathogenic mechanisms of Porphyromonas gingivalis. Microbiology and molecular biology reviews : MMBR.

[173] How KY, Song KP, Chan KG (2016). Porphyromonas gingivalis: An Overview of Periodontopathic Pathogen below the Gum Line. Frontiers in microbiology.

[174] Nelson KE, Fleischmann RD, DeBoy RT, Paulsen IT, Fouts DE, Eisen JA, Daugherty SC, Dodson RJ, Durkin AS, Gwinn M (2003). Complete genome sequence of the oral pathogenic Bacterium porphyromonas gingivalis strain W83. Journal of bacteriology.

[175] Koren S, Phillippy AM (2015). One chromosome, one contig: complete microbial genomes from long-read sequencing and assembly. Current opinion in microbiology.

[176] Teng JLL, Yeung ML, Chan E, Jia L, Lin CH, Huang Y, Tse H, Wong SSY, Sham PC, Lau SKP (2017). PacBio But Not Illumina Technology Can Achieve Fast, Accurate and Complete Closure of the High GC, Complex Burkholderia pseudomallei Two-Chromosome Genome. Frontiers in microbiology.

[177] Craddock T, Harwood CR, Hallinan J, Wipat A (2008). e-Science: relieving bottlenecks in large-scale genome analyses. Nature reviews. Microbiology.

