# Supplementary figures and images for: Genomic repeats, misassembly and reannotation: a case study with long-read resequencing of Porphyromonas gingivalis reference strains
Source: BMC Genomics. 2018 Jan 16;19:54. doi: 10.1186/s12864-017-4429-4 (PMC5771137; doi:10.1186/s12864-017-4429-4)

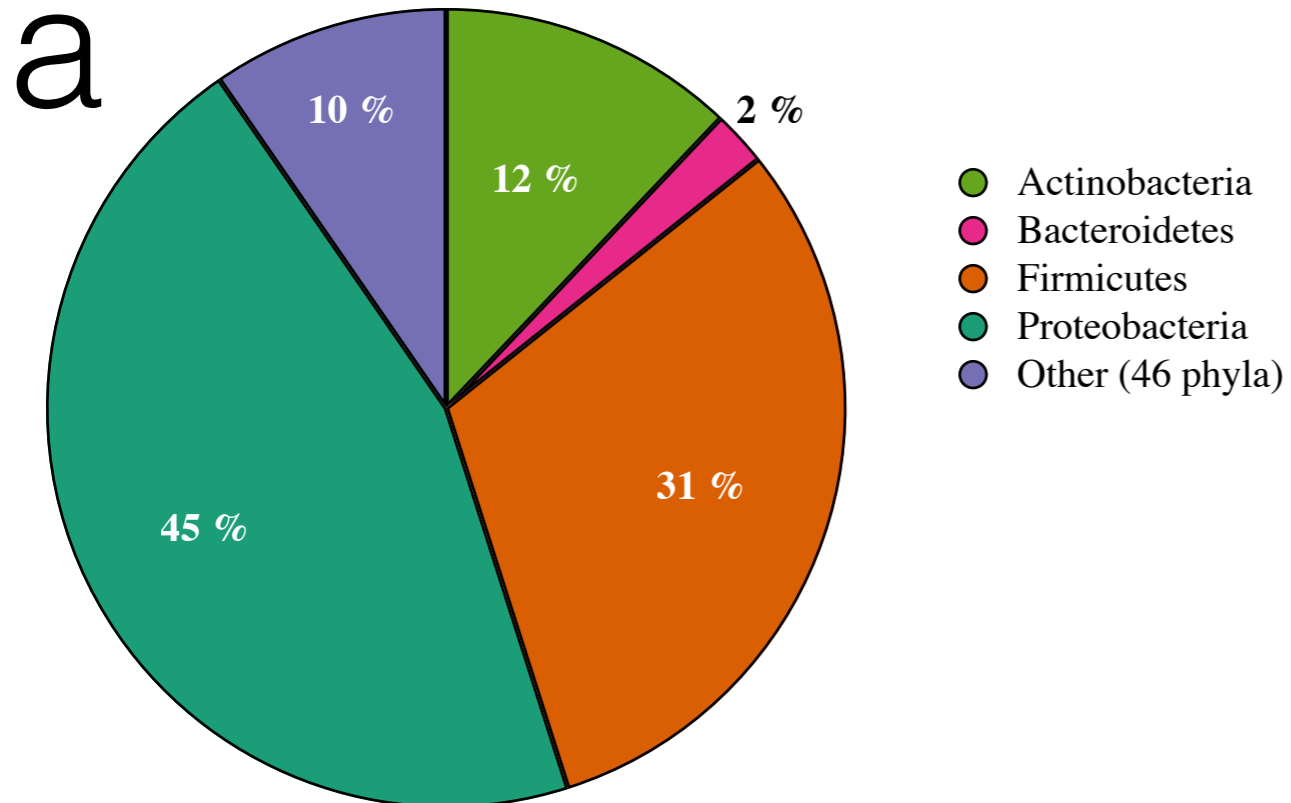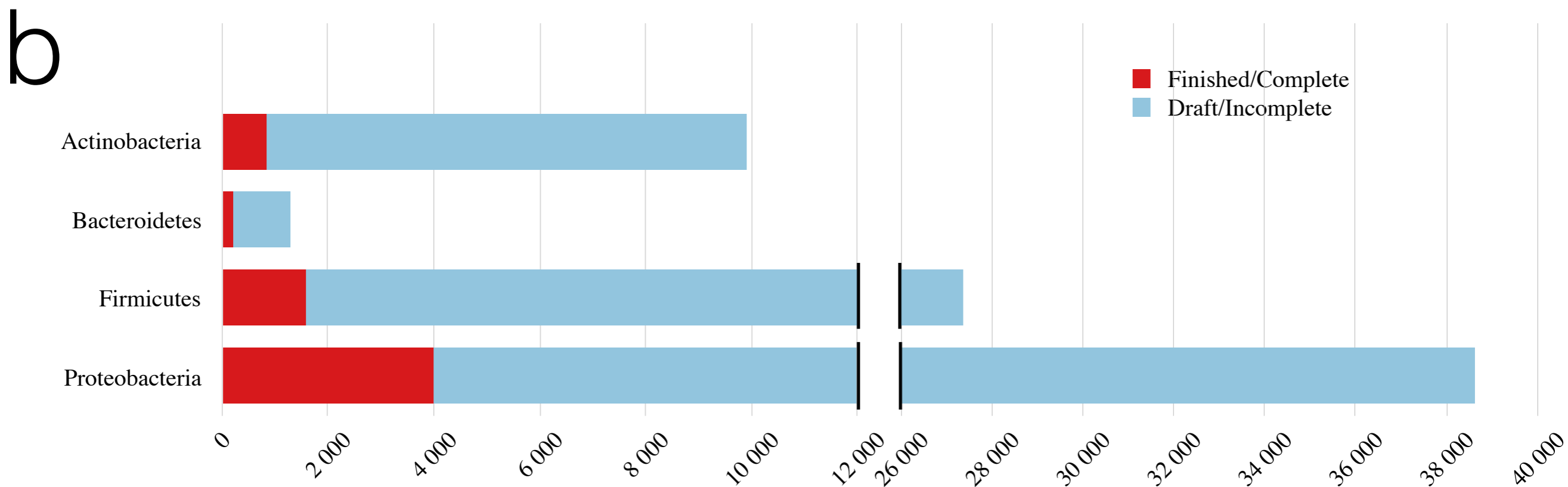

Supplement: Supplementary file 1 — Complete genomes not included in this study. When available, the species, strain, sequencing technology, assembler, coverage, Pubmed ID, release date, exclusion reason, and FTP link are presented for each genome. When no publication was found, genomes were marked as “unpublished,” and the sequencing organism was identified. Key: BCoM, Baylor College of Medicine; BU, Bielefeld University; DOE-JGI, United States Department of Energy-Joint Genome Institute; FI, The Forsyth Institute; HMP, Human Microbiome Project; JCVI, J. Craig Venter Institute; LIAEPB, Leibniz Institute for Agricultural Engineering Potsdam-Bornim; SI, Sanger Institute; and UoB, University of Bern. (PDF 43 kb) [file 12864_2017_4429_MOESM1_ESM.pdf]

a

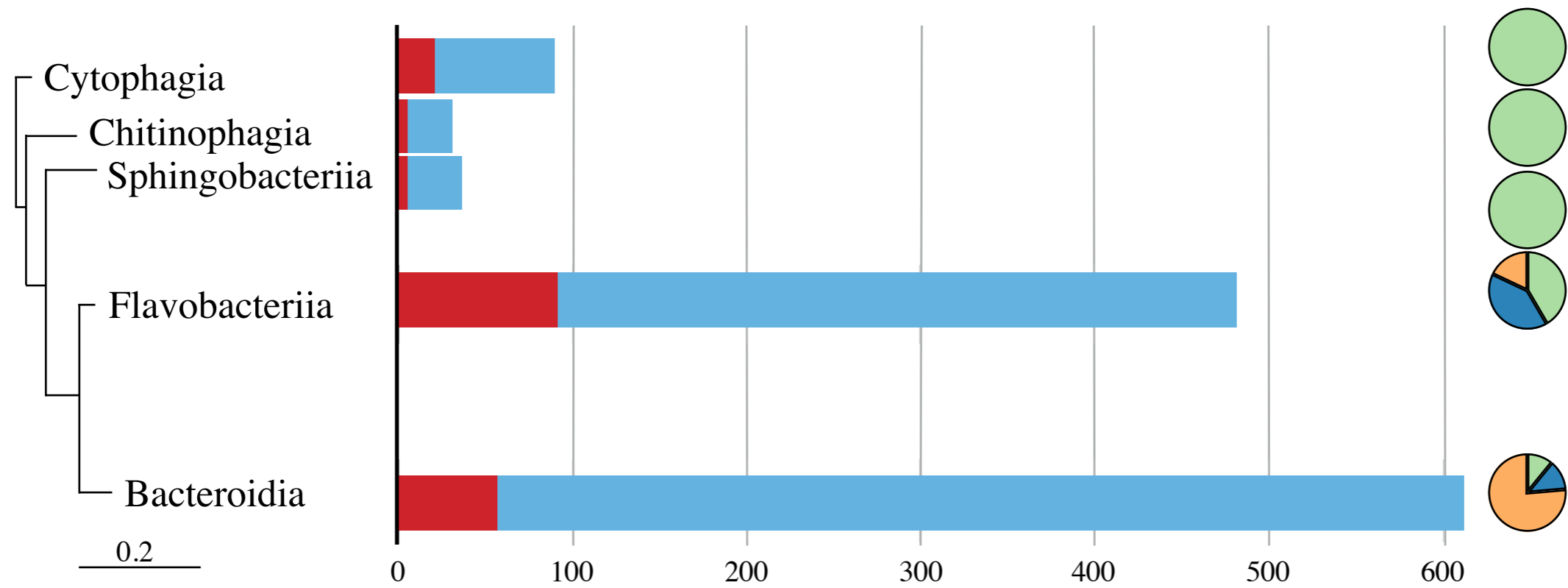

b

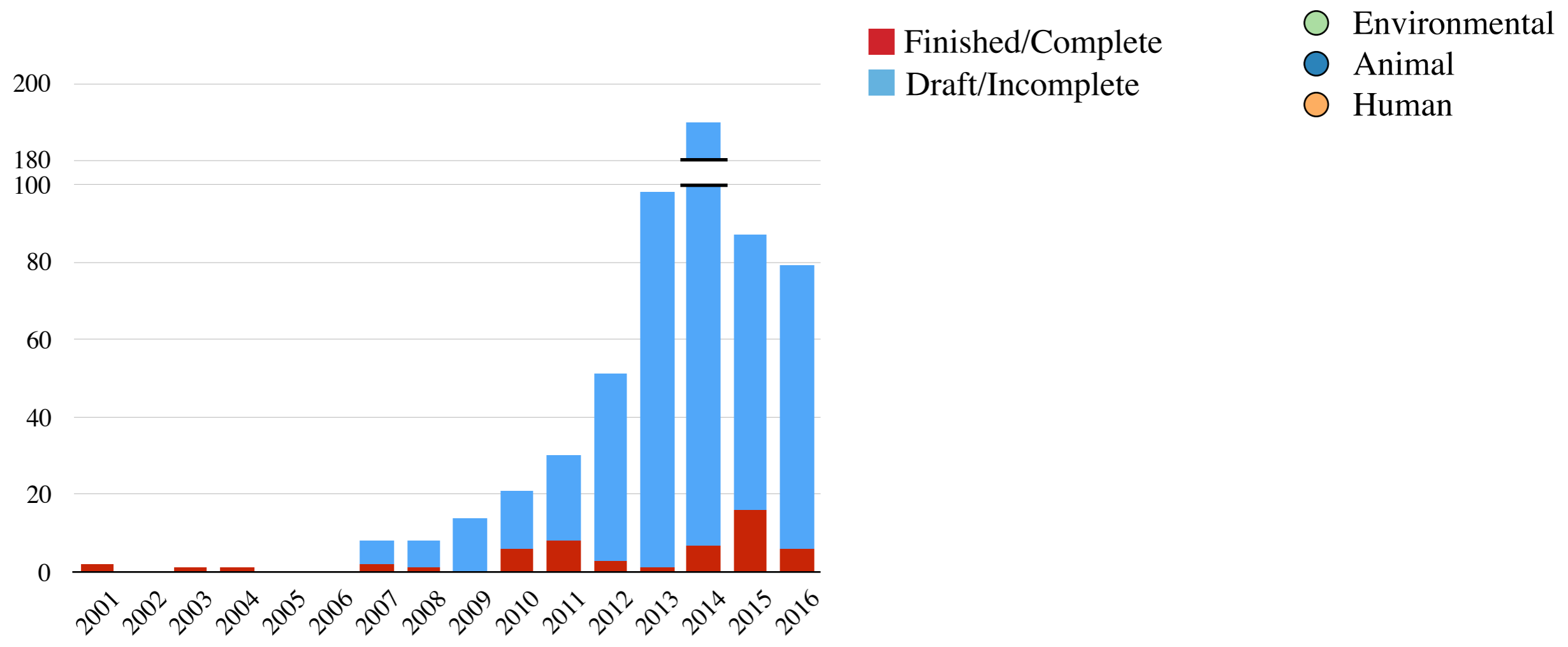

Supplement: Supplementary file 2 — NCBI genome database distribution of the main bacterial phyla associated with humans. a. Pie chart featuring the genomes present in the database, by phylum. b. Stacked bar chart of the incidence of the genomes belonging to the four main phyla associated with humans. Absolute counts are presented by phylum and classified as either finished/complete (having at least one chromosome and/or plasmid), or draft/incomplete (having multiple contigs or scaffolds). (PDF 61 kb) [file 12864_2017_4429_MOESM2_ESM.pdf]

a

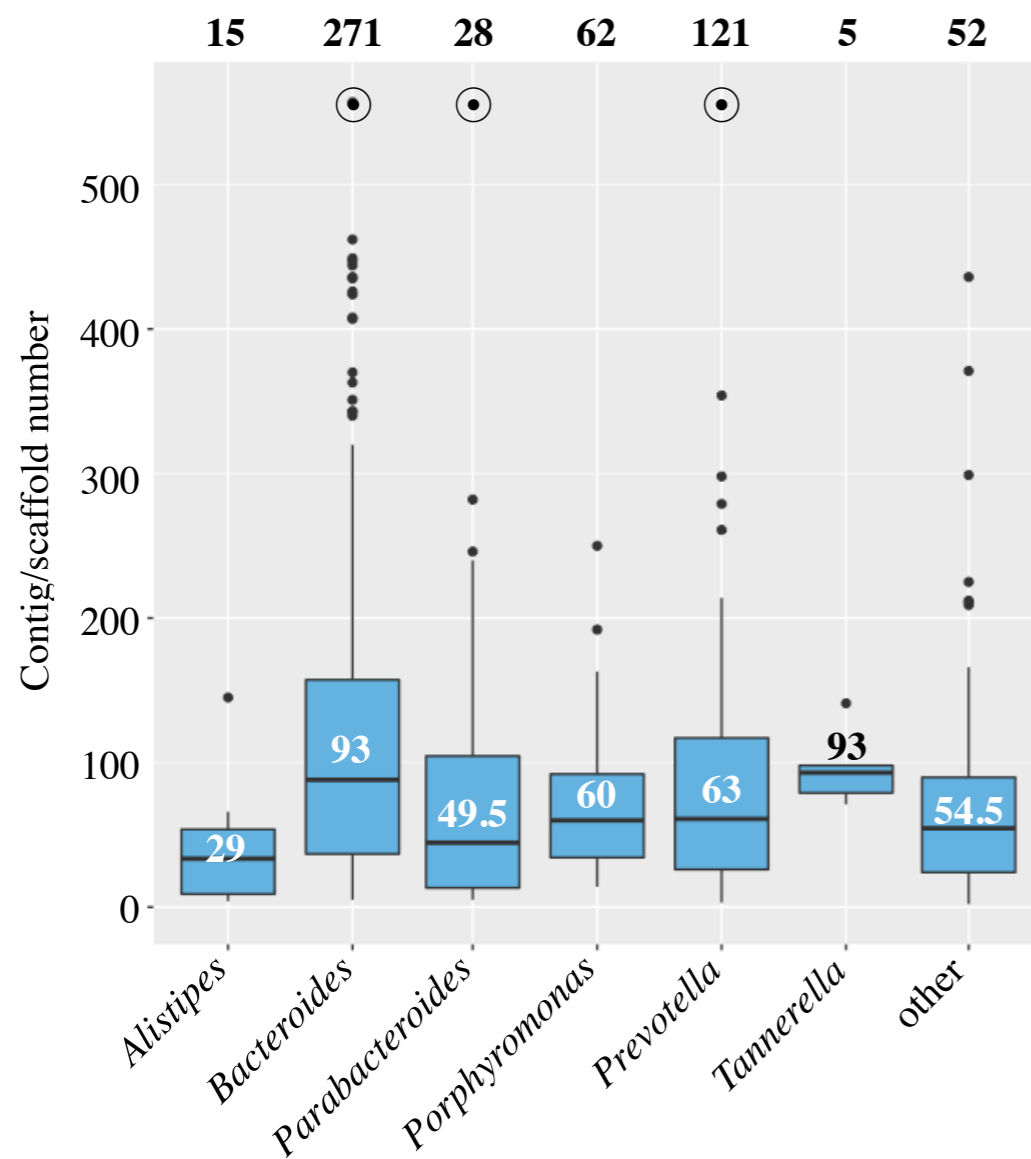

b

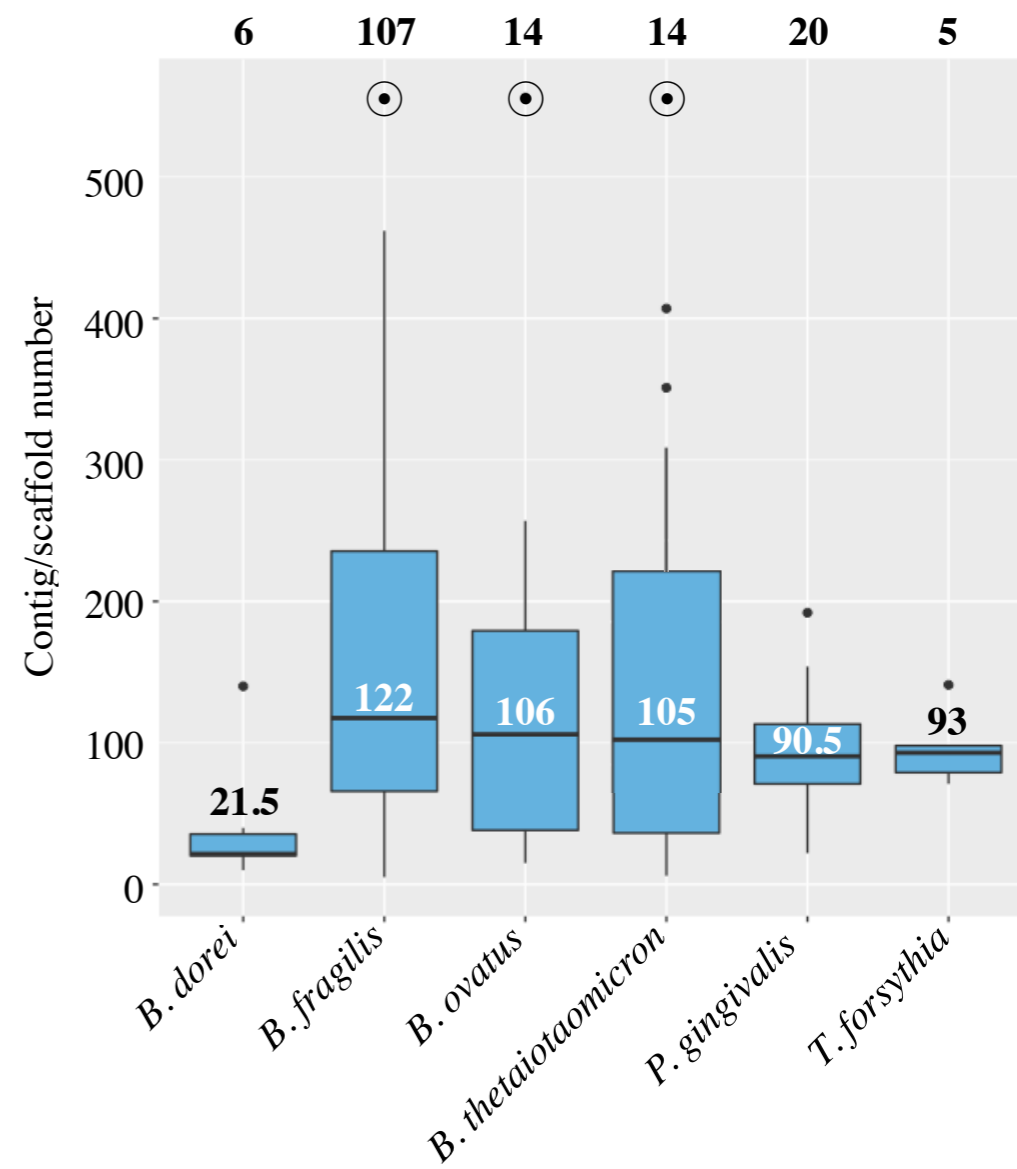

Supplement: Supplementary file 3 — Bacteroidetes genomes by class. a. On the left, a phylogenetic tree based on the 16S rRNA genes of complete genomes, grouped by class. A stacked bar chart then shows the number of genomes belonging to each Bacteroidetes class. The absolute genome counts are given, and classified as being either finished/complete or draft/incomplete (having multiple contigs or scaffolds). Pie charts on the right indicate the isolation sources for each genome: environmental (soil, fresh or marine water, and plants), animal (insects, molluscs, fish, birds, and mammals), or human (different body sites and health conditions). b. Stacked bar chart of Bacteroidia genomes grouped by status (complete or draft), presented by their publication year. (PDF 95 kb) [file 12864_2017_4429_MOESM3_ESM.pdf]

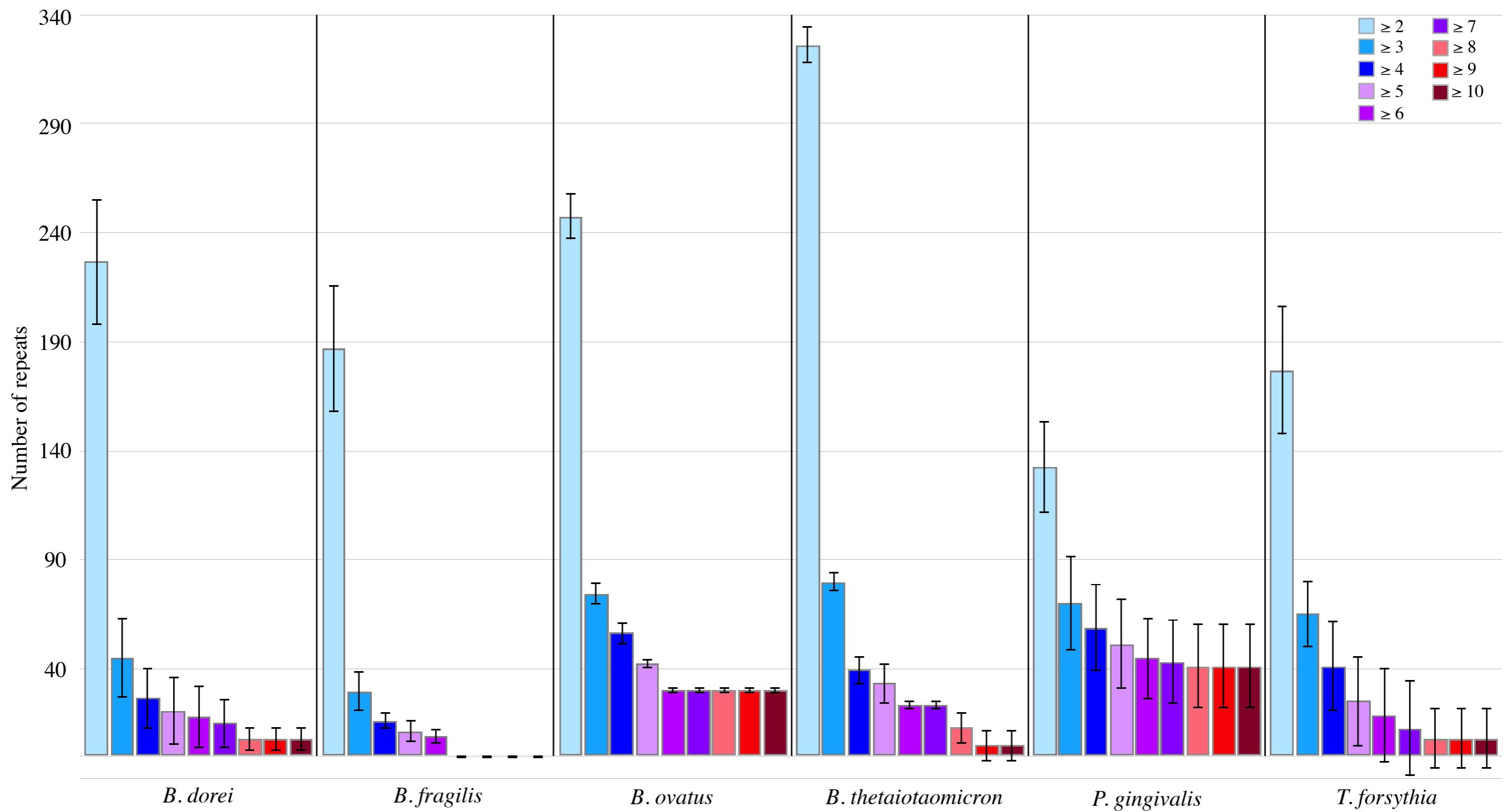

Supplement: Supplementary file 4 — Bacteroidia draft genomes binned by genus and by species. a. Box plot of draft/incomplete Bacteroidia genomes grouped by genus. With the exception of Tannerella which has complete genomes, any genus with less than 10 draft genomes was classified as “other.” The number of assemblies is presented above the plot, and the median is shown for each box. If a genus has drafts with more than 500 contigs/scaffolds, it is marked with ◉: Bacteroides (n = 17, 557 to 4357 contigs); Parabacteroides (n = 2, 1471 and 1920 contigs); and Prevotella (n = 3, 553 to 3171 contigs). b. Box plot of draft/incomplete Bacteroidia genomes for which at least two complete genomes exist, grouped by species, as per a. The drafts which have over 500 contigs/scaffolds are Bacteroides fragilis (n = 7, 557 to 2566 contigs), B. ovatus (n = 1, 556 contigs), and B. thetaiotaomicron (n = 2, 1730 and 2372 contigs). (PDF 34 kb) [file 12864_2017_4429_MOESM4_ESM.pdf]

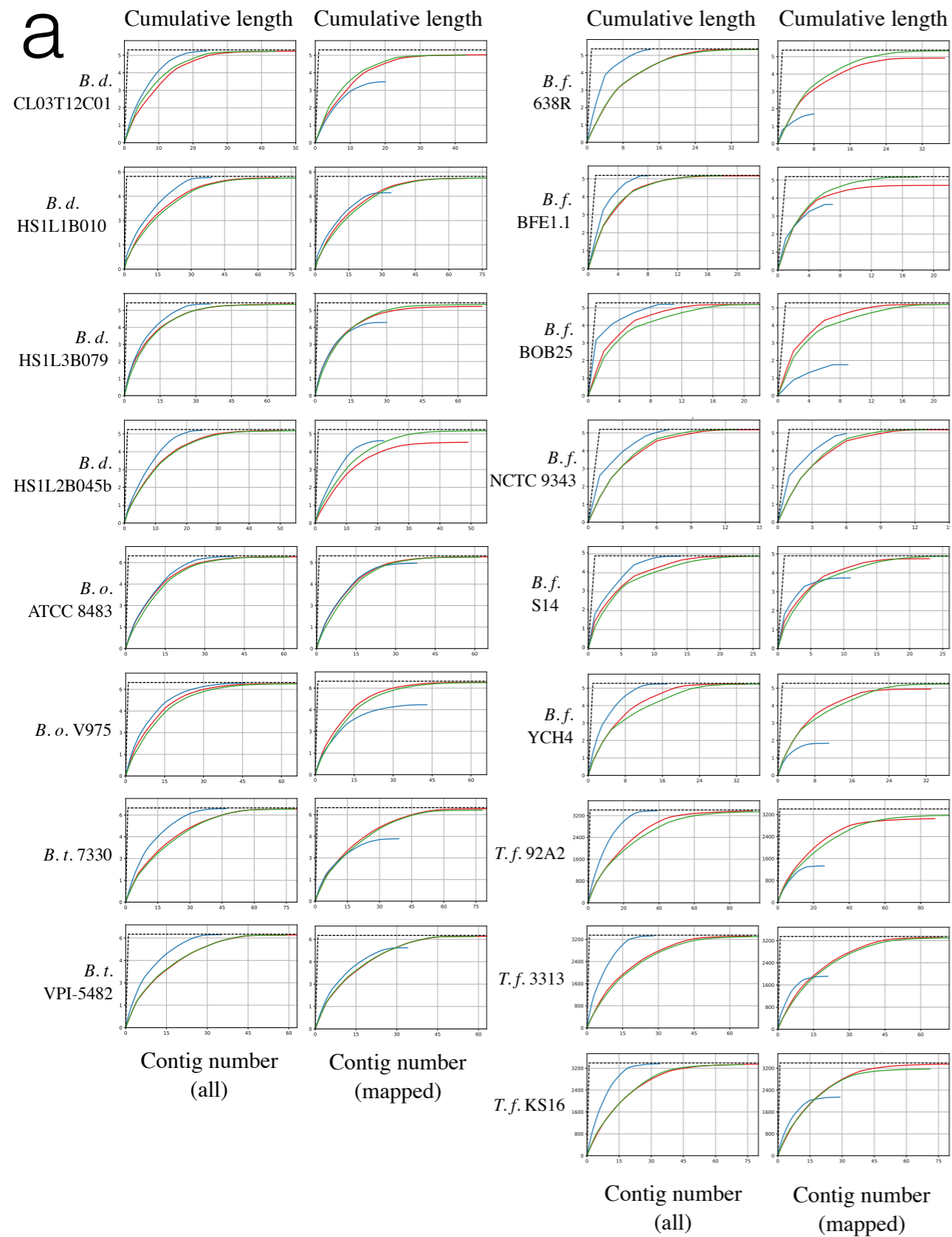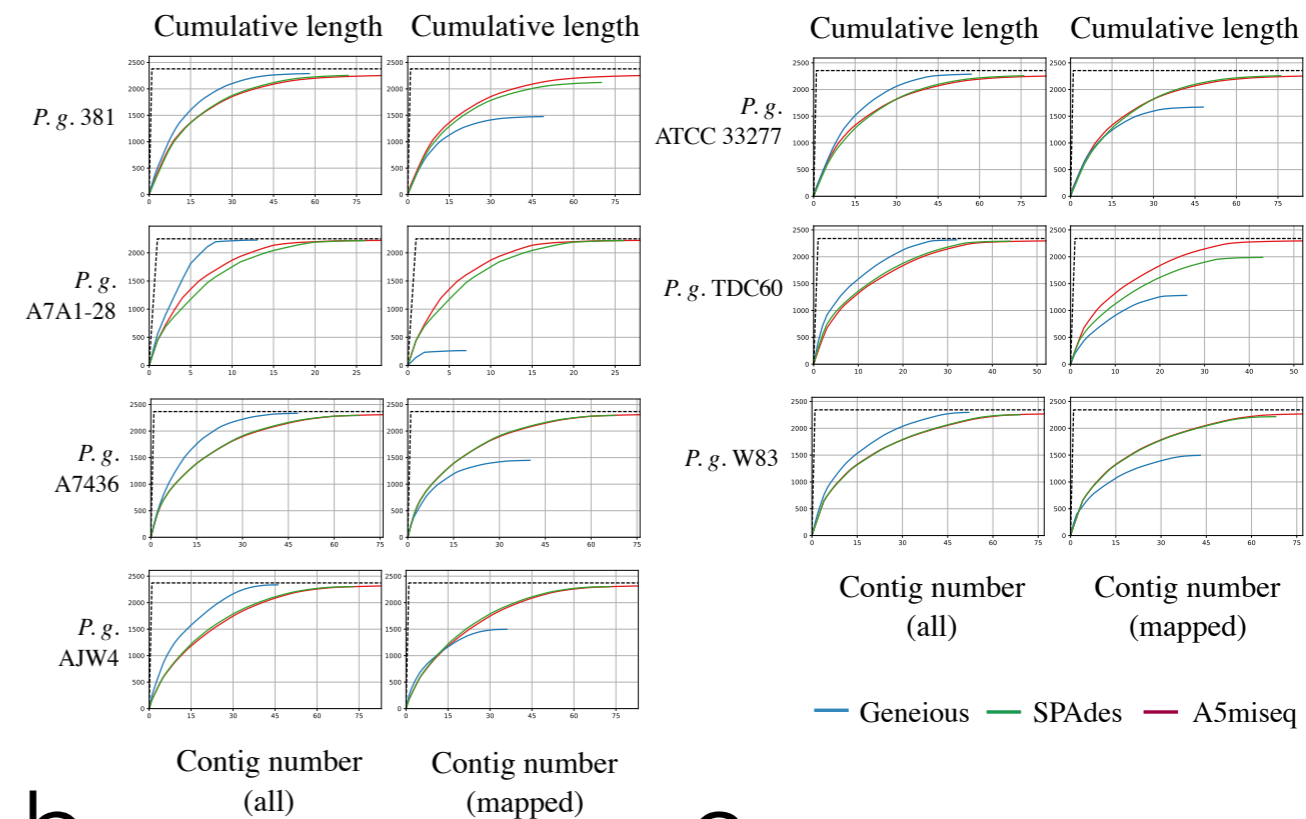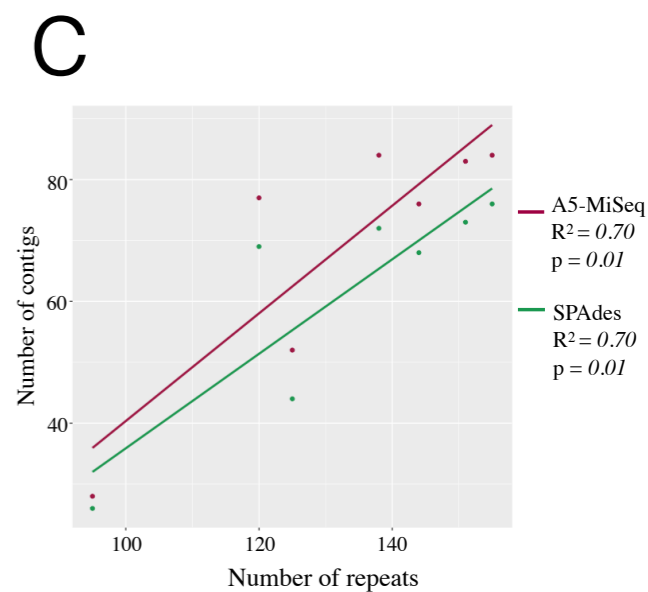

Supplement: Supplementary file 5 — The 166 draft genomes of the six Bacteroidia species studied here. Species, strain, sequencing technology, assembler, number of contigs, Pubmed ID, release date, and FTP links are presented. When no publication was found, genomes were marked as “unpublished” and the sequencing organism was identified. Key: *, no sequencing centre could be identified; BCoM, Baylor College of Medicine; BI, Broad Institute; DOE-JGI, United States Department of Energy-Joint Genome Institute; FMBA, Federal Medical-Biological Agency, Russia; HMP, Human Microbiome Project; IGS, Institute for Genome Science, University of Maryland; JCVI, J. Craig Venter Institute; TUD, Technical University of Denmark; UoS, University of Sheffield; and WU, Washington University. (PDF 1709 kb) [file 12864_2017_4429_MOESM5_ESM.pdf]

a

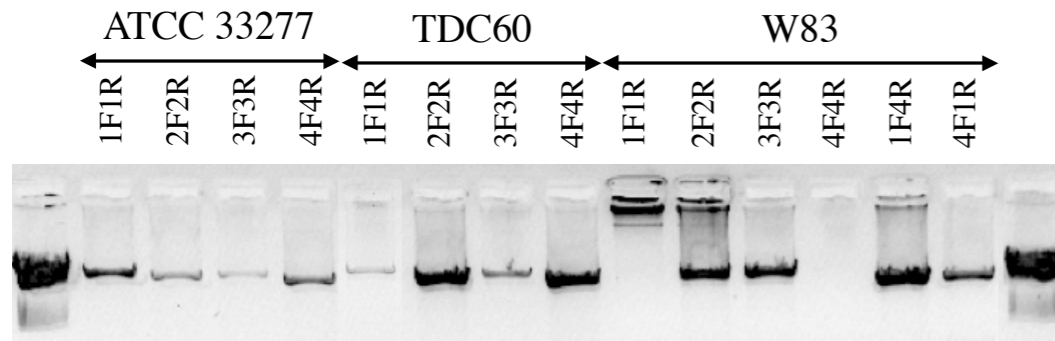

b

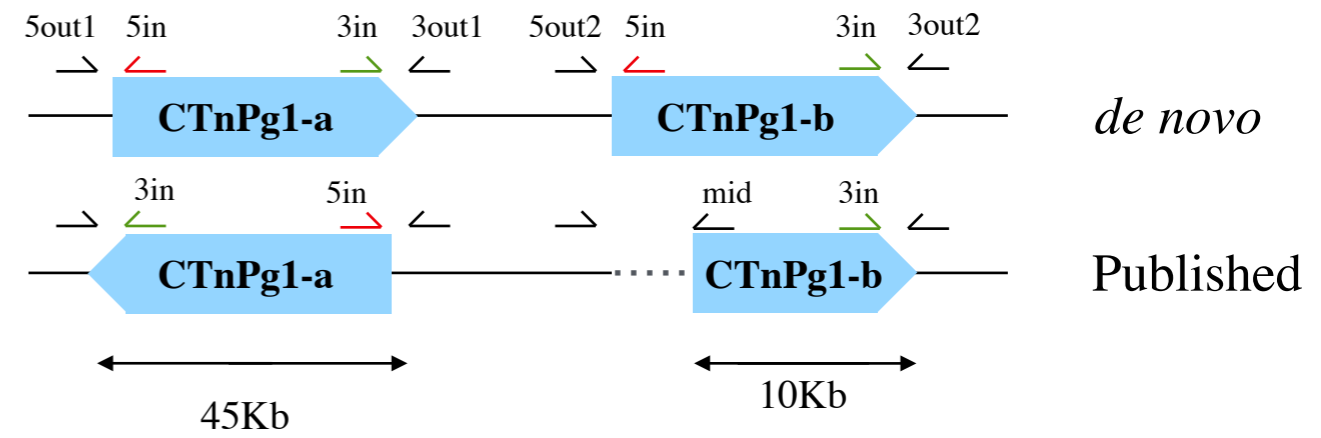

c

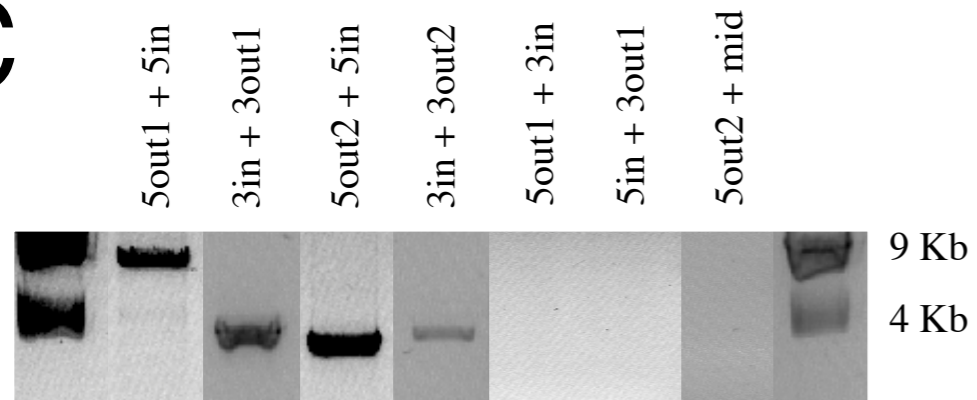

d

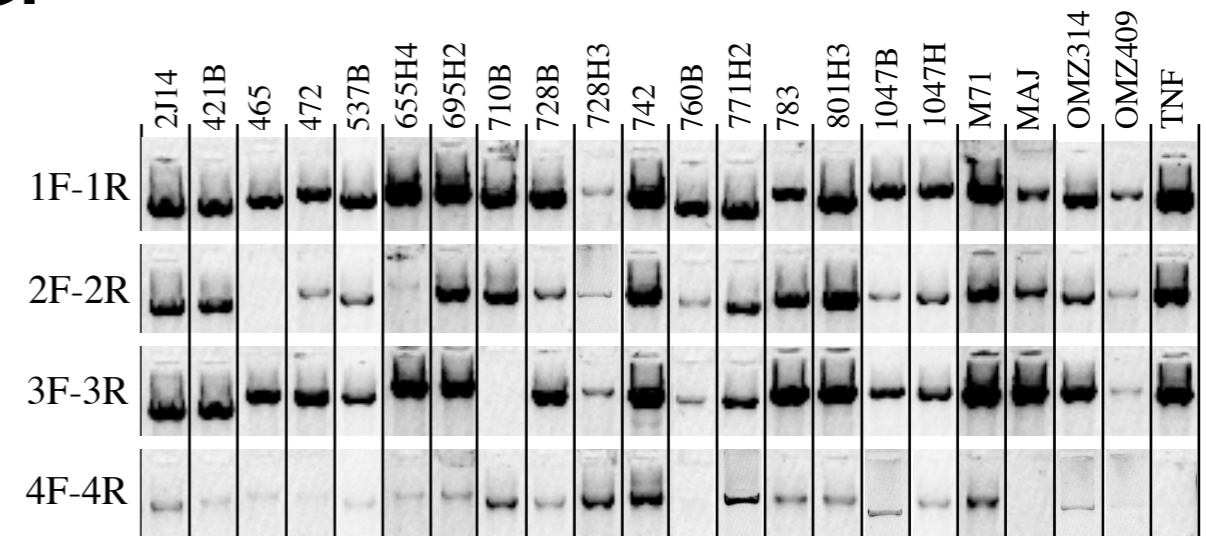

Supplement: Supplementary file 6 — Genomic repeats by species. Genomic repeats were identified for each genome, and the cumulative mean copy numbers and their standard deviations are presented. The light blue bar indicates the total number of repeats (at least 2 copies). (PDF 649 kb) [file 12864_2017_4429_MOESM6_ESM.pdf]

## P.g. ATCC 33277

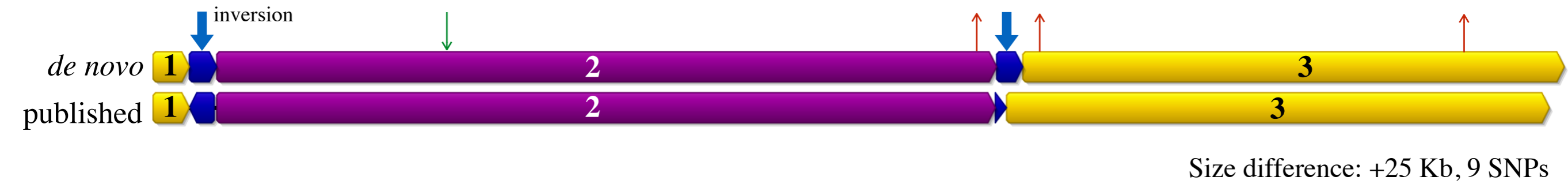

## P.g. TDC60

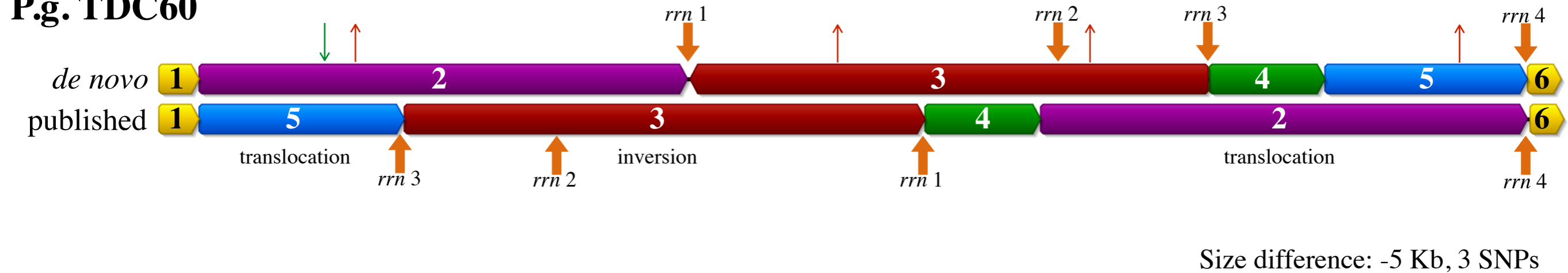

## P.g. W83

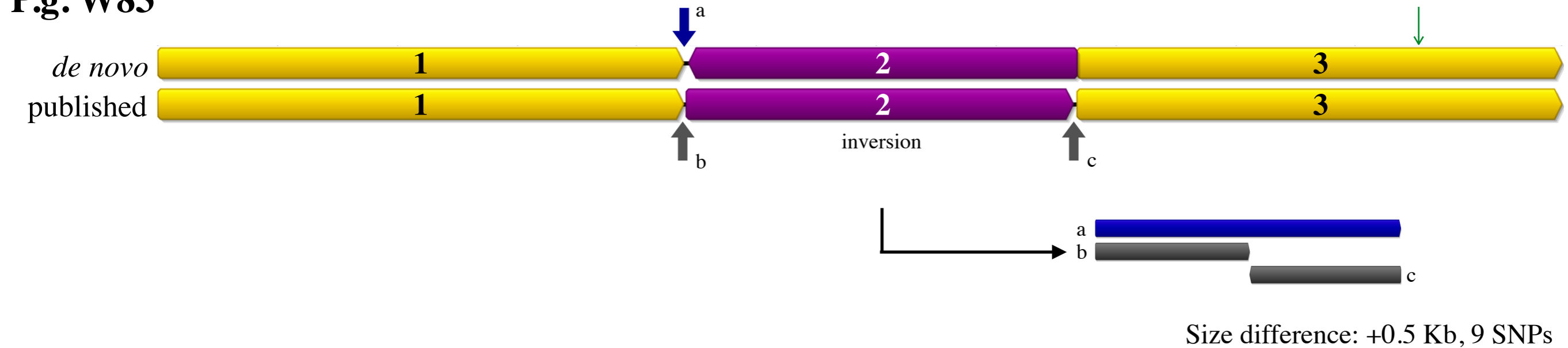

Supplement: Supplementary file 7 — De novo assembly of artificial reads of the studied Bacteroidia genomes. a. QUAST graph (cumulative length versus config index) for each assembly of each strain. The left column shows all contigs (> 1 Kbp), while the right shows only the contigs that mapped to its reference. The dotted line represents the reference genome size. b. For all 24 genomes, the contig counts from A5-miseq and SPAdes were plotted against the repeat counts (with at least 3 copies). c. As b, but showing all seven P. gingivalis strains. (PDF 133 kb) [file 12864_2017_4429_MOESM7_ESM.pdf]

# a

## *P. g.* ATCC 33277

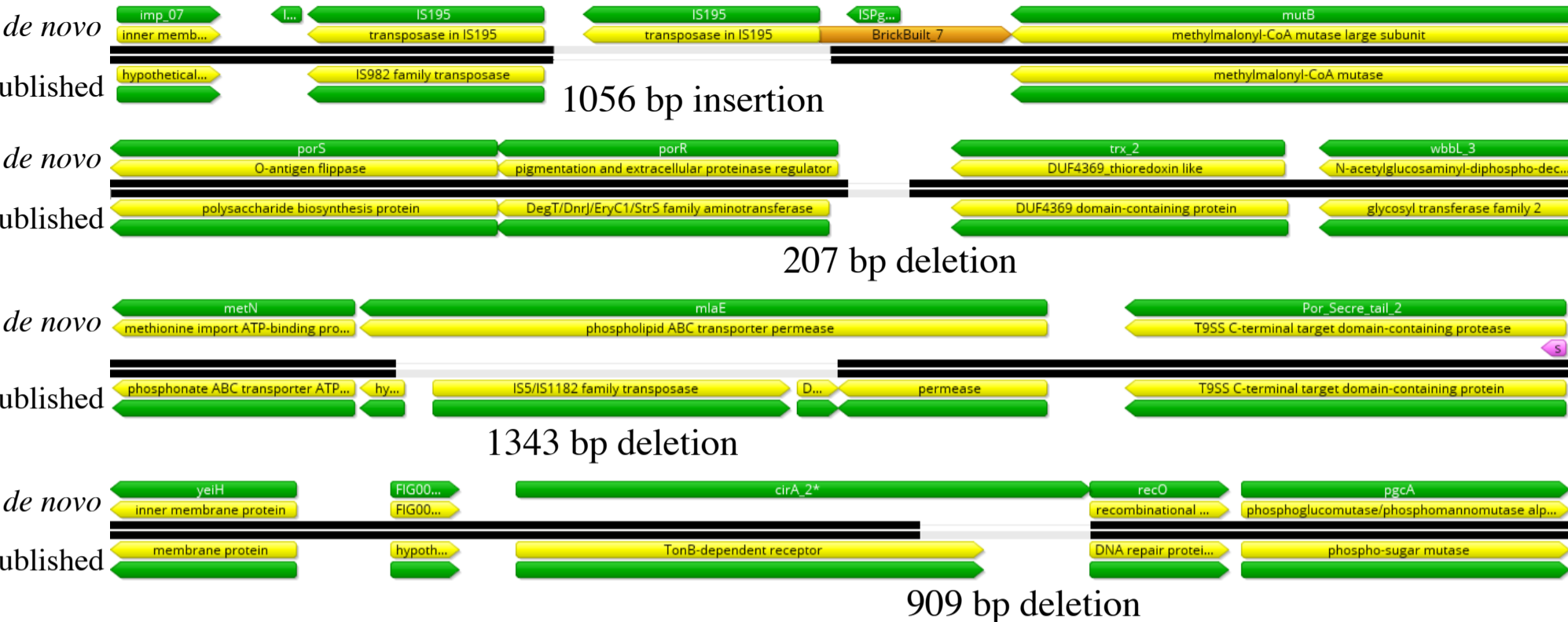

# b

## *P. g.* TDC60

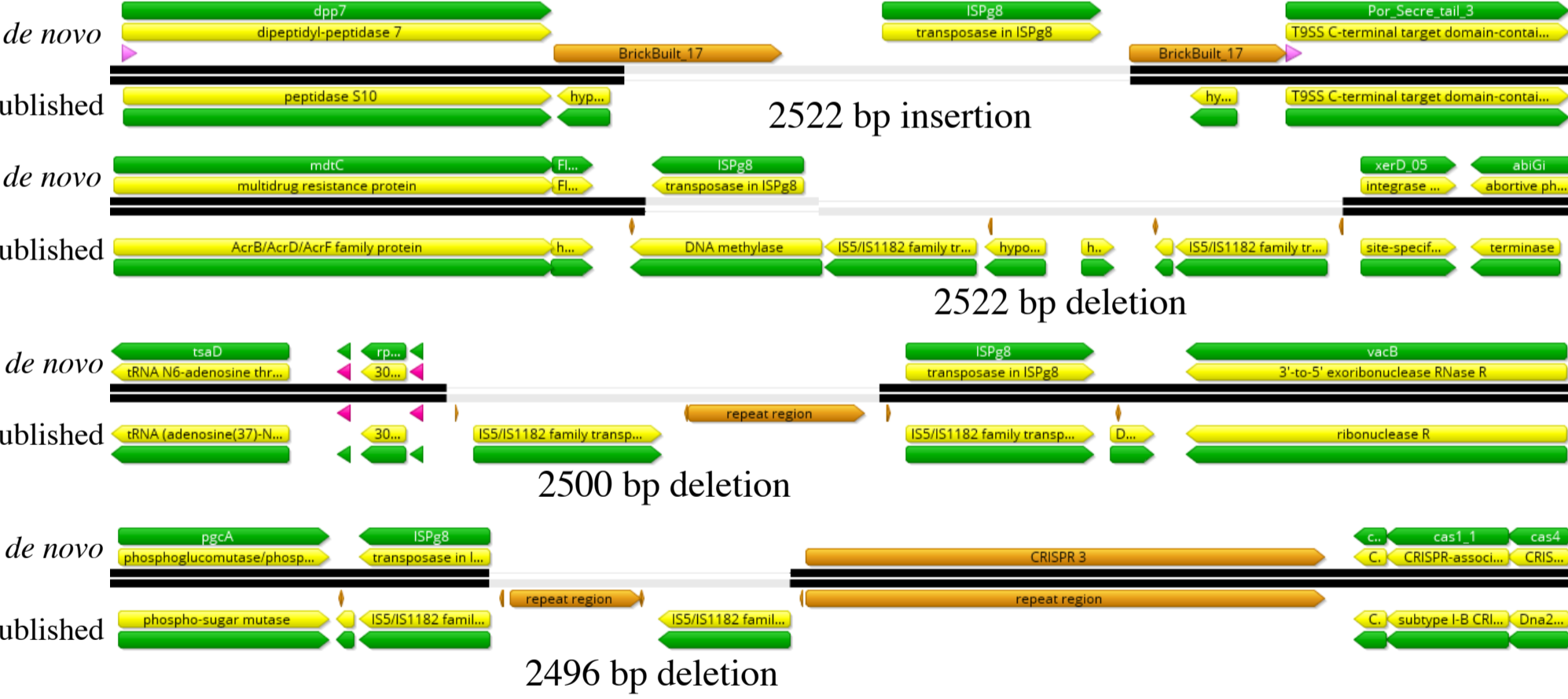

# c

## *P. g.* W83

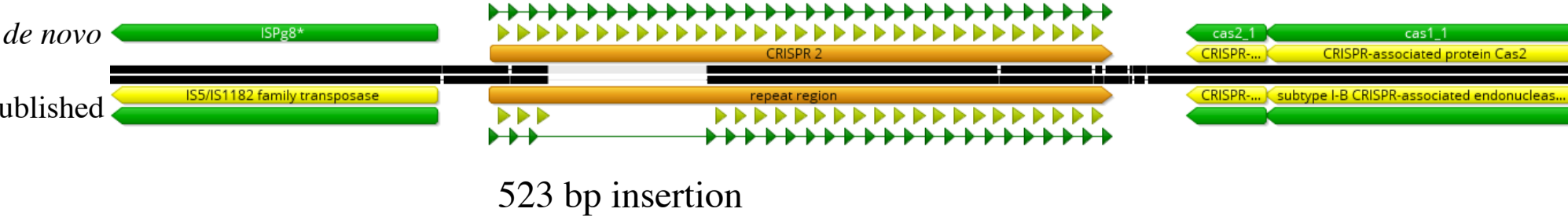

Supplement: Supplementary file 8 — PCR validation of 3 P. gingivalis strain constructions. a. Agarose gel electrophoresis (0.8% in 1X TBE buffer, stained with 1X GelRed) of PCR products for rrn operons. Primers used and verified strains are indicated by lane, and the primer names were simplified (“rrn” is not mentioned). DNA molecular-weight size markers were used in the first and last lanes. The first band is 9 Kb, and the second is 4 Kb. b. Schematic representation (not to scale) of both copies (“a” and “b”) of CTnPg1 from P. gingivalis ATCC 33277. The de novo assembly identified two complete copies with the same orientation. The published ATCC 33277 strain had two copies, but the second was partial and inverted when compared to the first. Size and orientation are presented for clarity, and the dotted line indicates the absent CTnPg1-b region in the published genome. Primers names were simplified (“ctnpg1_” is not mentioned). The ctnpg1_5in is red, the ctnpg1_3in is green, and all other primers are black. c. Agarose gel electrophoresis done as in a. for the primer combination indicated over each lane. In the order shown, the expected sizes are 7.5, 3.5, 3.0, 3.5, 5.0, 6.5, and 10 Kb. d. Agarose gel electrophoresis as above for rrn operons in 22 P. gingivalis strains. For PCR conditions, primer sequences, and strain origins, see Methods. (PDF 194 kb) [file 12864_2017_4429_MOESM8_ESM.pdf]
