# Supplementary material for: Genomic repeats, misassembly and reannotation: a case study with long-read resequencing of Porphyromonas gingivalis reference strains
Source: BMC Genomics. 2018 Jan 16;19:54. doi: 10.1186/s12864-017-4429-4 (PMC5771137; doi:10.1186/s12864-017-4429-4)
Supplement: Supplementary file 9 — Whole-genome alignments of the three resequenced P. gingivalis strains. Published and de novo assembled genome architectures are compared. Locally collinear blocks (LCBs) were detected using the progressiveMauve algorithm. Shown are translocations and inversions, insertions (green arrows), and deletions (thin red arrows), along with size differences and SNP counts. For ATCC 33277, the blue blocks represent CTnPg1 copies; in TDC60, they are the rrn operons. For W83, the de novo sequence “a” was assembled from the published genome’s “b” and “c” sequences (see text for details). (PDF 27 kb) [file 12864_2017_4429_MOESM9_ESM.pdf]

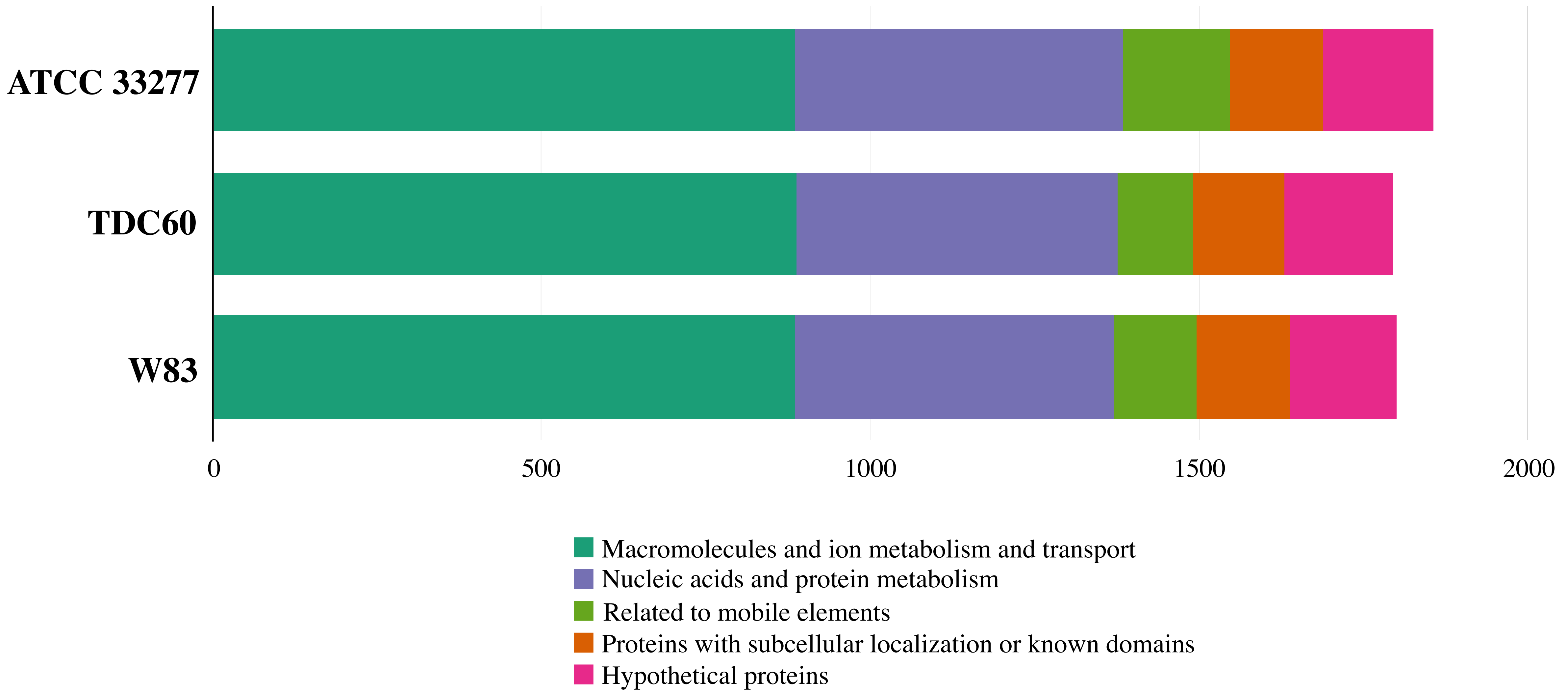
ATCC 33277
TDC60
W83
0
500
1000
1500
2000
Macromolecules and ion metabolism and transport
Nucleic acids and protein metabolism
Related to mobile elements
Proteins with subcellular localization or known domains
Hypothetical proteins
