# Supplementary material for: Genomic repeats, misassembly and reannotation: a case study with long-read resequencing of Porphyromonas gingivalis reference strains
Source: BMC Genomics. 2018 Jan 16;19:54. doi: 10.1186/s12864-017-4429-4 (PMC5771137; doi:10.1186/s12864-017-4429-4)
Supplement: Supplementary file 10 — Insertions and deletions in the de novo P. gingivalis assemblies as compared to the published genomes. In all cases, alignment zooms are presented for the corresponding insertions or deletions detailed in Fig. S8 and in the text. The upper genome is the de novo assembly and the bottom is the published one. a. The ATCC 33277 strain has four indels. b. TDC has four indels, with an additional 22-bp deletion in an intergenic region (not depicted). c. W83 has a single insertion. (PDF 65 kb) [file 12864_2017_4429_MOESM10_ESM.pdf]

a

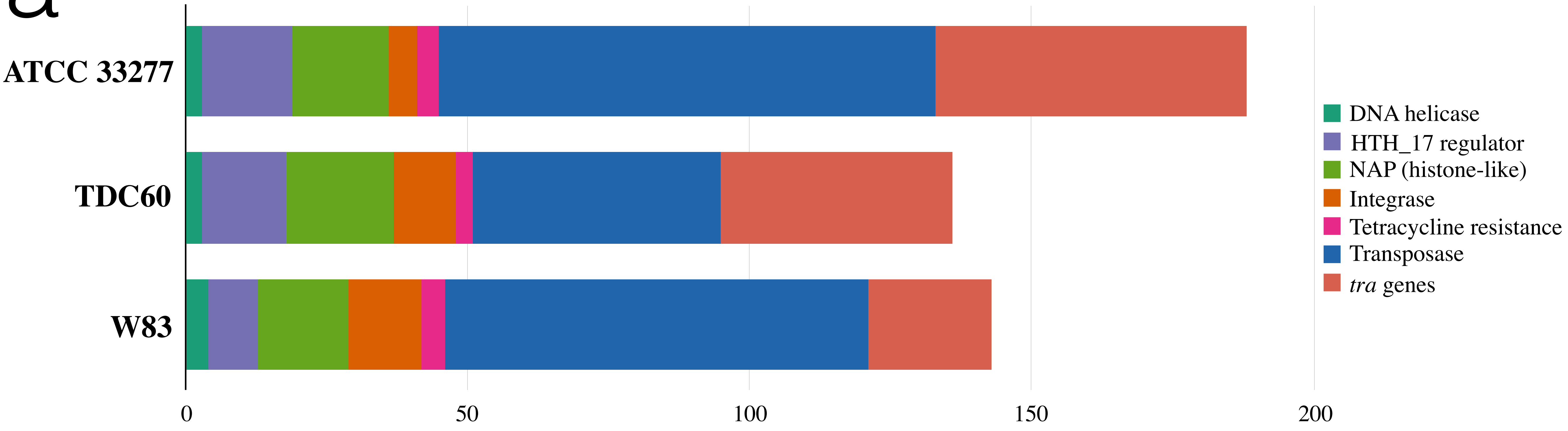

b

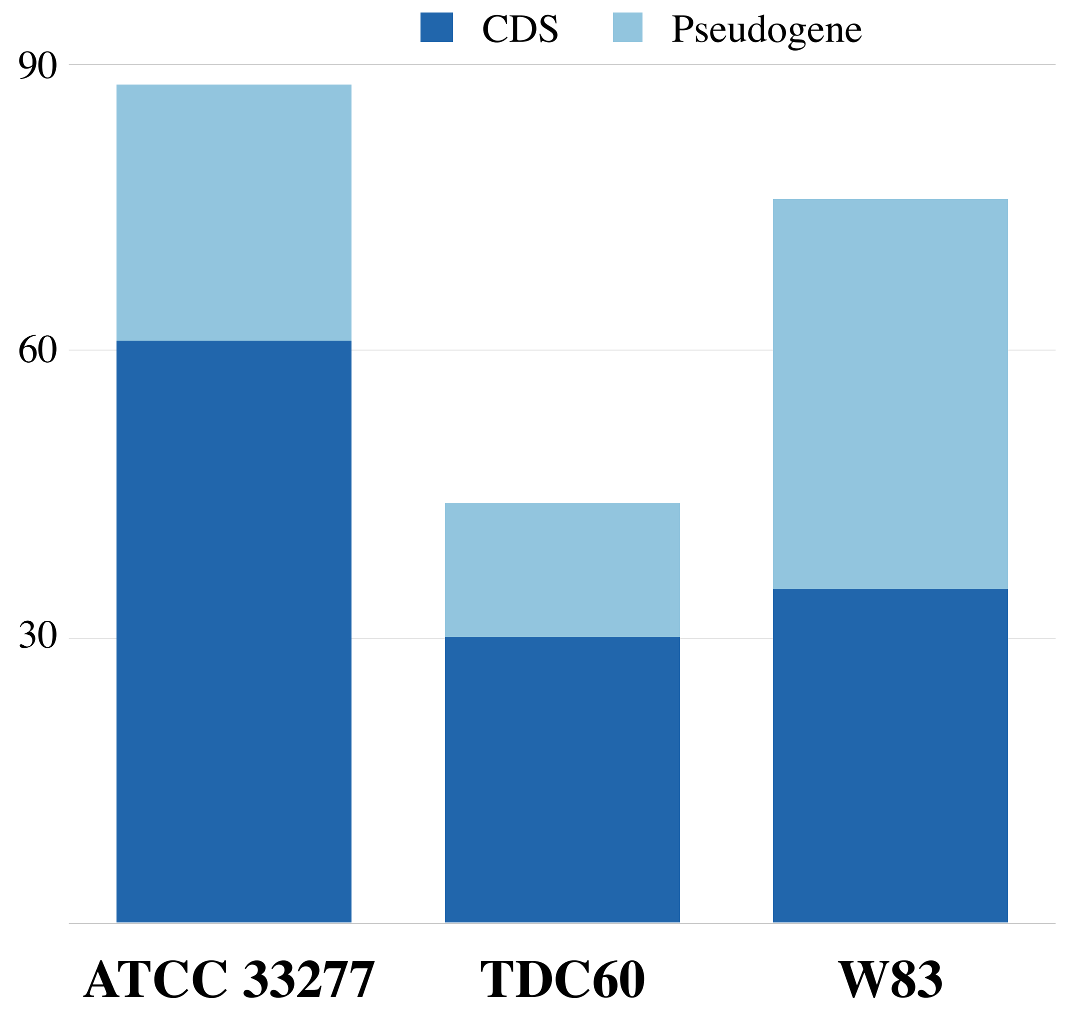

c

	ATCC 33277		TDC60		W83	
	CDS	pseudogene	CDS	pseudogene	CDS	pseudogene
**ISPg8**	32	10	16	5	11	22
**ISPg2**	3	4	1	2	5	3
**IS195**	24	4	10	1	5	3
**ISPg4**	0	1	0	1	10	1
**ISPg5**	0	5	0	0	1	11
**ISPg6**	0	3	1	2	1	0
**ISPg7**	0	0	1	0	1	0
**IS256**	0	0	0	2	0	1
**IS4**	0	0	1	1	1	0
**ISL3**	2	0	0	0	0	0
