# Supplementary material for: Genomic repeats, misassembly and reannotation: a case study with long-read resequencing of Porphyromonas gingivalis reference strains
Source: BMC Genomics. 2018 Jan 16;19:54. doi: 10.1186/s12864-017-4429-4 (PMC5771137; doi:10.1186/s12864-017-4429-4)
Supplement: Supplementary file 11 — Classification of additional CDS/pseudogenes. After a manual biocurated annotation, the changes were separated into five functional categories. The absolute counts of the new CDS/pseudogenes, CDS changed to pseudogenes or vice versa, feature fusions or splitting of single features into two, and coding strand changes are presented here, strain by strain. The results are emphasised via a heatmap that goes from lilac to burgundy. For re-annotation of the CDS/pseudogenes that are shared between the two versions, see Results and Fig. 4. (PDF 84 kb) [file 12864_2017_4429_MOESM11_ESM.pdf]

a

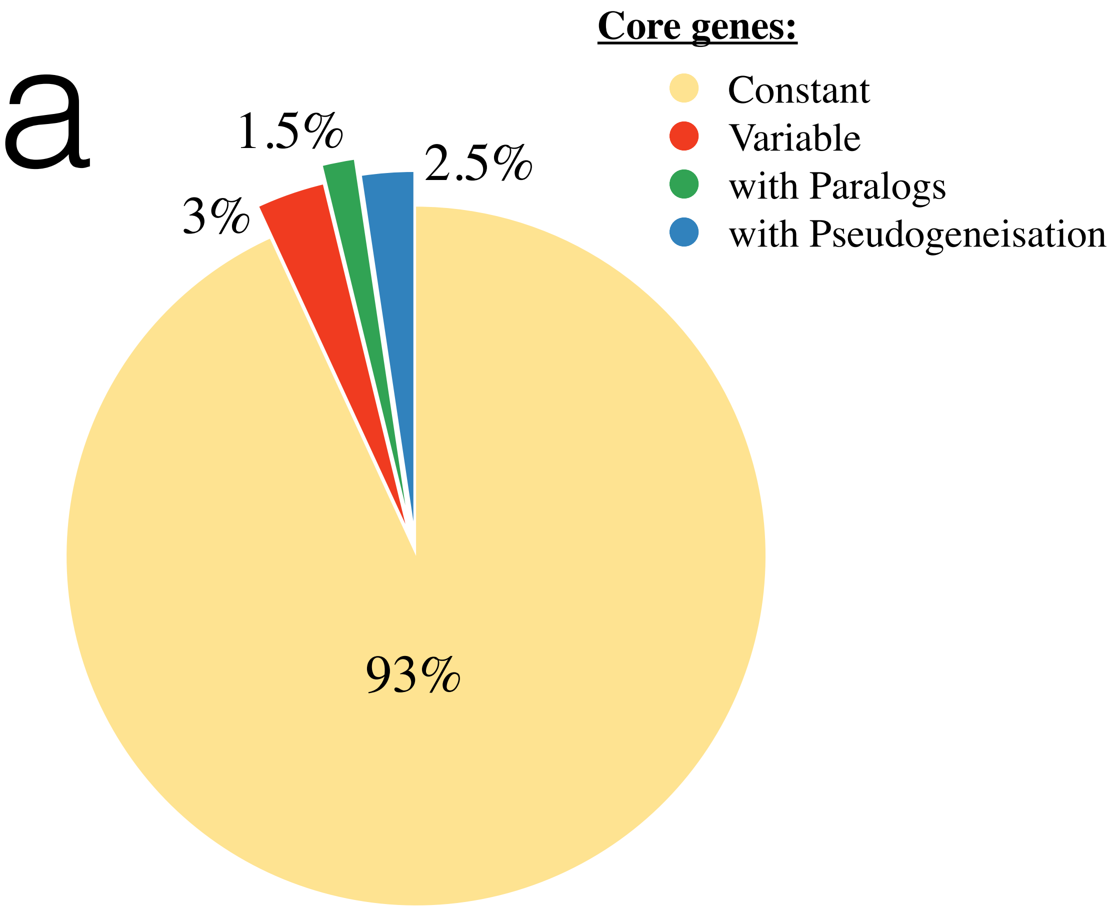

b

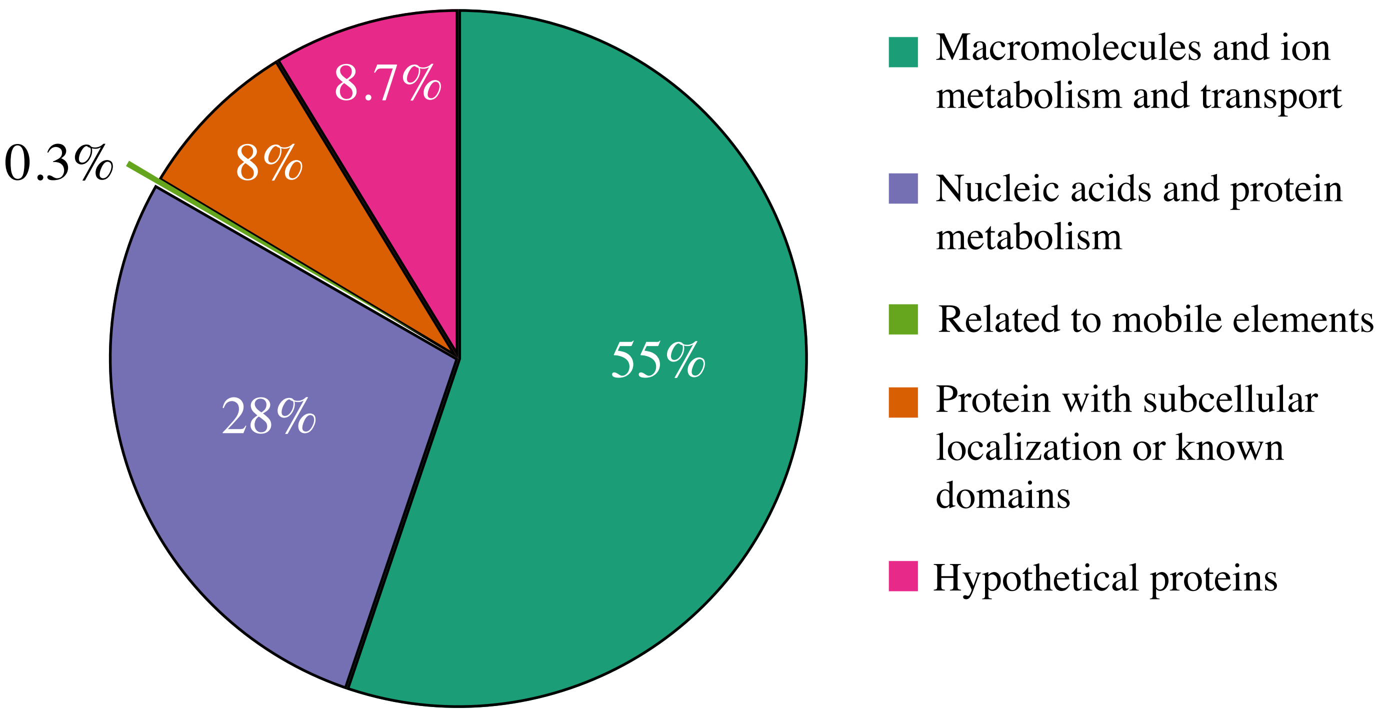

c

Function	Genes
CRISPR	*cas3*, *cas7*, *cmr2*, *cmr3*, *cmr5*, and *cmr6*
Fimbriae and pili	*fimA*, *fimC*, *fimD*, *fimE*, *mfa4;* and *ycf3_1*
Transport	*dtpT*, *ftsX_2*, *inlA*, *mepA_4*, *tonB_3*, and *wza*
Central metabolism	*capM*, *cysE*, *ghf*, *gppA*, *mfuc_2*, *mnmE*, *msuE*, *pheT_2*, *ragA*, *ragB*, *serB_2*, *topB3*, *wecG*, and *bmgA*
Type I restriction enzyme	*hsdR_1*
Haemagglutinin	*hagA*
Lysine gingipain protease	*kgp_4*
Unknown	*FIG00935998*, *FIG00936006*, *FIG00936766*, *FIG00936810*, *hypo_47*, *imp_35*, *imp_lipo_01*, *omp_lipo_1*, *omp_lipo_2*, *omp28*, and *yrrB_2*

d

gene	product	ATCC 33277	TDC60	W83
alkD	DNA alkylation repair enzyme	2	1	1
betI	transcriptional regulator	2	1	1
btr	transcriptional activator	2	1	2
ctpA	serine protease carboxy-terminal processing	2	1	1
DUF1896	hypothetical protein	2	2	1
epsJ	glycosyltransferase	2	2	1
era	GTPase	1 + 1*	1	1
FIG00935715	hypothetical protein	2	1	1
FIG00936125	hypothetical protein	2	1	1
hypo_01	hypothetical protein†	2	1	2
irtA	iron import ATP-binding/permease	2	1	1
ndvA	ABC transporter ATP-binding protein	2	1	1
rhuM	virulence protein RhuM family	2	1	2
ydfJ	exporter RND superfamily	2	1	1
ytxK	DNA methylase	2	2	1*
