# Supplementary material for: Genomic repeats, misassembly and reannotation: a case study with long-read resequencing of Porphyromonas gingivalis reference strains
Source: BMC Genomics. 2018 Jan 16;19:54. doi: 10.1186/s12864-017-4429-4 (PMC5771137; doi:10.1186/s12864-017-4429-4)
Supplement: Supplementary file 12 — The three P. gingivalis strains binned by their CDS/pseudogene functions. The coding sequences and pseudogenes were classified into five categories as shown, and the histogram is based on their absolute counts. (PDF 58 kb) [file 12864_2017_4429_MOESM12_ESM.pdf]

Species	Strain	Sequencing Technology	Assembler	Coverage	Reference Pubmed ID	Release Date	Exclusion reason	FTP
Alistipes finegoldii	DSM 17242	454 + Illumina	Newbler	30	Unpublished (DOE-JGI)	2012	Only 1 complete genome	ftp://ftp.ncbi.nlm.nih.gov/genomes/all/GCA/000/265/365/GCA_000265365.1_ASM26536v1
Alistipes shahii	WAL 8301	454	Unknown	22	Unpublished (SI)	2010	Only 1 complete genome	ftp://ftp.ncbi.nlm.nih.gov/genomes/all/GCA/000/210/575/GCA_000210575.1_ASM21057v1
Bacteroides caecimuris	I48	PacBio	HGAP + Celera	243	Unpublished (UoB)	2016	Only 1 complete genome	ftp://ftp.ncbi.nlm.nih.gov/genomes/all/GCA/001/688/725/GCA_001688725.1_ASM168872v1
Bacteroides cellulosilyticus	WH2	PacBio + Illumina	HGAP + Celera	209	26430127	2015	Only 1 complete genome	ftp://ftp.ncbi.nlm.nih.gov/genomes/all/GCA/001/318/345/GCA_001318345.1_ASM131834v1
Bacteroides coprosuis	DSM 18011	454 + Illumina	Newbler	30	21677860	2011	Only 1 complete genome	ftp://ftp.ncbi.nlm.nih.gov/genomes/all/GCA/000/212/915/GCA_000212915.1_ASM21291v1
Bacteroides helcogenes	P 36-108	454 + Illumina	Newbler	30	21475586	2011	Only 1 complete genome	ftp://ftp.ncbi.nlm.nih.gov/genomes/all/GCA/000/186/225/GCA_000186225.1_ASM18622v1
Bacteroides salanitronis	DSM 18170	454 + Illumina	Newbler	30	21677856	2011	Only 1 complete genome	ftp://ftp.ncbi.nlm.nih.gov/genomes/all/GCA/000/190/575/GCA_000190575.1_ASM19057v1
Bacteroides vulgatus	ATCC 8482	Sanger	Phrap + PCAP	13	17579514	2007	Only 1 complete genome	ftp://ftp.ncbi.nlm.nih.gov/genomes/all/GCA/000/012/825/GCA_000012825.1_ASM1282v1
Bacteroides vulgatus	mpk	PacBio	Celera	330	27071651	2015	Gap(s) in sequence	ftp://ftp.ncbi.nlm.nih.gov/genomes/all/GCA/001/412/315/GCA_001412315.1_ASM141231v1
Bacteroides xylanisolvens	XB1A	454	Unknown	18	Unpublished (SI)	2010	Only 1 complete genome	ftp://ftp.ncbi.nlm.nih.gov/genomes/all/GCA/000/210/075/GCA_000210075.1_ASM21007v1
Barnesiella viscericola	C46, DSM 18177	Unknown	ALLPATHS + Velvet + Phrap	Unknown	Unpublished (DOE-JGI)	2014	Only 1 complete genome	ftp://ftp.ncbi.nlm.nih.gov/genomes/all/GCA/000/512/915/GCA_000512915.1_ASM51291v1
Draconibacterium orientale	FH5	Sanger + 454 + Illumina	Newbler	15	26796622	2014	Only 1 complete genome	ftp://ftp.ncbi.nlm.nih.gov/genomes/all/GCA/000/626/635/GCA_000626635.1_ASM62663v1
Fermentimonas caenicola	ING2-E5B	Unknown	Unknown	Unknown	Unpublished (BU)	2014	Only 1 complete genome	ftp://ftp.ncbi.nlm.nih.gov/genomes/all/GCA/000/953/535/GCA_000953535.1_E5B
Mucinivorans hirudinis	M3T	PacBio + Illumina	HGAP	Unknown	25657285	2014	Only 1 complete genome	ftp://ftp.ncbi.nlm.nih.gov/genomes/all/GCA/000/723/505/GCA_000723505.1_Mucivorans_M3_Nelson-Bomar-Fixed
Odoribacter splanchnicus	DSM 220712	454 + Illumina	Newbler	30	21677857	2011	Only 1 complete genome	ftp://ftp.ncbi.nlm.nih.gov/genomes/all/GCA/000/190/535/GCA_000190535.1_ASM19053v1
Paludibacter propionicigenes	WB4	454 + Illumina	Newbler	30	21475585	2010	Only 1 complete genome	ftp://ftp.ncbi.nlm.nih.gov/genomes/all/GCA/000/183/135/GCA_000183135.1_ASM18313v1
Parabacteroides distasonis	ATCC 8503	Sanger	Phrap + PCAP	13	17579514	2007	Only 1 complete genome	ftp://ftp.ncbi.nlm.nih.gov/genomes/all/GCA/000/012/845/GCA_000012845.1_ASM1284v1
Petrimonas mucosa	ING2-E5A	Unknown	Unknown	Unknown	Unpublished (LIAEPB)	2016	Only 1 complete genome	ftp://ftp.ncbi.nlm.nih.gov/genomes/all/GCA/900/095/795/GCA_900095795.1_E5A
Porphyromonas asaccharolytica	DSM 20707	454 + Illumina	Newbler	30	Unpublished (DOE-JGI)	2011	Only 1 complete genome	ftp://ftp.ncbi.nlm.nih.gov/genomes/all/GCA/000/212/375/GCA_000212375.1_ASM21237v1
Porphyromonas gingivalis	HG66	PacBio	SMRT Analysis	198	25291768	2014	Gap(s) in sequence	ftp://ftp.ncbi.nlm.nih.gov/genomes/all/GCA/000/739/415/GCA_000739415.1_ASM73941v1
Porphyromonas gingivalis	JCVI SC001	Illumina	SPAdes	237	23564253	2013	Gap(s) in sequence	ftp://ftp.ncbi.nlm.nih.gov/genomes/all/GCA/000/380/305/GCA_000380305.1_PgingivalisJCVISC001v1.0
Prevotella dentalis	DSM 3688	454 + Illumina	Newbler	30	Unpublished (BCoM)	2012	Only 1 complete genome	ftp://ftp.ncbi.nlm.nih.gov/genomes/all/GCA/000/242/335/GCA_000242335.3_ASM24233v3
Prevotella denticola	F0289	454 + Illumina	Celera	43	Unpublished (JCVI)	2011	Only 1 complete genome	ftp://ftp.ncbi.nlm.nih.gov/genomes/all/GCA/000/193/395/GCA_000193395.1_ASM19339v1
Prevotella enoeca	F0113	PacBio	HGAP	366	Unpublished (BCoM)	2015	Only 1 complete genome	ftp://ftp.ncbi.nlm.nih.gov/genomes/all/GCA/001/444/445/GCA_001444445.1_ASM144444v1
Prevotella fusca	W1435	PacBio	HGAP	117	Unpublished (BCoM)	2015	Only 1 complete genome	ftp://ftp.ncbi.nlm.nih.gov/genomes/all/GCA/001/262/015/GCA_001262015.1_ASM126201v1
Prevotella intermedia	17	Sanger	Celera	7	Unpublished (JCVI)	2012	More than 1 chromosome	ftp://ftp.ncbi.nlm.nih.gov/genomes/all/GCA/000/261/025/GCA_000261025.1_ASM26102v1
Prevotella intermedia	17-2	PacBio	HGAP	200	26294638	2015	More than 1 chromosome	ftp://ftp.ncbi.nlm.nih.gov/genomes/all/GCA/001/548/195/GCA_001548195.1_ASM154819v1
Prevotella intermedia	ATCC 25611	PacBio	HGAP + Celera	200	Unpublished (FI)	2017	More than 1 chromosome	ftp://ftp.ncbi.nlm.nih.gov/genomes/all/GCA/001/953/955/GCA_001953955.1_ASM195395v1
Prevotella intermedia	strain 17	PacBio	HGAP + Celera	200	Unpublished (FI)	2017	More than 1 chromosome	ftp://ftp.ncbi.nlm.nih.gov/genomes/all/GCA/001/953/935/GCA_001953935.1_ASM195393v1
Prevotella melaninogenica	ATCC 25845	454	Celera	14	Unpublished (HMP)	2010	Only 1 complete genome	ftp://ftp.ncbi.nlm.nih.gov/genomes/all/GCA/000/144/405/GCA_000144405.1_ASM14440v1
Prevotella ruminicola	Bryant 23	Sanger	Unknown	8	20585943	2010	Only 1 complete genome	ftp://ftp.ncbi.nlm.nih.gov/genomes/all/GCA/000/025/925/GCA_000025925.1_ASM2592v1
Prevotella scopos	W2052	Illumina	SPAdes	26	Unpublished (BCoM)	2016	Only 1 complete genome	ftp://ftp.ncbi.nlm.nih.gov/genomes/all/GCA/001/683/355/GCA_001683355.1_ASM168335v1
Proteiniphilum saccharofermentans	M3/6	Unknown	Unknown	Unknown	Unpublished (BU)	2017	Only 1 complete genome	ftp://ftp.ncbi.nlm.nih.gov/genomes/all/GCA/900/095/135/GCA_900095135.1_M36
