# Supplementary material for: Genomic repeats, misassembly and reannotation: a case study with long-read resequencing of Porphyromonas gingivalis reference strains
Source: BMC Genomics. 2018 Jan 16;19:54. doi: 10.1186/s12864-017-4429-4 (PMC5771137; doi:10.1186/s12864-017-4429-4)
Supplement: Supplementary file 13 — CDS/pseudogenes of all strains that have at least two copies in all three P. gingivalis genomes. a. Horizontal histogram of absolute gene counts binned by category. Three of these categories are related to nucleic acids (DNA helicases, regulators mainly containing the HTH 17 domain, and histones), while the remaining ones are related to mobile elements (integrases, transposases, tetracycline-resistance genes, and the tra conjugative transposons). b. Histogram of all transposases coded by genome and divided into CDS and pseudogenes. c. Heatmap table of transposase families separated into coding sequences and pseudogenes. Absolute numbers are presented, as the copy numbers grow, the cell color move from light blue to dark red. (PDF 83 kb) [file 12864_2017_4429_MOESM13_ESM.pdf]

Species	Strain	Sequencing Technology	Assembler	Contigs	Reference Pubmed ID	Release Date	FTP
Bacteroides dorei	CAG:222	Unknown	Unknown	140	Unpublished (TUD)	2013	ftp://ftp.ncbi.nlm.nih.gov/genomes/all/GCA/000/436/135/GCA_000436135.1_MGS222
Bacteroides dorei	5_1_36/D4	454	Newbler	10	Unpublished (HMP)	2009	ftp://ftp.ncbi.nlm.nih.gov/genomes/all/GCA/000/158/335/GCA_000158335.2_Bact_dorei_5_1_36_D4_V2
Bacteroides dorei	CL02T00C15	Illumina	allpaths	22	Unpublished (HMP)	2012	ftp://ftp.ncbi.nlm.nih.gov/genomes/all/GCA/000/273/035/GCA_000273035.1_Bact_dore_CL02T00C15_V1
Bacteroides dorei	CL02T12C06	Illumina	allpaths	21	Unpublished (HMP)	2012	ftp://ftp.ncbi.nlm.nih.gov/genomes/all/GCA/000/273/055/GCA_000273055.1_Bact_dore_CL02T12C06_V1
Bacteroides dorei	CL03T12C01	Illumina	allpaths	20	Unpublished (HMP)	2012	ftp://ftp.ncbi.nlm.nih.gov/genomes/all/GCA/000/273/075/GCA_000273075.1_Bact_dore_CL03T12C01_V1
Bacteroides dorei	DSM 17855	454	Newbler	40	Unpublished (BCoM)	2008	ftp://ftp.ncbi.nlm.nih.gov/genomes/all/GCA/000/156/075/GCA_000156075.1_ASM15607v1
Bacteroides fragilis	CAG:47	Unknown	Unknown	118	Unpublished (TUD)	2013	ftp://ftp.ncbi.nlm.nih.gov/genomes/all/GCA/000/434/095/GCA_000434095.1_MGS47
Bacteroides fragilis	CAG:558	Unknown	Unknown	175	Unpublished (TUD)	2013	ftp://ftp.ncbi.nlm.nih.gov/genomes/all/GCA/000/432/495/GCA_000432495.1_MGS558
Bacteroides fragilis	1007-1-F #10	Illumina	MaSuRCA	83	Unpublished (IGS)	2014	ftp://ftp.ncbi.nlm.nih.gov/genomes/all/GCA/000/598/685/GCA_000598685.2_ASM59868v2
Bacteroides fragilis	1007-1-F #3	Illumina	MaSuRCA	106	Unpublished (IGS)	2014	ftp://ftp.ncbi.nlm.nih.gov/genomes/all/GCA/000/599/265/GCA_000599265.1_ASM59926v1
Bacteroides fragilis	1007-1-F #4	Illumina	MaSuRCA	167	Unpublished (IGS)	2014	ftp://ftp.ncbi.nlm.nih.gov/genomes/all/GCA/000/598/545/GCA_000598545.2_ASM59854v2
Bacteroides fragilis	1007-1-F #5	Illumina	MaSuRCA	157	Unpublished (IGS)	2014	ftp://ftp.ncbi.nlm.nih.gov/genomes/all/GCA/000/601/035/GCA_000601035.1_ASM60103v1
Bacteroides fragilis	1007-1-F #6	Illumina	MaSuRCA	87	Unpublished (IGS)	2014	ftp://ftp.ncbi.nlm.nih.gov/genomes/all/GCA/000/601/095/GCA_000601095.1_ASM60109v1
Bacteroides fragilis	1007-1-F #7	Illumina	MaSuRCA	130	Unpublished (IGS)	2014	ftp://ftp.ncbi.nlm.nih.gov/genomes/all/GCA/000/599/145/GCA_000599145.2_ASM59914v2
Bacteroides fragilis	1007-1-F #8	Illumina	MaSuRCA	315	Unpublished (IGS)	2014	ftp://ftp.ncbi.nlm.nih.gov/genomes/all/GCA/000/598/265/GCA_000598265.1_ASM59826v1
Bacteroides fragilis	1007-1-F #9	Illumina	MaSuRCA	66	Unpublished (IGS)	2014	ftp://ftp.ncbi.nlm.nih.gov/genomes/all/GCA/000/598/885/GCA_000598885.1_ASM59888v1
Bacteroides fragilis	1009-4-F #10	Illumina	MaSuRCA	63	Unpublished (IGS)	2014	ftp://ftp.ncbi.nlm.nih.gov/genomes/all/GCA/000/598/705/GCA_000598705.1_ASM59870v1
Bacteroides fragilis	1009-4-F #7	Illumina	MaSuRCA	46	Unpublished (IGS)	2014	ftp://ftp.ncbi.nlm.nih.gov/genomes/all/GCA/000/599/285/GCA_000599285.2_ASM59928v2
Bacteroides fragilis	14-106904-1	Illumina	SPAdes	73	Poster (Sydenham et al. 2015)	2016	ftp://ftp.ncbi.nlm.nih.gov/genomes/all/GCA/001/816/225/GCA_001816225.1_ASM181622v1
Bacteroides fragilis	2-078382-3	Illumina	SPAdes	140	27348220	2016	ftp://ftp.ncbi.nlm.nih.gov/genomes/all/GCA/001/699/865/GCA_001699865.1_ASM169986v1
Bacteroides fragilis	2-F-2 #4	Illumina	MaSuRCA	213	Unpublished (IGS)	2014	ftp://ftp.ncbi.nlm.nih.gov/genomes/all/GCA/000/598/825/GCA_000598825.1_ASM59882v1
Bacteroides fragilis	2-F-2 #5	Illumina	MaSuRCA	250	Unpublished (IGS)	2014	ftp://ftp.ncbi.nlm.nih.gov/genomes/all/GCA/000/598/285/GCA_000598285.1_ASM59828v1
Bacteroides fragilis	2-F-2 #7	Illumina	MaSuRCA	363	Unpublished (IGS)	2014	ftp://ftp.ncbi.nlm.nih.gov/genomes/all/GCA/000/598/145/GCA_000598145.1_ASM59814v1
Bacteroides fragilis	20656-2-1	Illumina	CLC-WB	68	27348220	2016	ftp://ftp.ncbi.nlm.nih.gov/genomes/all/GCA/001/699/875/GCA_001699875.1_ASM169987v1
Bacteroides fragilis	20793-3	Illumina	MaSuRCA	148	Unpublished (IGS)	2014	ftp://ftp.ncbi.nlm.nih.gov/genomes/all/GCA/000/598/905/GCA_000598905.1_ASM59890v1
Bacteroides fragilis	20793-3	Unknown	Unknown	52	Unpublished*	2016	ftp://ftp.ncbi.nlm.nih.gov/genomes/all/GCA/001/699/855/GCA_001699855.1_ASM169985v1
Bacteroides fragilis	2d2A	Illumina	Unknown	1025	25717097	2015	ftp://ftp.ncbi.nlm.nih.gov/genomes/all/GCA/000/944/095/GCA_000944095.1_2d2A_assembly
Bacteroides fragilis	3-F-2 #6	Illumina	MaSuRCA	201	Unpublished (IGS)	2014	ftp://ftp.ncbi.nlm.nih.gov/genomes/all/GCA/000/598/865/GCA_000598865.1_ASM59886v1
Bacteroides fragilis	320_BFRA	Illumina	Abyss	150	26230489	2015	ftp://ftp.ncbi.nlm.nih.gov/genomes/all/GCA/001/054/865/GCA_001054865.1_ASM105486v1
Bacteroides fragilis	321_BFRA	Illumina	Abyss	150	26230489	2015	ftp://ftp.ncbi.nlm.nih.gov/genomes/all/GCA/001/056/335/GCA_001056335.1_ASM105633v1
Bacteroides fragilis	322_BFRA	Illumina	Abyss	193	26230489	2015	ftp://ftp.ncbi.nlm.nih.gov/genomes/all/GCA/001/054/895/GCA_001054895.1_ASM105489v1
Bacteroides fragilis	3397 N2	Illumina	MaSuRCA	93	Unpublished (IGS)	2014	ftp://ftp.ncbi.nlm.nih.gov/genomes/all/GCA/000/598/565/GCA_000598565.1_ASM59856v1
Bacteroides fragilis	3397 N3	Illumina	MaSuRCA	102	Unpublished (IGS)	2014	ftp://ftp.ncbi.nlm.nih.gov/genomes/all/GCA/000/598/925/GCA_000598925.1_ASM59892v1
Bacteroides fragilis	3397 T10	Illumina	MaSuRCA	2566	Unpublished (IGS)	2014	ftp://ftp.ncbi.nlm.nih.gov/genomes/all/GCA/000/598/405/GCA_000598405.1_ASM59840v1
Bacteroides fragilis	3397 T14	Illumina	MaSuRCA	94	Unpublished (IGS)	2014	ftp://ftp.ncbi.nlm.nih.gov/genomes/all/GCA/000/599/165/GCA_000599165.2_ASM59916v2
Bacteroides fragilis	34-F-2 #13	Illumina	MaSuRCA	426	Unpublished (IGS)	2014	ftp://ftp.ncbi.nlm.nih.gov/genomes/all/GCA/000/598/425/GCA_000598425.1_ASM59842v1
Bacteroides fragilis	3719 A10	Illumina	MaSuRCA	117	Unpublished (IGS)	2014	ftp://ftp.ncbi.nlm.nih.gov/genomes/all/GCA/000/598/845/GCA_000598845.1_ASM59884v1
Bacteroides fragilis	3719 T6	Illumina	MaSuRCA	64	Unpublished (IGS)	2014	ftp://ftp.ncbi.nlm.nih.gov/genomes/all/GCA/000/598/725/GCA_000598725.1_ASM59872v1
Bacteroides fragilis	3725 D9 ii	Illumina	MaSuRCA	91	Unpublished (IGS)	2014	ftp://ftp.ncbi.nlm.nih.gov/genomes/all/GCA/000/699/685/GCA_000699685.1_ASM69968v1
Bacteroides fragilis	3725 D9(v)	Illumina	MaSuRCA	75	Unpublished (IGS)	2014	ftp://ftp.ncbi.nlm.nih.gov/genomes/all/GCA/000/598/585/GCA_000598585.1_ASM59858v1
Bacteroides fragilis	3774 T13	Illumina	MaSuRCA	340	Unpublished (IGS)	2014	ftp://ftp.ncbi.nlm.nih.gov/genomes/all/GCA/000/598/305/GCA_000598305.1_ASM59830v1
Bacteroides fragilis	3783N1-2	Illumina	MaSuRCA	449	Unpublished (IGS)	2014	ftp://ftp.ncbi.nlm.nih.gov/genomes/all/GCA/000/598/325/GCA_000598325.1_ASM59832v1

Species	Strain	Sequencing Technology	Assembler	Contigs	Reference Pubmed ID	Release Date	FTP
Bacteroides fragilis	3783N1-6	Illumina	MaSuRCA	57	Unpublished (IGS)	2014	ftp://ftp.ncbi.nlm.nih.gov/genomes/all/GCA/000/599/065/GCA_000599065.2_ASM59906v2
Bacteroides fragilis	3783N1-8	Illumina	MaSuRCA	85	Unpublished (IGS)	2014	ftp://ftp.ncbi.nlm.nih.gov/genomes/all/GCA/000/598/605/GCA_000598605.1_ASM59860v1
Bacteroides fragilis	3783N2-1	Illumina	MaSuRCA	436	Unpublished (IGS)	2014	ftp://ftp.ncbi.nlm.nih.gov/genomes/all/GCA/000/598/345/GCA_000598345.1_ASM59834v1
Bacteroides fragilis	3976T7	Illumina	MaSuRCA	447	Unpublished (IGS)	2014	ftp://ftp.ncbi.nlm.nih.gov/genomes/all/GCA/000/598/165/GCA_000598165.1_ASM59816v1
Bacteroides fragilis	3976T8	Illumina	MaSuRCA	73	Unpublished (IGS)	2014	ftp://ftp.ncbi.nlm.nih.gov/genomes/all/GCA/000/599/185/GCA_000599185.2_ASM59918v2
Bacteroides fragilis	3986 N(B)19	Illumina	MaSuRCA	792	Unpublished (IGS)	2014	ftp://ftp.ncbi.nlm.nih.gov/genomes/all/GCA/000/598/445/GCA_000598445.1_ASM59844v1
Bacteroides fragilis	3986 N(B)22	Illumina	MaSuRCA	31	Unpublished (IGS)	2014	ftp://ftp.ncbi.nlm.nih.gov/genomes/all/GCA/000/598/945/GCA_000598945.1_ASM59894v1
Bacteroides fragilis	3986 N3	Illumina	MaSuRCA	36	Unpublished (IGS)	2014	ftp://ftp.ncbi.nlm.nih.gov/genomes/all/GCA/000/601/115/GCA_000601115.1_ASM60111v1
Bacteroides fragilis	3986 T(B)13	Illumina	MaSuRCA	44	Unpublished (IGS)	2014	ftp://ftp.ncbi.nlm.nih.gov/genomes/all/GCA/000/598/965/GCA_000598965.1_ASM59896v1
Bacteroides fragilis	3986 T(B)9	Illumina	MaSuRCA	211	Unpublished (IGS)	2014	ftp://ftp.ncbi.nlm.nih.gov/genomes/all/GCA/000/598/465/GCA_000598465.1_ASM59846v1
Bacteroides fragilis	3986T(B)10	Illumina	MaSuRCA	313	Unpublished (IGS)	2014	ftp://ftp.ncbi.nlm.nih.gov/genomes/all/GCA/000/598/185/GCA_000598185.2_ASM59818v2
Bacteroides fragilis	3988 T1	Illumina	MaSuRCA	462	Unpublished (IGS)	2014	ftp://ftp.ncbi.nlm.nih.gov/genomes/all/GCA/000/598/205/GCA_000598205.1_ASM59820v1
Bacteroides fragilis	3988T(B)14	Illumina	MaSuRCA	408	Unpublished (IGS)	2014	ftp://ftp.ncbi.nlm.nih.gov/genomes/all/GCA/000/598/365/GCA_000598365.1_ASM59836v1
Bacteroides fragilis	3996 N(B) 6	Illumina	MaSuRCA	298	Unpublished (IGS)	2014	ftp://ftp.ncbi.nlm.nih.gov/genomes/all/GCA/000/598/225/GCA_000598225.1_ASM59822v1
Bacteroides fragilis	3998 T(B) 4	Illumina	MaSuRCA	424	Unpublished (IGS)	2014	ftp://ftp.ncbi.nlm.nih.gov/genomes/all/GCA/000/598/385/GCA_000598385.1_ASM59838v1
Bacteroides fragilis	3998T(B)3	Illumina	MaSuRCA	444	Unpublished (IGS)	2014	ftp://ftp.ncbi.nlm.nih.gov/genomes/all/GCA/000/598/485/GCA_000598485.1_ASM59848v1
Bacteroides fragilis	3_1_12	454	Unknown	33	Unpublished (HMP)	2009	ftp://ftp.ncbi.nlm.nih.gov/genomes/all/GCA/000/157/015/GCA_000157015.1_ASM15701v1
Bacteroides fragilis	4g8B	Illumina	Unknown	1402	25717097	2015	ftp://ftp.ncbi.nlm.nih.gov/genomes/all/GCA/001/373/095/GCA_001373095.1_4g8B_assembly
Bacteroides fragilis	86-5443-2-2	Illumina	CLC-WB	156	27348220	2016	ftp://ftp.ncbi.nlm.nih.gov/genomes/all/GCA/001/699/885/GCA_001699885.1_ASM169988v1
Bacteroides fragilis	885_BFRA	Illumina	Abyss	435	26230489	2015	ftp://ftp.ncbi.nlm.nih.gov/genomes/all/GCA/001/058/755/GCA_001058755.1_ASM105875v1
Bacteroides fragilis	894_BFRA	Illumina	Abyss	370	26230489	2015	ftp://ftp.ncbi.nlm.nih.gov/genomes/all/GCA/001/058/775/GCA_001058775.1_ASM105877v1
Bacteroides fragilis	915_BFRA	Illumina	Abyss	1290	26230489	2015	ftp://ftp.ncbi.nlm.nih.gov/genomes/all/GCA/001/077/245/GCA_001077245.1_ASM107724v1
Bacteroides fragilis	A7 (UDC12-2)	Illumina	MaSuRCA	87	Unpublished (IGS)	2014	ftp://ftp.ncbi.nlm.nih.gov/genomes/all/GCA/000/598/985/GCA_000598985.1_ASM59898v1
Bacteroides fragilis	ATCC 25285	Illumina	CLC-WB	58	28592550	2017	ftp://ftp.ncbi.nlm.nih.gov/genomes/all/GCA/001/997/325/GCA_001997325.1_ASM199732v1
Bacteroides fragilis	B1 (UDC16-1)	Illumina	MaSuRCA	311	Unpublished (IGS)	2014	ftp://ftp.ncbi.nlm.nih.gov/genomes/all/GCA/000/598/625/GCA_000598625.1_ASM59862v1
Bacteroides fragilis	BF8	Illumina	CLC-WB	5	27246231	2016	ftp://ftp.ncbi.nlm.nih.gov/genomes/all/GCA/001/695/355/GCA_001695355.1_ASM169535v1
Bacteroides fragilis	CL03T00C08	Illumina	allpaths	8	Unpublished (HMP)	2012	ftp://ftp.ncbi.nlm.nih.gov/genomes/all/GCA/000/273/095/GCA_000273095.1_Bact_frag_CL03T00C08_V1
Bacteroides fragilis	CL03T12C07	Illumina	allpaths	7	Unpublished (HMP)	2012	ftp://ftp.ncbi.nlm.nih.gov/genomes/all/GCA/000/273/115/GCA_000273115.1_Bact_frag_CL03T12C07_V1
Bacteroides fragilis	CL05T00C42	Illumina	allpaths	12	Unpublished (HMP)	2012	ftp://ftp.ncbi.nlm.nih.gov/genomes/all/GCA/000/269/525/GCA_000269525.1_PB_Bact_frag_CL05T00C42_V1
Bacteroides fragilis	CL05T00C42	Illumina	allpaths	5	Unpublished (HMP)	2012	ftp://ftp.ncbi.nlm.nih.gov/genomes/all/GCA/000/273/765/GCA_000273765.1_Bact_frag_CL05T00C42_V1
Bacteroides fragilis	CL05T12C13	Illumina	allpaths	13	Unpublished (HMP)	2012	ftp://ftp.ncbi.nlm.nih.gov/genomes/all/GCA/000/273/135/GCA_000273135.1_Bact_frag_CL05T12C13_V1
Bacteroides fragilis	CL07T00C01	Illumina	allpaths	9	Unpublished (HMP)	2012	ftp://ftp.ncbi.nlm.nih.gov/genomes/all/GCA/000/263/115/GCA_000263115.1_Bact_frag_CL07T00C01_V1
Bacteroides fragilis	CL07T12C05	Illumina	allpaths	11	Unpublished (HMP)	2012	ftp://ftp.ncbi.nlm.nih.gov/genomes/all/GCA/000/273/155/GCA_000273155.1_Bact_frag_CL07T12C05_V1
Bacteroides fragilis	DCMOUH0017B	Illumina	SPAdes	232	Poster (Sydenham et al. 2015)	2014	ftp://ftp.ncbi.nlm.nih.gov/genomes/all/GCA/000/710/375/GCA_000710375.2_DCMOUH0017B2.0
Bacteroides fragilis	DCMOUH0018B	Illumina	SPAdes	268	Poster (Sydenham et al. 2015)	2014	ftp://ftp.ncbi.nlm.nih.gov/genomes/all/GCA/000/724/665/GCA_000724665.2_DCMOUH0018B2.0
Bacteroides fragilis	DCMOUH0042B	Illumina	SPAdes	197	Poster (Sydenham et al. 2015)	2014	ftp://ftp.ncbi.nlm.nih.gov/genomes/all/GCA/000/724/795/GCA_000724795.1_DCMOUH0042B1.0
Bacteroides fragilis	DCMOUH0067B	Illumina	SPAdes	343	Poster (Sydenham et al. 2015)	2014	ftp://ftp.ncbi.nlm.nih.gov/genomes/all/GCA/000/724/805/GCA_000724805.1_DCMOUH0067B_1.0
Bacteroides fragilis	DCMOUH0085B	Illumina	SPAdes	157	Poster (Sydenham et al. 2015)	2014	ftp://ftp.ncbi.nlm.nih.gov/genomes/all/GCA/000/724/815/GCA_000724815.1_DCMOUH0085B_1.0
Bacteroides fragilis	DCMSKEJBY0001B	Illumina	SPAdes	213	Poster (Sydenham et al. 2015)	2014	ftp://ftp.ncbi.nlm.nih.gov/genomes/all/GCA/000/710/365/GCA_000710365.2_DCMSKEJBY0001B2.0
Bacteroides fragilis	DS-166	Illumina	MaSuRCA	124	Unpublished (IGS)	2014	ftp://ftp.ncbi.nlm.nih.gov/genomes/all/GCA/000/598/245/GCA_000598245.1_ASM59824v1
Bacteroides fragilis	DS-208	Illumina	MaSuRCA	271	Unpublished (IGS)	2014	ftp://ftp.ncbi.nlm.nih.gov/genomes/all/GCA/000/598/505/GCA_000598505.1_ASM59850v1
Bacteroides fragilis	DS-71	Illumina	MaSuRCA	308	Unpublished (IGS)	2014	ftp://ftp.ncbi.nlm.nih.gov/genomes/all/GCA/000/599/085/GCA_000599085.1_ASM59908v1

Species	Strain	Sequencing Technology	Assembler	Contigs	Reference Pubmed ID	Release Date	FTP
Bacteroides fragilis	Ds-233	Illumina	MaSuRCA	557	Unpublished (IGS)	2014	ftp://ftp.ncbi.nlm.nih.gov/genomes/all/GCA/000/598/805/GCA_000598805.1_ASM59880v1
Bacteroides fragilis	HMW 610	Illumina	allpaths	9	Unpublished (HMP)	2012	ftp://ftp.ncbi.nlm.nih.gov/genomes/all/GCA/000/297/695/GCA_000297695.1_Bact_frag_HMW_610_V1
Bacteroides fragilis	HMW 615	Illumina	allpaths	14	Unpublished (HMP)	2012	ftp://ftp.ncbi.nlm.nih.gov/genomes/all/GCA/000/297/735/GCA_000297735.1_Bact_frag_HMW_615_V1
Bacteroides fragilis	HMW 616	Illumina	allpaths	9	Unpublished (HMP)	2012	ftp://ftp.ncbi.nlm.nih.gov/genomes/all/GCA/000/297/755/GCA_000297755.1_Bact_frag_HMW_616_V1
Bacteroides fragilis	I1345	Illumina	MaSuRCA	76	Unpublished (IGS)	2014	ftp://ftp.ncbi.nlm.nih.gov/genomes/all/GCA/000/598/785/GCA_000598785.2_ASM59878v2
Bacteroides fragilis	J-143-4	Illumina	MaSuRCA	270	Unpublished (IGS)	2014	ftp://ftp.ncbi.nlm.nih.gov/genomes/all/GCA/000/598/525/GCA_000598525.1_ASM59852v1
Bacteroides fragilis	J38-1	Illumina	MaSuRCA	120	Unpublished (IGS)	2014	ftp://ftp.ncbi.nlm.nih.gov/genomes/all/GCA/000/598/645/GCA_000598645.1_ASM59864v1
Bacteroides fragilis	JCM 11017	IonTorrent	Newbler	98	19934255	2014	ftp://ftp.ncbi.nlm.nih.gov/genomes/all/GCA/000/613/425/GCA_000613425.1_ASM61342v1
Bacteroides fragilis	JIM10	454 + IonTorrent	Newbler	81	Unpublished (FMBA)	2016	ftp://ftp.ncbi.nlm.nih.gov/genomes/all/GCA/001/692/695/GCA_001692695.1_ASM169269v1
Bacteroides fragilis	KLE1758	Illumina	Velvet	52	Unpublished (WU)	2016	ftp://ftp.ncbi.nlm.nih.gov/genomes/all/GCA/001/580/095/GCA_001580095.1_ASM158009v1
Bacteroides fragilis	Korea 419	Illumina	MaSuRCA	246	Unpublished (IGS)	2014	ftp://ftp.ncbi.nlm.nih.gov/genomes/all/GCA/000/599/205/GCA_000599205.1_ASM59920v1
Bacteroides fragilis	O:21	Illumina	CLC-WB	14	27246231	2016	ftp://ftp.ncbi.nlm.nih.gov/genomes/all/GCA/001/693/695/GCA_001693695.1_ASM169369v1
Bacteroides fragilis	S13 L11	Illumina	MaSuRCA	790	Unpublished (IGS)	2014	ftp://ftp.ncbi.nlm.nih.gov/genomes/all/GCA/000/599/105/GCA_000599105.1_ASM59910v1
Bacteroides fragilis	S23 R14	Illumina	MaSuRCA	263	Unpublished (IGS)	2014	ftp://ftp.ncbi.nlm.nih.gov/genomes/all/GCA/000/598/665/GCA_000598665.1_ASM59866v1
Bacteroides fragilis	S23L17	Illumina	MaSuRCA	133	Unpublished (IGS)	2014	ftp://ftp.ncbi.nlm.nih.gov/genomes/all/GCA/000/601/055/GCA_000601055.1_ASM60105v1
Bacteroides fragilis	S23L24	Illumina	MaSuRCA	122	Unpublished (IGS)	2014	ftp://ftp.ncbi.nlm.nih.gov/genomes/all/GCA/000/599/305/GCA_000599305.1_ASM59930v1
Bacteroides fragilis	S24L15	Illumina	MaSuRCA	147	Unpublished (IGS)	2014	ftp://ftp.ncbi.nlm.nih.gov/genomes/all/GCA/000/599/005/GCA_000599005.1_ASM59900v1
Bacteroides fragilis	S24L26	Illumina	MaSuRCA	72	Unpublished (IGS)	2014	ftp://ftp.ncbi.nlm.nih.gov/genomes/all/GCA/000/598/745/GCA_000598745.1_ASM59874v1
Bacteroides fragilis	S24L34	Illumina	MaSuRCA	65	Unpublished (IGS)	2014	ftp://ftp.ncbi.nlm.nih.gov/genomes/all/GCA/000/599/325/GCA_000599325.1_ASM59932v1
Bacteroides fragilis	S36L11	Illumina	MaSuRCA	294	Unpublished (IGS)	2014	ftp://ftp.ncbi.nlm.nih.gov/genomes/all/GCA/000/599/125/GCA_000599125.1_ASM59912v1
Bacteroides fragilis	S36L12	Illumina	MaSuRCA	90	Unpublished (IGS)	2014	ftp://ftp.ncbi.nlm.nih.gov/genomes/all/GCA/000/599/345/GCA_000599345.1_ASM59934v1
Bacteroides fragilis	S36L5	Illumina	MaSuRCA	105	Unpublished (IGS)	2014	ftp://ftp.ncbi.nlm.nih.gov/genomes/all/GCA/000/599/025/GCA_000599025.1_ASM59902v1
Bacteroides fragilis	S38L3	Illumina	MaSuRCA	40	Unpublished (IGS)	2014	ftp://ftp.ncbi.nlm.nih.gov/genomes/all/GCA/000/598/765/GCA_000598765.2_ASM59876v2
Bacteroides fragilis	S38L5	Illumina	MaSuRCA	83	Unpublished (IGS)	2014	ftp://ftp.ncbi.nlm.nih.gov/genomes/all/GCA/000/599/365/GCA_000599365.1_ASM59936v1
Bacteroides fragilis	S6L3	Illumina	MaSuRCA	119	Unpublished (IGS)	2014	ftp://ftp.ncbi.nlm.nih.gov/genomes/all/GCA/000/599/225/GCA_000599225.1_ASM59922v1
Bacteroides fragilis	S6L8	Illumina	MaSuRCA	100	Unpublished (IGS)	2014	ftp://ftp.ncbi.nlm.nih.gov/genomes/all/GCA/000/599/385/GCA_000599385.1_ASM59938v1
Bacteroides fragilis	S6R5	Illumina	MaSuRCA	80	Unpublished (IGS)	2014	ftp://ftp.ncbi.nlm.nih.gov/genomes/all/GCA/000/599/045/GCA_000599045.1_ASM59904v1
Bacteroides fragilis	S6R6	Illumina	MaSuRCA	84	Unpublished (IGS)	2014	ftp://ftp.ncbi.nlm.nih.gov/genomes/all/GCA/000/599/245/GCA_000599245.1_ASM59924v1
Bacteroides fragilis	S6R8	Illumina	MaSuRCA	133	Unpublished (IGS)	2014	ftp://ftp.ncbi.nlm.nih.gov/genomes/all/GCA/000/601/075/GCA_000601075.2_ASM60107v2
Bacteroides ovatus	CAG:22	Unknown	Unknown	257	Unpublished (TUD)	2013	ftp://ftp.ncbi.nlm.nih.gov/genomes/all/GCA/000/436/435/GCA_000436435.1_MGS22
Bacteroides ovatus	2789STDY5834943	Illumina	Unknown	57	27144353	2015	ftp://ftp.ncbi.nlm.nih.gov/genomes/all/GCA/001/405/735/GCA_001405735.1_14207_7_66
Bacteroides ovatus	3725 D1 iv	Illumina	MaSuRCA	181	Unpublished (IGS)	2014	ftp://ftp.ncbi.nlm.nih.gov/genomes/all/GCA/000/699/725/GCA_000699725.1_ASM69972v1
Bacteroides ovatus	3725 D9 iii	Illumina	MaSuRCA	556	Unpublished (IGS)	2014	ftp://ftp.ncbi.nlm.nih.gov/genomes/all/GCA/000/699/665/GCA_000699665.1_ASM69966v1
Bacteroides ovatus	3_8_47FAA	454	Newbler	25	Unpublished (HMP)	2011	ftp://ftp.ncbi.nlm.nih.gov/genomes/all/GCA/000/218/325/GCA_000218325.1_Bact_ovat_3_8_47FAA_V1
Bacteroides ovatus	ATCC 8483	454 + Sanger	Newbler + PCAP	32	Unpublished (HMP)	2007	ftp://ftp.ncbi.nlm.nih.gov/genomes/all/GCA/000/154/125/GCA_000154125.1_ASM15412v1
Bacteroides ovatus	CL02T12C04	Illumina	allpaths	15	Unpublished (HMP)	2012	ftp://ftp.ncbi.nlm.nih.gov/genomes/all/GCA/000/273/195/GCA_000273195.1_Bact_ovat_CL02T12C04_V1
Bacteroides ovatus	CL03T12C18	Illumina	allpaths	19	Unpublished (HMP)	2012	ftp://ftp.ncbi.nlm.nih.gov/genomes/all/GCA/000/273/215/GCA_000273215.1_Bact_ovat_CL03T12C18_V1
Bacteroides ovatus	CL09T03C03	Illumina	Velvet	193	26768901	2016	ftp://ftp.ncbi.nlm.nih.gov/genomes/all/GCA/001/535/615/GCA_001535615.1_ASM153561v1
Bacteroides ovatus	DSM 1896	Unknown	Unknown	60	Unpublished (DOE-JGI)	2016	ftp://ftp.ncbi.nlm.nih.gov/genomes/all/GCA/900/107/475/GCA_900107475.1_IMG-taxon_2693429857_annotated_assembly
Bacteroides ovatus	KLE1656	Illumina	Velvet	87	Unpublished (WU)	2016	ftp://ftp.ncbi.nlm.nih.gov/genomes/all/GCA/001/578/575/GCA_001578575.1_ASM157857v1
Bacteroides ovatus	NLAE-zl-C500	Unknown	Unknown	125	Unpublished (DOE-JGI)	2016	ftp://ftp.ncbi.nlm.nih.gov/genomes/all/GCA/900/102/645/GCA_900102645.1_IMG-taxon_2654588143_annotated_assembly
Bacteroides ovatus	NLAE-zl-C57	Unknown	Unknown	174	Unpublished (DOE-JGI)	2016	ftp://ftp.ncbi.nlm.nih.gov/genomes/all/GCA/900/100/465/GCA_900100465.1_IMG-taxon_2654588146_annotated_assembly

Species	Strain	Sequencing Technology	Assembler	Contigs	Reference Pubmed ID	Release Date	FTP
Bacteroides ovatus	SD CMC 3f	454	Newbler	156	Unpublished (JCVI)	2010	ftp://ftp.ncbi.nlm.nih.gov/genomes/all/GCA/000/178/275/GCA_000178275.1_ASM17827v1
Bacteroides thetaiotaomicron	CAG:40	Unknown	Unknown	142	Unpublished (TUD)	2013	ftp://ftp.ncbi.nlm.nih.gov/genomes/all/GCA/000/437/995/GCA_000437995.1_MGS40
Bacteroides thetaiotaomicron	3731	Unknown	Unknown	175	Unpublished (WU)	2015	ftp://ftp.ncbi.nlm.nih.gov/genomes/all/GCA/001/049/535/GCA_001049535.1_3731
Bacteroides thetaiotaomicron	7330	Unknown	Unknown	407	Unpublished (WU)	2015	ftp://ftp.ncbi.nlm.nih.gov/genomes/all/GCA/001/049/555/GCA_001049555.1_7330
Bacteroides thetaiotaomicron	14-106904-2	Illumina	SPAdes	102	Poster (Sydenham et al. 2015)	2016	ftp://ftp.ncbi.nlm.nih.gov/genomes/all/GCA/001/816/245/GCA_001816245.1_ASM181624v1
Bacteroides thetaiotaomicron	19_BTHE	Illumina	Abyss	351	26230489	2015	ftp://ftp.ncbi.nlm.nih.gov/genomes/all/GCA/001/055/755/GCA_001055755.1_ASM105575v1
Bacteroides thetaiotaomicron	2789STDY5608873	Illumina	Unknown	31	27144353	2015	ftp://ftp.ncbi.nlm.nih.gov/genomes/all/GCA/001/405/255/GCA_001405255.1_13414_6_57
Bacteroides thetaiotaomicron	2789STDY5834846	Illumina	Unknown	63	27144354	2015	ftp://ftp.ncbi.nlm.nih.gov/genomes/all/GCA/001/405/095/GCA_001405095.1_13470_2_65
Bacteroides thetaiotaomicron	2789STDY5834899	Illumina	Unknown	52	27144355	2015	ftp://ftp.ncbi.nlm.nih.gov/genomes/all/GCA/001/405/895/GCA_001405895.1_14207_7_22
Bacteroides thetaiotaomicron	2789STDY5834945	Illumina	Unknown	28	27144356	2015	ftp://ftp.ncbi.nlm.nih.gov/genomes/all/GCA/001/406/075/GCA_001406075.1_14207_7_68
Bacteroides thetaiotaomicron	2e6A	Unknown	Unknown	1730	Unpublished (WU)	2015	ftp://ftp.ncbi.nlm.nih.gov/genomes/all/GCA/001/373/135/GCA_001373135.1_2e6A_assembly
Bacteroides thetaiotaomicron	3a5B	Unknown	Unknown	2372	Unpublished (WU)	2015	ftp://ftp.ncbi.nlm.nih.gov/genomes/all/GCA/000/937/835/GCA_000937835.1_3a5B_assembly
Bacteroides thetaiotaomicron	KLE1254	Illumina	Velvet	108	Unpublished (WU)	2016	ftp://ftp.ncbi.nlm.nih.gov/genomes/all/GCA/001/578/565/GCA_001578565.1_ASM157856v1
Bacteroides thetaiotaomicron	KPPR-3	Unknown	Unknown	38	Unpublished (DOE-JGI)	2016	ftp://ftp.ncbi.nlm.nih.gov/genomes/all/GCA/900/109/385/GCA_900109385.1_IMG-taxon_2593339226_annotated_assembly
Bacteroides thetaiotaomicron	dnLKV9	Illumina	allpaths	6	Unpublished (BI)	2013	ftp://ftp.ncbi.nlm.nih.gov/genomes/all/GCA/000/403/155/GCA_000403155.2_Bact_thet_dnLKV9_V1
Porphyromonas gingivalis	11A	Illumina	Velvet	89	28184216	2017	ftp://ftp.ncbi.nlm.nih.gov/genomes/all/GCA/900/157/295/GCA_900157295.1_11A
Porphyromonas gingivalis	13_1	Illumina	Velvet	68	28184216	2017	ftp://ftp.ncbi.nlm.nih.gov/genomes/all/GCA/900/157/285/GCA_900157285.1_13-1
Porphyromonas gingivalis	3A1	Illumina	Velvet	56	28184216	2017	ftp://ftp.ncbi.nlm.nih.gov/genomes/all/GCA/900/157/325/GCA_900157325.1_3A1
Porphyromonas gingivalis	3_3	Illumina	Velvet	72	28184216	2017	ftp://ftp.ncbi.nlm.nih.gov/genomes/all/GCA/900/157/215/GCA_900157215.1_Strain_3-3
Porphyromonas gingivalis	7BTORR	Illumina	Velvet	72	28184216	2017	ftp://ftp.ncbi.nlm.nih.gov/genomes/all/GCA/900/157/225/GCA_900157225.1_7BTORR
Porphyromonas gingivalis	84_3	Illumina	Velvet	50	28184216	2017	ftp://ftp.ncbi.nlm.nih.gov/genomes/all/GCA/900/157/275/GCA_900157275.1_84-3
Porphyromonas gingivalis	A7A1_28	Illumina	Velvet	22	28184216	2017	ftp://ftp.ncbi.nlm.nih.gov/genomes/all/GCA/900/157/265/GCA_900157265.1_A7A1-28
Porphyromonas gingivalis	AFR5B1	Illumina	Velvet	88	28184216	2017	ftp://ftp.ncbi.nlm.nih.gov/genomes/all/GCA/900/157/345/GCA_900157345.1_AFR-5B1
Porphyromonas gingivalis	ATCC 49417	Illumina	Velvet	77	28184216	2017	ftp://ftp.ncbi.nlm.nih.gov/genomes/all/GCA/900/157/255/GCA_900157255.1_ATCC49417
Porphyromonas gingivalis	Ando	Illumina	Velvet	112	26543123	2015	ftp://ftp.ncbi.nlm.nih.gov/genomes/all/GCA/001/297/745/GCA_001297745.1_ASM129774v1
Porphyromonas gingivalis	F0185	Illumina	Velvet	113	Unpublished (HMP)	2013	ftp://ftp.ncbi.nlm.nih.gov/genomes/all/GCA/000/467/955/GCA_000467955.1_ASM46795v1
Porphyromonas gingivalis	F0566	Illumina	Velvet	192	Unpublished (HMP)	2013	ftp://ftp.ncbi.nlm.nih.gov/genomes/all/GCA/000/467/975/GCA_000467975.1_ASM46797v1
Porphyromonas gingivalis	F0568	Illumina	Velvet	154	Unpublished (HMP)	2013	ftp://ftp.ncbi.nlm.nih.gov/genomes/all/GCA/000/467/795/GCA_000467795.1_ASM46779v1
Porphyromonas gingivalis	F0569	Illumina	Velvet	111	Unpublished (HMP)	2013	ftp://ftp.ncbi.nlm.nih.gov/genomes/all/GCA/000/467/815/GCA_000467815.1_ASM46781v1
Porphyromonas gingivalis	F0570	Illumina	Velvet	117	Unpublished (HMP)	2013	ftp://ftp.ncbi.nlm.nih.gov/genomes/all/GCA/000/467/835/GCA_000467835.1_ASM46783v1
Porphyromonas gingivalis	MP4-504	Illumina	SPAdes	92	27056232	2016	ftp://ftp.ncbi.nlm.nih.gov/genomes/all/GCA/001/553/795/GCA_001553795.1_ASM155379v1
Porphyromonas gingivalis	SJD2	Illumina	SOAPdenovo	117	24385574	2013	ftp://ftp.ncbi.nlm.nih.gov/genomes/all/GCA/000/503/975/GCA_000503975.1_SJD2
Porphyromonas gingivalis	SU60	Illumina	Velvet	53	28184216	2017	ftp://ftp.ncbi.nlm.nih.gov/genomes/all/GCA/900/157/235/GCA_900157235.1_YH522
Porphyromonas gingivalis	W4087	Illumina	Velvet	114	Unpublished (HMP)	2013	ftp://ftp.ncbi.nlm.nih.gov/genomes/all/GCA/000/467/995/GCA_000467995.1_ASM46799v1
Porphyromonas gingivalis	W50	Illumina	Celera	104	Unpublished (JCVI)	2012	ftp://ftp.ncbi.nlm.nih.gov/genomes/all/GCA/000/271/945/GCA_000271945.1_PgingivalisW50v1.0
Tannerella forsythia	9610	Illumina	SPAdes	79	Unpublished (WU)	2017	ftp://ftp.ncbi.nlm.nih.gov/genomes/all/GCA/001/938/785/GCA_001938785.1_ASM193878v1
Tannerella forsythia	ATCC 43037	Illumina	SPAdes	141	26067981	2015	ftp://ftp.ncbi.nlm.nih.gov/genomes/all/GCA/001/006/485/GCA_001006485.1_ASM100648v1
Tannerella forsythia	UB20	Illumina	Unknown	93	Unpublished (UoS)	2016	ftp://ftp.ncbi.nlm.nih.gov/genomes/all/GCA/900/096/735/GCA_900096735.1_TFUB20
Tannerella forsythia	UB22	Illumina	Unknown	98	Unpublished (UoS)	2016	ftp://ftp.ncbi.nlm.nih.gov/genomes/all/GCA/900/096/715/GCA_900096715.1_TFUB22
Tannerella forsythia	UB4	Illumina	Unknown	71	Unpublished (UoS)	2016	ftp://ftp.ncbi.nlm.nih.gov/genomes/all/GCA/900/096/725/GCA_900096725.1_TFUB4
