# Supplementary material for: Genomic repeats, misassembly and reannotation: a case study with long-read resequencing of Porphyromonas gingivalis reference strains
Source: BMC Genomics. 2018 Jan 16;19:54. doi: 10.1186/s12864-017-4429-4 (PMC5771137; doi:10.1186/s12864-017-4429-4)
Supplement: Supplementary file 14 — Overview of the core genomes of P. gingivalis strains ATCC 33277, TDC60, and W83. a. Pie chart of genes present in all three strains grouped by categories: constant (more than 97% nucleotide identity, none has paralogs); variable (less than 97% nucleotide identity, none have paralogs); with paralogs (at least one strain has paralogs); and with pseudogenisation (at least one strain has a pseudogene, another a functional CDS, and none have paralogs). b. Constant core genes classified into five categories. c. Genes in variable core genome. The 47 genes are presented grouped by function. d. Core genes with paralogs. Gene names and products are listed, and the number of paralogs detailed by strain. To facilitate reading, cells were shaded when at least two paralogs exist. *, pseudogenes; †, a hypothetical gene clustered with genes from the BF0131 conjugative transposon. (PDF 26 kb) [file 12864_2017_4429_MOESM14_ESM.pdf]

Strain	CDS to pseudogene	Pseudogene to CDS	Fusion	Separation	Strand change
**ATCC 33277**	18	13	3 CDS -> 1 pseudogene	1 pseudogene -> 2 CDS	1 CDS -> 1 CDS<br>1 pseudogene -> 1 CDS
			2 pseudogene -> 1 pseudogene		
			8 CDS -> 4 pseudogene		
			2 CDS + 1 pseudogene -> 1 pseudogene		
			4 CDS + 4 pseudogene -> 4 pseudogene		
			1 CDS + 1 pseudogene -> 1 CDS		
			2 CDS -> 1 CDS		
**TDC60**	15	8	8 CDS -> 4 pseudogene	1 pseudogene -> 2 CDS	1 pseudogene -> 1 CDS
			2 pseudogene -> 1 CDS		
**W83**	16	7	16 CDS -> 8 pseudogene	—	2 CDS -> 2 CDS
			2 pseudogene + 1 CDS -> 1 pseudogene		
			1 pseudogene + 1 CDS -> 1 pseudogene		
