# Supplementary material for: Genomic repeats, misassembly and reannotation: a case study with long-read resequencing of Porphyromonas gingivalis reference strains
Source: BMC Genomics. 2018 Jan 16;19:54. doi: 10.1186/s12864-017-4429-4 (PMC5771137; doi:10.1186/s12864-017-4429-4)
Supplement: Supplementary file 15 — Core genes with pseudogenisation. Gene names and products are listed for all 36 genes that are present in this subset of core genes. An asterisk indicates the strains in which they are pseudogenised, and genes with pseudogenes in more than one strain are bold. (PDF 35 kb) [file 12864_2017_4429_MOESM15_ESM.pdf]

	Macromolecules and ion transport and metabolism			Nucleic Acids & protein metabolism			Related to mobile elements			Proteins with subcellular localization or known domains			Hypothetical proteins		
Strain	ATCC 33277	TDC	W83	ATCC 33277	TDC	W83	ATCC 33277	TDC	W83	ATCC 33277	TDC	W83	ATCC 33277	TDC	W83
New pseudogenes	0	0	0	0	0	0	**8**	2	**5**	1	0	1	0	0	0
New CDS	5	1	1	12	2	2	31	2	**9**	1	1	0	**6**	0	0
CDS to pseudogenes	3	1	2	2	**5**	2	13	**8**	**9**	0	1	1	0	0	2
Pseudogenes to CDS	3	3	4	**6**	1	1	3	3	2	0	1	0	1	0	0
Fusions	**7**	2	**5**	0	0	1	5	2	2	1	1	1	0	0	1
Separations	0	0	0	2	2	0	0	0	0	0	0	0	0	0	0
Coding Strand Change	0	0	0	0	0	0	0	0	0	1	0	1	1	1	1
Sub-Total	18	7	12	22	10	6	60	17	27	4	4	4	8	1	4
