# Supplementary material for: Genomic repeats, misassembly and reannotation: a case study with long-read resequencing of Porphyromonas gingivalis reference strains
Source: BMC Genomics. 2018 Jan 16;19:54. doi: 10.1186/s12864-017-4429-4 (PMC5771137; doi:10.1186/s12864-017-4429-4)
Supplement: Supplementary file 16 — CDS/pseudogene differences by strain. Comparison of the publically available annotations accessed via NCBI versus this study’s annotation showed that coding sequences changed to pseudogenes, pseudogenes changed to CDS, features fused, single features split into two, and coding strands changed. See the Results for information on shared CDS and pseudogenes, eliminated CDS/genes, and all other features (tRNA, rRNA, ncRNA, tmRNA, riboswitches, mobile elements, signal peptides, and repeat regions). (PDF 66 kb) [file 12864_2017_4429_MOESM16_ESM.pdf]

gene	product	ATCC 33277	TDC60	W83
araC	arabinose operon regulatory protein			*
bepA_4	**Beta-barrel assembly-enhancing protease**	*	*	
cas1_2	CRISPR-associated protein Cas1	*		
cirA_2	TonB-dependent receptor putative	*		
clcA	H(+)/Cl(-) exchange transporter			*
cobQ	cobyric acid synthase			*
cshA_1	ATP-dependent RNA helicase		*	
drsE	Coenzyme A disulfide reductase	*		
DUF3078_1	hypothetical protein	*		
feuA	periplasmic binding protein			*
fib3	fibronectin type III domain protein			*
FIG00936174	hypothetical protein	*		
fimB	fimbriae length regulator	*		
fimS	two-component sensor histidine kinase			*
fucP	L-fucose-proton symporter		*	
glkA	**glucokinase**	*		*
GSCFA	hypothetical protein			*
haeS	two-component sensor histidine kinase	*		
hipB_1	transcriptional regulator		*	
lipo_03	**lipoprotein FIG00936185**	*	*	
mepM	Murein DD-endopeptidase	*		
mfa1	fimbrillin protein			*
mfa5	fimbrillin associated protein			*
nahK	N-acetylhexosamine 1-kinase			*
natA_1	ABC transporter ATP-binding protein			*
nrfA	cytochrome c nitrite reductase catalytic subunit	*		
nrfB	cytochrome c biogenesis protein			*
ogt	Methylated-DNA-protein-cysteine methyltransferase	*		
omp_09	outer membrane protein immunoreactive 23 kDa antigen			*
PAD_porph_4	peptidylarginine deiminase		*	
PF07877	PF07877 family protein			*
pfeA_1	ferric enterobactin receptor			*
sprA	gliding motility protein			*
wapA_2	tRNA(Glu)-specific nuclease			*
ydaF_1	ribosomal N-acetyltransferase		*	
yqhD	**Fe-dependent oxidoreductase alcohol dehydrogenase**		*	*
**Total**		**13**	**8**	**19**
